# The Real‐World Safety Profile of Immune Checkpoint Inhibitors in Melanoma: FAERS Pharmacovigilance Analysis Complemented by Randomized Trial Evidence

**DOI:** 10.1002/hsr2.72865

**Published:** 2026-07-28

**Authors:** Yiwei Chen, Xueyan Liang, Xiaoyu Chen, Yan Li

**Affiliations:** ^1^ Department of Pharmacy Guangxi Academy of Medical Sciences and the People's Hospital of Guangxi Zhuang Autonomous Region Nanning People's Republic of China; ^2^ Phase 1 Clinical Trial Laboratory, Guangxi Academy of Medical Sciences and the People's Hospital of Guangxi Zhuang Autonomous Region Nanning People's Republic of China; ^3^ Department of Scientific Research and Discipline Development Guangxi Academy of Medical Sciences and the People's Hospital of Guangxi Zhuang Autonomous Region Nanning People's Republic of China

**Keywords:** FAERS, immune checkpoint inhibitors, melanoma, pharmacovigilance, real‐world study

## Abstract

**Background and Aims:**

Melanoma can be effectively treated with immune checkpoint inhibitors (ICIs), but information regarding the risks of treatment‐related adverse events (trAEs), immune‐related adverse events (irAEs), and real‐world adverse events (AEs) is scarce.

**Methods:**

We searched PubMed, Embase, Medline, and Cochrane CENTRAL databases for randomized controlled trials (RCTs) comparing ICIs for melanoma therapy up to February 16, 2025. Bayesian network meta‐analysis (NMA) was conducted, and odds ratios (ORs) with 95% credible intervals (95%CrI) were calculated. A pharmacovigilance analysis was performed using the Food and Drug Administration Adverse Event Reporting System (FAERS) database. ICI‐associated AEs in patients with melanoma were obtained for the period from the first quarter (Q1) of 2004 to Q1 of 2023.

**Results:**

The NMA included 31 RCTs, comprising 11,937 patients with melanoma. Ipilimumab (Ipi), nivolumab (Nivo), and pembrolizumab (Pem) were ranked as having the lowest risk of any grade as well as grade 3–5 trAEs. Chemotherapy (Chemo) was associated with the lowest risk of any grade or grade 3–5 irAEs. Compared to other ICIs, Chemo, Ipi, and Ipi‐Chemo were associated with the lowest risk of any irAEs. Based on the results of the FAERS pharmacovigilance analysis, we identified 5974 AEs in 4599 melanoma patients treated with ICIs. ICIs were associated with statistically significant indications involving 236 preferred terms (PTs). Thoroughly examined and pinpointed AEs were strongly correlated with ICIs. Pem treatment was associated with 166 significant PTs. Apart from malignant neoplasm progression and death, the most common PT was an immune‐mediated adverse reaction.

**Conclusion:**

The results of this study indicated that ICI safety profiles vary. These findings provide comparative safety signals that may help inform AE monitoring and risk assessment in patients with melanoma receiving ICIs, but they should not be interpreted as definitive prescribing guidance.

## 1 Introduction

Melanoma is a severe skin cancer that poses a major public health challenge. In Europe, North America, and Oceania, the incidence of melanoma has been escalating more rapidly than that of any other cancer over the past four decades [1]. The 5‐year survival rate for early‐stage melanomas (stages I and II) is high (98.4%) [2]. However, the 3‐year survival rate for advanced melanoma (stages III and IV) in patients receiving chemotherapy (Chemo) is only 12.2% [3, 4]. In recent years, immune checkpoint inhibitors (ICIs) have become the favored initial treatment for patients with advanced melanoma, with approximately half of patients experiencing durable antitumor responses. The use of ICIs targeting programmed death‐1/ligand‐1 (PD‐1/PD‐L1) prolongs the survival of patients with melanoma [4, 5]. Anti‐programmed cell death‐1 (anti‐PD‐1) stimulates the widespread activation of T cells and causes autoimmune effects, such as immune‐related adverse events (irAEs), which affect multiple organs.

A previously published network meta‐analysis (NMA) primarily focused on irAEs associated with ICI therapy [2]. However, no studies to date have explored irAEs related to system organ classes (SOC) and treatment‐related adverse events (trAEs). Moreover, the majority of previously published studies have not focused on the risk of treatment‐related adverse events (trAEs) linked to various ICI regimens, which may fluctuate depending on the treatment strategy. Additionally, real‐world pharmacovigilance data from actual clinical data were not assessed concurrently. Information on real‐world adverse events (AEs) in patients with melanoma who are treated with ICI regimens is lacking. We thus performed an NMA to evaluate both irAEs and trAEs, given that no randomized controlled trials (RCTs) have directly compared different ICI strategies. We also scrutinized real‐world pharmacovigilance data from the Food and Drug Administration (FDA) Adverse Event Reporting System (FAERS), focusing on reported adverse events (AEs) associated with ICI use in clinical settings.

## 2 Methods

### 2.1 Study Design

An NMA was performed to ascertain the risks related to trAEs and irAEs in patients with melanoma who were treated with ICIs, based on RCT data. Moreover, the FAERS database was used to assess AE reporting signals in melanoma patients who received ICIs. In this study, we defined trAEs and irAEs based on AE reporting in RCTs. Real‑world AEs were obtained from the FAERS database.

### 2.2 Systematic Review Procedures

#### 2.2.1 Data Sources and Searches

The NMA was performed according to a previously developed protocol (PROSPERO CRD42023494136) and followed the PRISMA guidelines [6]. We searched the PubMed, Embase, Medline, and Cochrane CENTRAL databases, without language restrictions, for suitable RCT reports, up to February 16, 2025.

#### 2.2.2 Study Selection and Data Extraction

Search terms related to ICIs, such as nivolumab (Nivo) and pembrolizumab (Pem), together with terms related to randomized controlled trials, were used in the database searches. RCTs that evaluated ICIs as monotherapy or in combination with placebo or active controls in patients with melanoma and that reported AE outcomes were eligible for inclusion. Data extraction was independently performed by two authors. Disagreements were resolved by a third reviewer. For each included study, we recorded the following results: (1) details about the study (such as phase, registered, and published information); (2) characteristics of patients (such as sex, age, and histology), and (3) interventions and outcomes (such as cohort size, therapeutic strategies used, and the number of different trAEs and irAEs).

Two authors independently assessed the risk‐of‐bias in the included studies by using the risk‐of‐bias tool 2.0 [7].

#### 2.2.3 Outcome Measures

In this study, the primary outcomes were any trAEs or irAEs (grades 1–5), and severe trAEs or irAEs (grades 3–5) [8, 9]. The five levels of AEs were defined as mild‐to‐moderate AEs (grades 1 and 2), severe or medically significant but not immediate AEs (grade 3), life‐threatening AEs (grade 4), and AEs resulting in death (grade 5) [9, 10]. In the NMA, the trAE and irAE data were characterized as reported in each RCT. The incidence and severity of trAEs and irAEs varied significantly according to the therapeutic regimen. Secondary outcomes included trAEs or irAEs specific to each SOC as well as outcomes based on the severity of the AEs. AEs were normalized using the Medical Dictionary for Regulatory Activities (MedDRA), which includes 27 SOCs [11]. Thus, AEs in each report were classified according to their associated SOC levels using MedDRA (version 25.0).

#### 2.2.4 Data Synthesis and Statistical Analysis

Bayesian NMA data synthesis was conducted using the “gemtc” package in R (R Foundation for Statistical Computing, Vienna, Austria). The “netmeta” package was used to create network plots for the various treatment regimens. We conducted a Markov chain Monte Carlo simulation with vague priors across the four chains. Non‐informative priors were applied to the treatment effects, specified as normal distributions centered at zero with a large variance, to ensure that the posterior estimates were primarily driven by the observed data rather than by prior assumptions. The convergence of the Bayesian models was evaluated using Gelman–Rubin diagnostics, with all potential scale reduction factors approaching 1.0, and by visual inspection of trace plots across four independent chains (burn‐in of 50,000 iterations, followed by 500,000 sampling iterations, with thinning set to 10). The results of the NMA are presented as odds ratios (ORs) and 95% credible intervals (CrIs). The surface under the cumulative ranking curve (SUCRA) was calculated to illustrate the ranking of interventions. When the synthesis of data in the trials included in the NMA was not feasible, a narrative review was employed. The regimen with the lowest risk of AE was considered to be the optimal choice among all treatments. Therefore, we conducted a pairwise meta‐analysis of different RCTs greater than based on a frequentist approach and compared the corresponding pooled results derived from Bayesian models for the primary outcomes. The Q test and statistical inconsistency index (*I* ^2^) were used to evaluate statistical heterogeneity and inconsistency. When *I* ^2^ was greater than 50%, the level of heterogeneity was considered substantial, which prompted the implementation of a sensitivity analysis to identify the source of heterogeneity [12].

### 2.3 Pharmacovigilance Study Procedures

#### 2.3.1 Data Source

In this study on drug safety, we scrutinized the AEs associated with ICIs in patients with melanoma. This analysis was conducted using the publicly available FAERS database, which is a comprehensive resource for safety reports [13]. We used ICIs and melanoma as search terms to extract reported data related to ICIs and melanoma from the FAERS database. The data spanned the period from the first quarter of 2004 to the first quarter of 2023. Our study only incorporated instances where ICIs were the primary suspects for the treatment of melanoma patients. The FAERS database also assigns codes to reported AEs according to the preferred term (PT) codes from MedDRA. The codes were arranged systematically into five tiers. PTs are unique representations of specific medical concepts. This hierarchical structure also encompasses “High‐Level Terms” (HLTs) and “High‐Level Group Terms” (HLGTs). SOCs are categorized into the causes, locations of manifestations, and objectives of HLGTs. We utilized the MedDRA and extracted PTs with a primary SOC marked as “Yes” for analysis.

The FAERS data were cleaned by removing incomplete reports lacking essential information (e.g., drug name and event date). Duplicate entries were excluded based on the case ID and report date. We filtered data for AEs in which ICIs were identified as the primary suspect for melanoma treatment, and excluded irrelevant or concomitant drug reports. The extracted signals were validated by cross‑referencing with published literature and drug‐labeling information to ensure consistency.

#### 2.3.2 Signal Mining

Signal mining is a method frequently employed in pharmacovigilance research to examine the possible links between the various PTs of AE and specific drugs. These potential associations can subsequently be clinically assessed by reviewing individual reports [14]. The reporting odds ratio (ROR) is a statistical measure used in pharmacovigilance [15] that compares the probability of a specific event occurring with a particular medicinal product with the probability of the same event occurring with all other medicinal products in the database. In this study, the ROR was computed to assess ICI‐related AE signals in patients with melanoma [16]. We performed a disproportionality analysis using the complete AE reports of patients with melanoma from the FAERS database as a reference in order to assess the potential association between AEs and different ICIs in patients with melanoma [17]. We constructed a contingency table for the drug AEs, then computed the ROR based on this table [18], prior to analyzing disproportionality of ICI‐related AEs. AE signals were deemed to be associated with various types of ICIs if a minimum of three cases of AEs were found at the PT level. Additionally, the lower limit of the 95% confidence interval (CI) for the ROR had to be greater than 1.

#### 2.3.3 Descriptive Analysis

We analyzed the clinical attributes of patients with melanoma as a consequence of ICI‐related AEs. These attributes included factors such as age, sex, country, FDA acceptance year, ICI type, and priority of the case [9]. Outcomes that were classified as serious included death, disability, and life‐threatening situations. Data importation and analysis were performed using PostgreSQL (version 14) and R, using the methodologies outlined in previous research [18].

## 3 Results

### 3.1 Characteristics and Quality of Included Studies

The database searches identified 3785 articles, of which 31 RCTs, encompassing a total of 11,937 patients, were included in the NMA (Figure S1). The comprehensive characteristics of the included studies are summarized in Table S1 [3, 19, 20, 21, 22, 23, 24, 25, 26, 27, 28, 29, 30, 31, 32, 33, 34, 35, 36, 37, 38, 39, 40, 41, 42, 43, 44, 45, 46, 47, 48]. Sixteen of the included studies demonstrated a low risk of bias. However, other studies exhibited a risk‐of‐bias categorized as “some concerns” in relation to randomization and deviation from the planned intervention. Details are presented in Table S2.

### 3.2 Network Meta‐Analysis Outcomes

#### 3.2.1 Primary Outcomes

**Figure 1:**
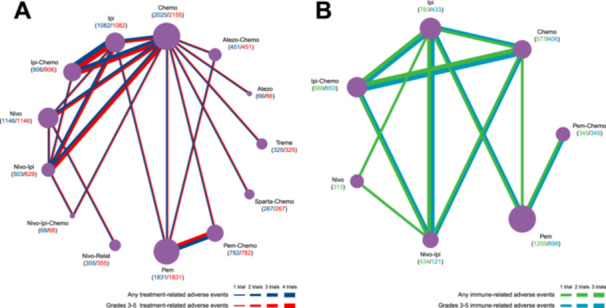
Network plots of adverse events of ICIs for melanoma. (A) Comparisons were performed on any and grades 3–5 treatment‐related adverse events. (B) Comparisons were performed on any‐grade and grade 3–5 immune‐related adverse events. Atezo, atezolizumab; Chemo, chemotherapy; Ipi, ipilimumab; Nivo, nivolumab; Pem, pembrolizumab; Relat, relatlimab; Sparta, spartalizumab; Treme, tremelimumab.

**Figure 2:**
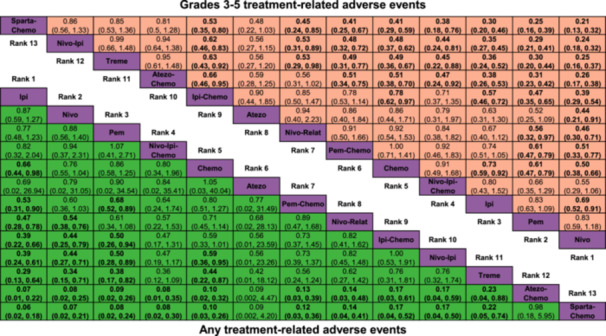
Odds ratio (95% CrI) estimated from Bayesian network meta‐analysis of any and grades 3–5 treatment‐related adverse events associated with each treatment regimen. Atezo, atezolizumab; Chemo, chemotherapy; Ipi, ipilimumab; Nivo, nivolumab; Pem, pembrolizumab; Relat, relatlimab; Sparta, spartalizumab; Treme, tremelimumab.

**Figure 3:**
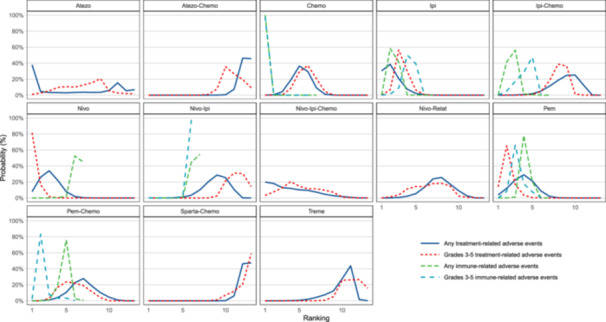
Ranking of the probability estimated from Bayesian network meta‐analysis of being the lowest risk treatment regimen. Atezo, atezolizumab; Chemo, chemotherapy; Ipi, ipilimumab; Nivo, nivolumab; Pem, pembrolizumab; Relat, relatlimab; Sparta, spartalizumab; Treme, tremelimumab.

Network plots for any grade or grades 3–5 trAEs are presented in Figure 1A. Comparisons of ORs and 95% CrIs are depicted in Figure 2. The rank probability for each ICI is illustrated in Figure 3. In terms of any grade and grades 3–5 trAEs, ipilimumab (Ipi), Nivo, and Pem were associated with the lowest risk of trAEs, and were ranked as the safest ICIs for patients with melanoma. These ICIs were associated with ORs ranging from 0.02 to 0.98. In contrast, spartalizumab combined with chemotherapy (Sparta‐Chemo) was considered to be one of the least safe drugs.

**Figure 4:**
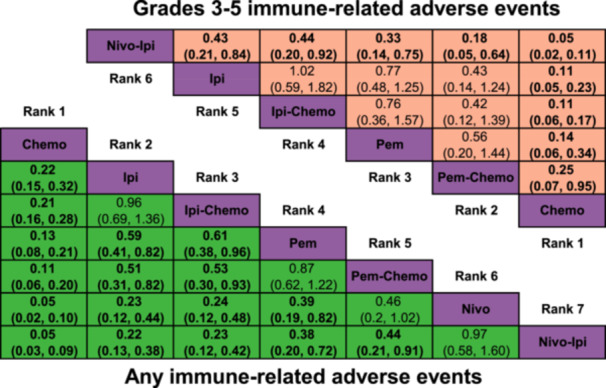
Odds ratio (95% CrI) estimated from Bayesian network meta‐analysis of any and grades 3–5 immune‐related adverse events associated with each treatment regimen. Chemo, chemotherapy; Ipi, ipilimumab; Nivo, nivolumab; Pem, pembrolizumab.

Network plots of any grade and grade 3–5 irAEs are presented in Figure 1B. The ORs and 95% CrIs among the comparisons are shown in Figure 4. The rank probability for each ICI is illustrated in Figure 3. In terms of any grade and grade 3–5 irAEs, Chemo was linked to the lowest risk and offered a higher safety margin than most ICIs, with ORs ranging from 0.03 to 0.95. Chemo was ranked as the safest, whereas Nivo‐Ipi was among the least safe treatments. Furthermore, Chemo, Ipi, and Ipi‐Chemo were significantly associated with the lowest risk of any irAEs as compared to treatment with other ICIs as monotherapies or in combination (ORs ranging between 0.12 and 0.96). However, we found no significant difference among most of the grade 3–5 irAEs, except when compared with Nivo‐Ipi or Chemo.

#### 3.2.2 Secondary Outcomes

The likelihood of an ICI‐treatment regimen being ranked as least risky depends on the SOC, in addition to irAEs and trAEs. In this study, we assessed trAEs in 11 SOCs and irAEs in four SOCs. Network plots are shown in Figures S2 and S3. The ORs and 95% Crls for the comparisons are shown in Figures S4 and S5. The rank probabilities for each ICI regimen are shown in Figures S6 and S7.

Initially, we assessed the trAEs across various SOCs. Tremelimumab (Treme), Ipi, and Pem were identified as being related to a minimal risk of disorders of the blood and lymphatic systems, with ORs varying from 0.00002 to 0.89. In contrast, Sparta‐Chemo, Chemo, Ipi‐Chemo, and Ipi were the least safe in this regard. For endocrine disorders, Chemo was associated with the lowest risk, with ORs varying from 0.001 to 0.90, while Pem‐Chemo and Pem were identified as the regimens with the lowest safety. For gastrointestinal disorders, Nivo and Pem were associated with the lowest risk, with ORs ranging from 0.001 to 0.76, atezolizumab plus chemotherapy (Atezo‐Chemo) one of the most unsafe ICI regimens. For general disorders and administration site conditions, Ipi, Nivo‐Ipi‐Chemo, and Nivo were the treatments with the lowest risk rankings, with ORs ranging from 0.001 to 0.98, while Pem‐Chemo and Atezo‐Chemo were among the treatments considered the least safe. For investigations, Pem and Ipi were the ICIs deemed to have the lowest risk, with ORs between 0.04 and 0.99, while Ipi‐Chemo was associated with the highest risk. Among the ICIs evaluated for metabolic and nutritional disorders, Ipi, Nivo, and Pem had the lowest risks, with ORs between 0.09 and 0.85. Conversely, Treme was identified as the ICI with the lowest safety level in this regard. Ipi presented the lowest risk of musculoskeletal and connective tissue disorders, with ORs ranging from 0.12 to 0.98, while Pem‐Chemo presented the highest risk. Pem and Ipi had the lowest risk of causing nervous system disorders, with ORs from 0.02 to 0.92, whereas Atezo was one of the least safe ICIs in this context. The Ipi and Ipi‐Chemo groups had the lowest risk of respiratory, thoracic, and mediastinal disorders, with ORs between 0.02 and 0.89, while Sparta‐Chemo had the highest risk. Chemo and Sparta‐Chemo had the lowest risk of skin and subcutaneous tissue disorders, with ORs ranging from 0.005 to 0.89, while Atezo‐Chemo was the least safe treatment option.

Second, we assessed irAEs across various SOCs. Chemo, Ipi‐Chemo, and Ipi were associated with a low incidence of endocrine disorders, with ORs ranging from 0.01 to 0.84, while nivolumab plus relatlimab (Nivo‐Relat) demonstrated the least safety. Chemo was the safest option for gastrointestinal disorders, although no significant differences were observed among most comparisons. However, Nivo‐Relat was identified as the least safe option in this regard. Ipi was the safest in terms of respiratory, thoracic, and mediastinal system disorder, with ORs ranging between 0.01 and 0.79, while Nivo‐Relat was the least safe treatment option. Chemo was associated with the lowest risk of skin and subcutaneous tissue disorders, with ORs ranging between 0.03 and 0.24, whereas Nivo‐Ipi had the lowest safety rating.

#### 3.2.3 Heterogeneity and Inconsistency

The included trials exhibited positive transitivity and consistency, thereby enabling both direct and indirect comparisons. According to the *Q* test and *I* ^2^ statistic, the majority of heterogeneity and inconsistency across the included studies were either minimal (*I* ^2^ = 0%) or low (*I* ^2^ < 25%) (Figure S8).

### 3.3 Pharmacovigilance Analysis

#### 3.3.1 AEs Among Melanoma Patients Treated With ICIs in the FAERS

The FAERS database included 22,706 cases of ICI treatment in patients with melanoma (Table S3). Data on AEs in melanoma patients who were treated with ICIs were collected, while cases involving concurrent medications or related treatment indications were excluded. The number of yearly reported cases remained relatively steady. The experience of AEs among patients with melanoma varied according to the ICI‐treatment strategy used. Given the percentages of AEs observed with different ICI approaches, the data indicated that ICIs may be responsible for a substantial number of these AEs.

#### 3.3.2 Scanning for ICI‐Related AEs

**Figure 5:**
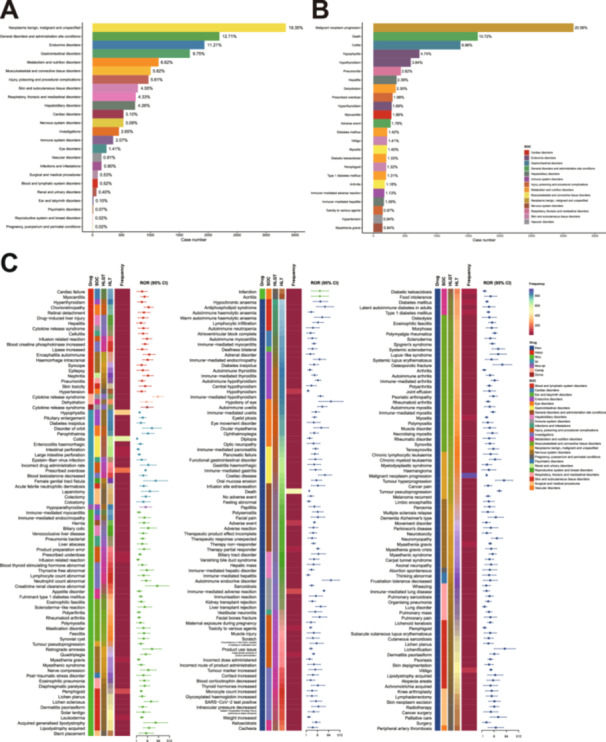
Scanning for ICI‐related adverse event signals based on the FAERS database. (A) Bar plot showing the distribution of significant PT signals across SOCs. The percentage values labeled in the figure represent the proportion of cases with the corresponding adverse events among all ICI‐related AE cases. (B) Bar plot showing the top PT signals of adverse events. The color indicates the SOC of the corresponding PT. The percentage values labeled in the figure represent the proportion of cases with such adverse events out of the total ICI‐related adverse event cases. (C) Heatmap and forest plot showing the RORs for 236 PT signals with at least three cases and a lower limit of the 95% CI greater than or equal to 1 in the FAERS database under different ICI treatment strategies, including Pem, Nivo, Atezo, Ipi, Cemip, Durva, and Nivo‐Ipi. The color indicates the ICI regimen, SOC, HLGT, HLT, and PT frequency. This figure also depicts the hierarchical relationship of PTs for categories of ICI‐related AEs in MedDRA. Due to the limitation of the figure, the legends of HLT and HLGT were provided in Figure S9. Atezo, atezolizumab; Cemip, cemiplimab; CI, confidence interval; Durva, durvalumab; FAERS, FDA adverse event reporting system; HLGT, high level group term; HLT, high level term; ICI, immune checkpoint inhibitors; Ipi, ipilimumab; Nivo, nivolumab; Pem, pembrolizumab; PT, preferred term; ROR, reporting odds ratio; SOC, systemic organ class.

We tabulated the categories and quantities of AEs obtained from the FAERS reports of patients with melanoma treated with ICIs (Table S4). Malignant neoplasm progression (*N* = 3251, 14.34%), diarrhea (*N* = 1750, 7.72%), death (*N* = 1666, 7.35%), colitis (*N* = 1410, 6.22%), and fatigue (*N* = 1213, 5.35%) were the five highest categories of AEs. We computed the RORs for PTs that had a minimum of three instances of AEs in comparison with the complete set of AEs for patients with melanoma in the FAERS database. Patients with melanoma who met the aforementioned criteria were categorized as having ICI‐related AEs. Reports involving 5974 AEs associated with ICIs in 4599 patients with melanoma were examined. We found that seven ICIs, either as monotherapy or in combination, were associated with a significant signal in 236 PTs belonging to 24 distinct SOCs (Figure 5 and Figure S9). Among ICI‐related AEs, the most frequently reported AEs were benign, malignant, and unspecified neoplasms, general disorders and administration site conditions, and endocrine disorders, categorized by different SOCs (Figure 5A). However, immune system disorders constituted only 2.07% of these reports. Moreover, the progression of malignant neoplasms (20.56%), death (10.72%), and colitis (8.96%) were identified as the top‐three AEs associated with ICIs (Figure 5B). Figure 5C shows a comparison of the RORs for various ICI treatments. Disproportionality analysis revealed 166 significant signals for Pem, 45 for Nivo, 20 for Atezo, 18 for Ipi, two for cemiplimab (Cemip), 1 for durvalumab (Durva), and 1 for Nivo‐Ipi within the PTs. A total of 236 PTs were associated with 24 SOCs. Following the disproportionality analysis of the PTs associated with SOCs, we identified 22 significant safety signals for Pem, 16 for Nivo, 13 for Atezo, 9 for Ipi, 2 for Cemip, 1 for Durva, and 1 for Nivo‐Ipi. Upon closer examination of the safety signals identified for PTs, the most commonly reported AEs were as follows: for Pem, the progression of malignant neoplasms (*N* = 942), death (*N* = 493), and immune‐mediated adverse reactions (*N* = 151); for Nivo, pemphigoid (*N* = 127); and for Atezo, an increase in blood creatine phosphokinase levels (*N* = 11).

#### 3.3.3 Descriptive Analysis of Fatal and Non‐Fatal Cases

Following the search for ICI reports in the FAERS database (*N* = 4599) (Table 1), we conducted a statistical analysis of the clinical characteristics of ICI‐related AEs. The median age of the included patients was 65 years (interquartile range, 54–74 years). A significant number of ICI‐related AE cases was reported in males (*N* = 2457, accounting for 60%), and the majority of cases were reported in the United States (*N* = 2084, representing 45% of the total). Fatal outcomes were associated with 25.35% of cases (*N* = 1166).

**Table 1:** Characteristics of reports with ICI‐related adverse events of melanoma patients sourced from the FAERS database.

Clinical characteristics	Overall (*N* = 4599)	Fatal (*N* = 1166)	Non‐fatal (*N* = 3433)	*p*‐value
Gender				0.3
Male	2457 (60%)	681 (62%)	1776 (60%)	
Female	1616 (40%)	424 (38%)	1192 (40%)	
Missing	526	61	465	
Age				
Median (IQR)	65 (54, 74)	66 (54, 75)	65 (55, 74)	0.8
Missing	1266	256	1010	
Age group				0.06
18–64	1619 (49%)	442 (49%)	1177 (49%)	
65–75	912 (27%)	227 (25%)	685 (28%)	
≥ 75	802 (24%)	241 (26%)	561 (23%)	
Missing	1266	256	1010	
Country				< 0.001
United States of America	2084 (45%)	270 (23%)	1814 (53%)	
France	370 (8.0%)	59 (5.1%)	311 (9.1%)	
Japan	296 (6.4%)	72 (6.2%)	224 (6.5%)	
Germany	220 (4.8%)	54 (4.6%)	166 (4.8%)	
Australia	191 (4.2%)	49 (4.2%)	142 (4.1%)	
Canada	153 (3.3%)	73 (6.3%)	80 (2.3%)	
United Kingdom of Great Britain and Northern Ireland	145 (3.2%)	37 (3.2%)	108 (3.1%)	
Italy	106 (2.3%)	43 (3.7%)	63 (1.8%)	
Other country	1034 (22%)	509 (44%)	525 (15%)	
Received year				< 0.001
2012	57 (1.2%)	12 (1.0%)	45 (1.3%)	
2013	105 (2.3%)	21 (1.8%)	84 (2.4%)	
2014	162 (3.5%)	49 (4.2%)	113 (3.3%)	
2015	545 (12%)	276 (24%)	269 (7.8%)	
2016	518 (11%)	162 (14%)	356 (10%)	
2017	660 (14%)	94 (8.1%)	566 (16%)	
2018	645 (14%)	96 (8.2%)	549 (16%)	
2019	562 (12%)	54 (4.6%)	508 (15%)	
2020	327 (7.1%)	40 (3.4%)	287 (8.4%)	
2021	341 (7.4%)	59 (5.1%)	282 (8.2%)	
2022	580 (13%)	293 (25%)	287 (8.4%)	
2023	97 (2.1%)	10 (0.9%)	87 (2.5%)	
Case priority				< 0.001
Direct	80 (1.7%)	13 (1.1%)	67 (2.0%)	
Expedited	3919 (85%)	1145 (98%)	2774 (81%)	
Non‐expedited	600 (13%)	8 (0.7%)	592 (17%)	
Reporter type				< 0.001
Healthcare professional	3143 (69%)	694 (60%)	2449 (72%)	
Consumer	1438 (31%)	468 (40%)	970 (28%)	
Lawyer	3 (< 0.1%)	0 (0%)	3 (< 0.1%)	
Missing	15	4	11	
Treatment strategy				< 0.001
Pem	2615 (57%)	914 (78%)	1701 (50%)	
Ipi	1238 (27%)	164 (14%)	1074 (31%)	
Nivo	646 (14%)	85 (7.3%)	561 (16%)	
Atezo	84 (1.8%)	3 (0.3%)	81 (2.4%)	
Cemip	7 (0.2%)	0 (0%)	7 (0.2%)	
Nivo‐Ipi	6 (0.1%)	0 (0%)	6 (0.2%)	
Durva	3 (< 0.1%)	0 (0%)	3 (< 0.1%)	

Abbreviations: Atezo, atezolizumab; Durva, durvalumab; FAERS, FDA adverse event reporting system; ICI, immune checkpoint inhibitors; Ipi, ipilimumab; Nivo, nivolumab; Pem: pembrolizumab.

A comparative analysis of fatal and non‐fatal groups regarding the utilization of different types of ICIs could offer insights into how to mitigate fatal outcomes in patients with melanoma. Utilization of different types of ICIs, alone or in combination, was significantly different between the fatal and nonfatal groups (*p* < 0.001). Pem accounted for a higher proportion of fatal reports. However, sex and age did not differ between the fatal and non‐fatal groups (*p* = 0.3, *p* = 0.8, and *p* = 0.06, respectively).

## 4 Discussion

The increasing use of ICIs has led to an increase in AE reports, which requires further investigation. This study provided a comprehensive analysis of AEs associated with ICI‐treatment in patients with melanoma, utilizing data from both RCTs and real‐world cases from the FAERS, which has not been reported previously. In total, 11,937 patients from 31 RCTs on melanoma were included in the NMA. By conducting a disproportionality analysis of all AE cases reported in patients with melanoma in the FAERS database, we identified AEs that strongly correlated with the types of ICI used and assessed the characteristics of patients with melanoma associated with these AEs.

This NMA provided several significant insights into the risk of AEs among patients with melanoma in relation to various ICI regimens. First, Ipi, Nivo, and Pem were associated with the lowest risk of any grade or grade 3–5 trAEs, whereas Sparta‐Chemo was among the least safe treatments in terms of trAEs. Second, Chemo was linked to the lowest risk across all grades, and specifically grades 3–5 irAEs, whereas Nivo‐Ipi was the least safe among the ICIs for irAEs. Moreover, compared with other treatments, Ipi and Ipi‐Chemo were associated with the lowest risk of irAEs. Third, upon comparing different ICI regimens in relation to AEs across various SOCs, the ICI types were ranked according to their risk of trAEs or irAEs. For trAEs, Treme, Ipi, and Pem were related to a reduced risk of blood and lymphatic system disorders; Nivo and Pem were linked to a decreased risk of gastrointestinal disorders; Ipi was associated with a diminished risk of general disorders and administration site conditions, as well as musculoskeletal and connective tissue disorders; Pem and Ipi were tied to a lower risk of investigations; Ipi, Nivo, and Pem were associated with a reduced risk of metabolism and nutrition disorders; Pem and Ipi were linked to a lower risk of nervous system disorders; and Ipi and Ipi‐Chemo were associated with a lower risk of respiratory, thoracic, and mediastinal disorders. However, compared with traditional Chemo regimens, ICIs were associated with a higher risk of endocrine disorders. In terms of irAEs, Ipi was associated with the lowest risk of disorders of the respiratory, thoracic, and mediastinal areas. However, compared to Chemo, ICIs were associated with a higher risk of endocrine, gastrointestinal, skin, and subcutaneous tissue disorders.

Valuable insights were obtained from disproportionality analysis. The FAERS pharmacovigilance data analysis revealed that the FAERS database contained 22,706 reports pertaining to ICI immunotherapy in patients with melanoma. In addition, 5974 AEs related to ICI were identified and were analyzed in 4599 patients with melanoma. Disproportionality analysis indicated 166 significant safety signals for Pem. For Pem, apart from malignant neoplasm progression and death, the most common PT signal was an immune‐mediated adverse reaction. For Nivo, the most common PT signal was pemphigoid.

As previously reported, Ipi, Nivo, and Pem were the safest in terms of grade 3–5 trAEs, while Chemo exhibited the most favorable safety profile for irAEs of grades 3–5 [4]. A previous study reported that Pem (at 2 mg/kg or 10 mg/kg, every 3 weeks), and Nivo (3 mg/kg, every 2 weeks) may be the preferred treatment regimens (with respect to irAE risks) among the ICI regimens reported [2]. Our study showed similar results. However, another study indicated a contrasting result, finding no significant difference in AEs between Nivo‐Ipi and Ipi [49]. This study differed in several ways from previously published studies. For example, earlier studies only provided limited evidence on irAE results and did not provide evidence for new ICIs, such as Atezo or Sparta, and did not include as many RCTs [2, 4, 49]. In this study, we designated the rates of trAEs and irAEs as potential efficacy endpoints of ICI therapy, even though this was usually considered a safety endpoint in the included RCTs. We incorporated recently published RCTs that assessed new ICIs, such as Atezo and Sparta, and ranked all the therapeutic strategies evaluated in the RCTs. Our approach differed from that used in previous studies, in which melanoma therapeutic strategies were ranked differently from those for other cancers. Additionally, we obtained real‐world data from the FAERS database and performed a pharmacovigilance analysis.

Thus, this was an inaugural study for scrutinizing trAEs and irAEs linked to various ICI protocols for melanoma, amalgamating an NMA and real‐world AE cases from the FAERS database, in conjunction with a pharmacovigilance analysis. The safety characteristics of ICIs used to treat melanoma were thoroughly examined in this comprehensive study. Trial‐based and real‐world analyses were integrated to provide a more comprehensive evaluation of ICI‐related AEs in melanoma. Although RCTs offer high‐quality evidence on treatment efficacy and safety under controlled conditions, they are often limited by sample size, follow‐up duration, and selective reporting of AEs. In contrast, the FAERS pharmacovigilance database captures spontaneous reports from a broader patient population, thereby complementing RCT findings by identifying rare, delayed, or unexpected AEs that may not be observed in clinical trials. In this study, the FAERS data were used to complement the safety signals observed in RCTs and to detect additional signals across diverse organ systems, providing complementary evidence that strengthens the robustness and clinical relevance of our conclusions.

Mechanistically, the more favorable tolerability of PD‑1 inhibitors as compared to CTLA‑4 inhibitors may be explained by differences in their immunological targets. PD‑1 blockade primarily modulates peripheral T‑cell activity within the tumor microenvironment, whereas CTLA‑4 inhibition exerts broader effects on early T‑cell activation, leading to more widespread immune dysregulation. This distinction likely contributes to the lower incidence of severe immune‐related toxicities observed with PD‑1 inhibitors. These mechanistic insights may help explain the observed differences in tolerability and may support individualized toxicity monitoring rather than definitive regimen selection [50]. Recent reviews have emphasized that the tumor microenvironment plays a pivotal role in shaping ICI responsiveness and resistance, as immune evasion mechanisms in melanoma can limit treatment efficacy, highlighting the need for predictive biomarkers to optimize patient outcomes [51]. Accordingly, PD‑1 inhibitors showed comparatively favorable tolerability signals in this study, which may help inform individualized safety assessment and toxicity monitoring; however, treatment selection should also consider efficacy, patient characteristics, guideline recommendations, and clinical context. Oncologists, emergency department physicians, critical care providers, and other experts must be aware of the grave toxic effects of ICIs utilized in various types of cancer. The most frequent manifestation of AEs in diverse organ systems is immune‐mediated damage to normal tissues. Variations in the risk of AEs could plausibly be connected to the distinct mechanisms of action of each ICI [52]. Additional clinical studies are required to evaluate and confirm the potential relationship between ICIs and these AEs. Moreover, given the significant risk of irAEs, treatment with ICIs should be performed with particular caution. Previously published guidelines on the management of irAEs have suggested conducting a thorough clinical examination to establish baselines before starting ICI therapy [53, 54]. High‐occurrence AEs should be monitored closely once immunotherapy has been selected. The clinical determination of ICI therapy should be based on the findings presented in this study. Clinicians should ascertain the duration and type of immunosuppressive therapy according to the severity of irAEs and should contemplate the possibility of reintroducing ICIs after their discontinuation. This method emphasizes the importance of diagnosing and treating patients on a case‐by‐case basis [55].

These findings have potential clinical implications for regimen selection and AE monitoring in patients with melanoma receiving ICI treatment. Given our identification of regimens such as Ipi, Nivo, and Pem as being associated with lower risks of severe trAEs, these agents may represent regimens with comparatively favorable trAE safety signals when treatment efficacy and tolerability are considered together. Conversely, regimens linked to higher risks of irAEs, including Nivo‐Ipi and certain Chemo–immunotherapy combinations, may warrant closer AE monitoring. The organ‐specific AE profiles derived from both the RCT and FAERS pharmacovigilance data may provide useful information for tailoring surveillance strategies; for example, endocrine and gastrointestinal toxicities should be closely monitored in patients receiving certain ICIs, whereas musculoskeletal and respiratory events may be more relevant for other regimens. However, these findings should be interpreted as comparative safety signals rather than direct prescribing recommendations, and clinical decisions should not be based solely on these results.

## 5 Limitations

This study had some limitations. Initially, the NMA results exhibited a broad spectrum of CrIs. This can be attributed to the limited number of studies, small sample sizes of some of the included trials, and varying standards of reporting. Second, our ability to rank the types of ICIs according to their likelihood of being associated with the lowest risk for each AE was constrained. Due to the limited number of trials available, we were unable to evaluate and rank several treatment options and SOCs. Third, the FAERS database serves as a global system for spontaneous incident reporting. Numerous factors contribute to data‐selection bias, including ethnicity, geographical location, and comprehension of AEs. These limitations prevented us from calculating the risk of ICI‐related AEs and establishing a causal link between ICIs and AEs. Furthermore, we could not extract more detailed characteristics of AEs or the precise time of death in patients with melanoma. We were also unable to perform additional competitive risk analyses of ICIs and AEs. In addition, the FAERS data are subject to underreporting and duplicate submissions, and the absence of exposure denominators precludes the estimation of true incidence rates. Finally, AE cases have only been documented for a handful of ICIs in the FAERS database, with no reports of AE cases for new ICIs. Accordingly, our FAERS analysis could not provide such evidence.

## 6 Conclusions

In summary, an extensive investigation into AEs associated with various types of ICIs was conducted using data from RCTs and the FAERS database, employing NMA and disproportionality analyses. Based on our findings, Ipi, Nivo, and Pem may be the preferred ICI regimens for melanoma, considering that they are associated with comparatively low risk of any grade or grade 3–5 trAEs. Chemo was associated with the lowest risk of any grade and grade 3–5 irAEs. Sparta‐Chemo and Nivo‐Ipi should be administered with caution and close monitoring, because of their higher risk of any grade or grade 3–5 trAEs and irAEs, respectively. While the AE spectra of the ICIs varied, Pem exhibited significant signals, and apart from malignant neoplasm progression and death, immune‐mediated adverse reactions were the most frequently encountered PT signals. These findings should be interpreted as comparative safety signals rather than definitive prescribing guidance, and treatment decisions should continue to consider efficacy, patient characteristics, guideline recommendations, and clinical context.

## Author Contributions

Yiwei Chen, Xueyan Liang, and Yan Li designed the study, collected and managed the data. Yiwei Chen and Xueyan Liang drafted the manuscript. Yan Li and Xiaoyu Chen critically reviewed the manuscript. Xueyan Liang and Xiaoyu Chen supervised the study. Yiwei Chen and Yan Li obtained funding. All authors have read and approved the final version of the manuscript. Yan Li had full access to all of the data in this study and takes complete responsibility for the integrity of the data and the accuracy of the data analysis.

## Ethics Statement

The authors have nothing to report.

## Consent

The authors have nothing to report.

## Conflicts of Interest

The authors declare no conflicts of interest.

## Transparency Statement

The corresponding author (Yan Li) affirms that this manuscript is an honest, accurate, and transparent account of the study being reported; that no important aspects of the study have been omitted; and that any discrepancies from the study as planned have been explained.

## Supporting information


Supporting File

## Data Availability

The FAERS are publicly available at https://www.fda.gov/drugs/questions-and-answers-fdas-adverse-event-reporting-system-faers/fda-adverse-event-reporting-system-faers-public-dashboard.
