# Supplementary material for: The Real‐World Safety Profile of Immune Checkpoint Inhibitors in Melanoma: FAERS Pharmacovigilance Analysis Complemented by Randomized Trial Evidence
Source: Health Sci Rep. 2026 Jul 28;9(8):e72865. doi: 10.1002/hsr2.72865 (PMC13412547; doi:10.1002/hsr2.72865)
Supplement: Supplementary file 1 — Supporting File [file HSR2-9-e72865-s001.docx]

**Supplementary Content**

**Supplementary Figure S1**. Preferred reporting items for systematic reviews and meta-analyses (PRISMA) flow diagram.

**Supplementary Figure S2.** Network plots of system organ classes specific treatment-related adverse events of ICIs for Melanoma.

**Supplementary Figure S3.** Network plots of system organ classes specific immune-related adverse events of ICIs for melanoma.

**Supplementary Figure S4**. Odds ratio (95% CrI) of system organ classes specific treatment-related adverse events associated with each treatment regimen.

**Supplementary Figure S5**. Odds ratio (95% CrI) of system organ classes specific immune-related adverse events associated with each treatment regimen.

**Supplementary Figure S6.** Ranking of the probability of being the best treatment regimen in system organ classes specific treatment-related adverse events.

**Supplementary Figure S7.** Ranking of the probability of being the best treatment regimen in system organ classes specific immune-related adverse events.

**Supplementary Figure S8.** Heterogeneity and inconsistency analysis of network meta-analysis results.

**Supplementary Figure S9.** HLGT and HLT legend of **Fig. 5**.

**Supplementary Table S1.** Characteristics of included randomized controlled trials for melanoma.

**Supplementary Table S2.** Risk of bias assessment of the included studies.

**Supplementary Table S3.** Characteristics of reports with ICI-related adverse events in melanoma patients.

**Supplementary Table S4.** The case number of different adverse events in melanoma cases receiving ICI treatment in FAERS database.


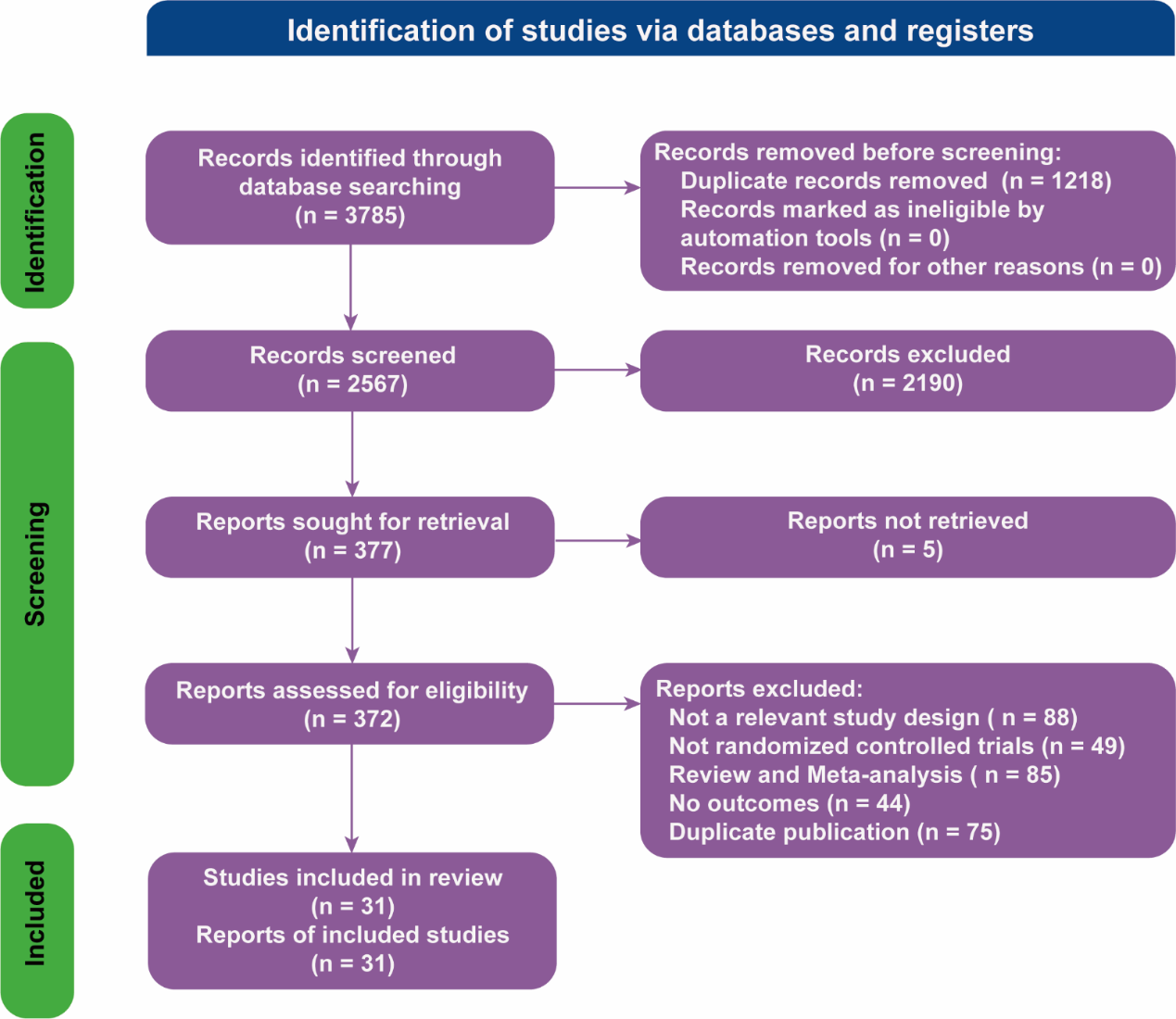


**Supplementary Figure S1**. Preferred reporting items for systematic reviews and meta-analyses (PRISMA) flow diagram.


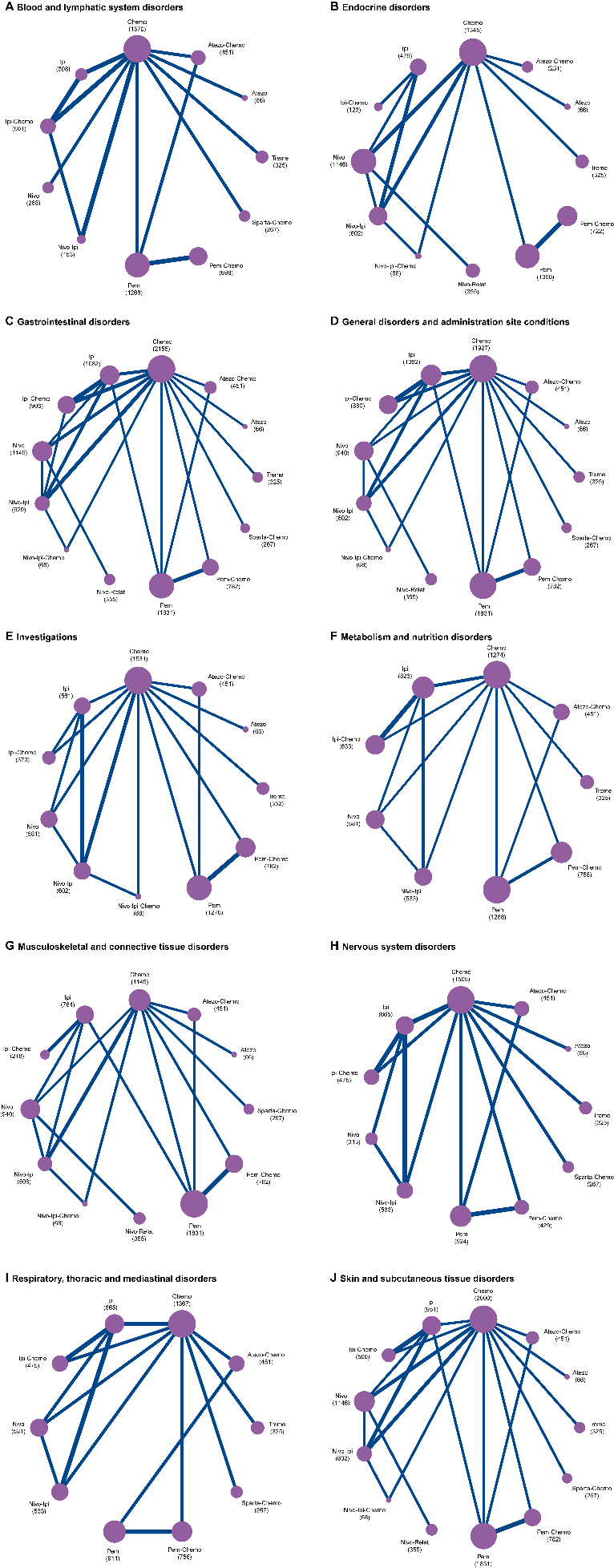


**Supplementary Figure S2.** Network plots of system organ classes specific treatment-related adverse events of ICIs for Melanoma. (A) Blood and lymphatic system disorders; (B) Endocrine disorders; (C) Gastrointestinal disorders; (D) General disorders and administration site conditions; (E) Investigations; (F) Metabolism and nutrition disorders; (G) Musculoskeletal and connective tissue disorders; (H) Nervous system disorders; (I) Respiratory, thoracic and mediastinal disorders; (J) Skin and subcutaneous tissue disorders. Atezo: atezolizumab; Chemo, chemotherapy; Ipi: ipilimumab; Nivo: nivolumab; Pem: pembrolizumab; Relat: relatlimab; Sparta: spartalizumab; Treme: tremelimumab.


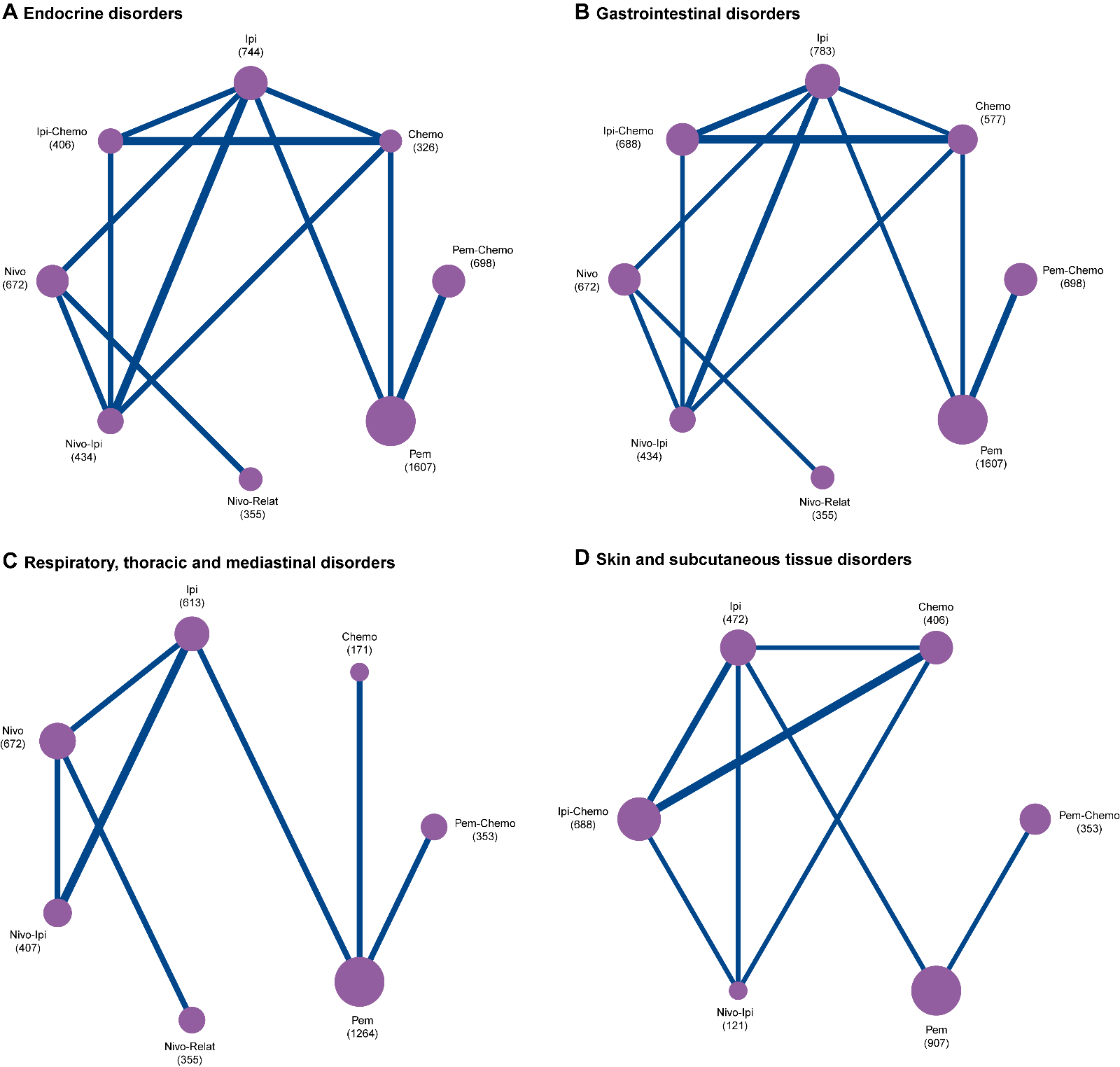


**Supplementary Figure S3.** Network plots of system organ classes specific immune-related adverse events of ICIs for melanoma. (A) Endocrine disorders; (B) Gastrointestinal disorders; (C) Respiratory, thoracic and mediastinal disorders; (D) Skin and subcutaneous tissue disordersChemo, chemotherapy; Ipi: ipilimumab; Nivo: nivolumab; Pem: pembrolizumab; Relat: relatlimab.

**(A) Blood and lymphatic system disorders**


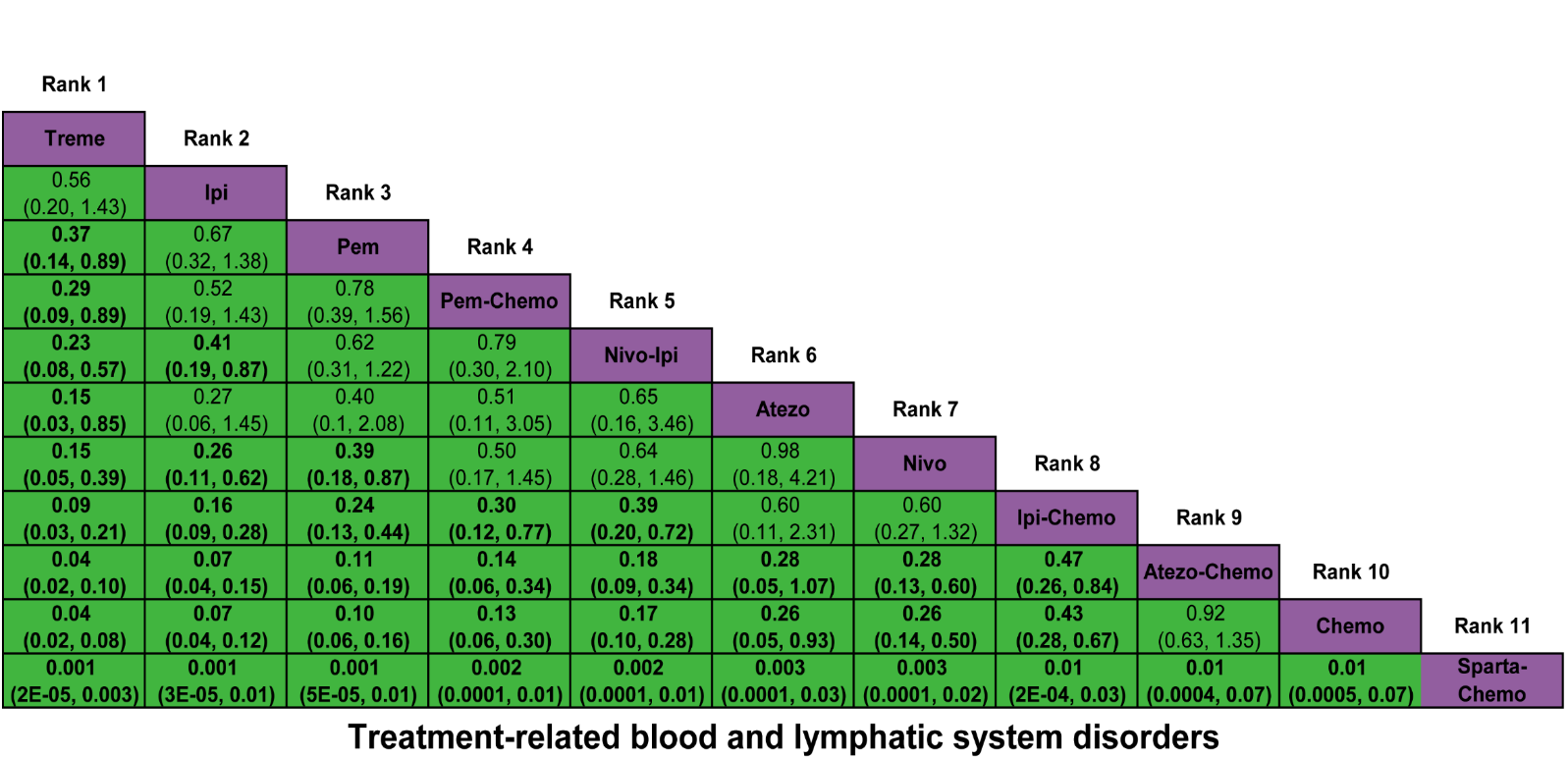


**(B) Endocrine disorders**


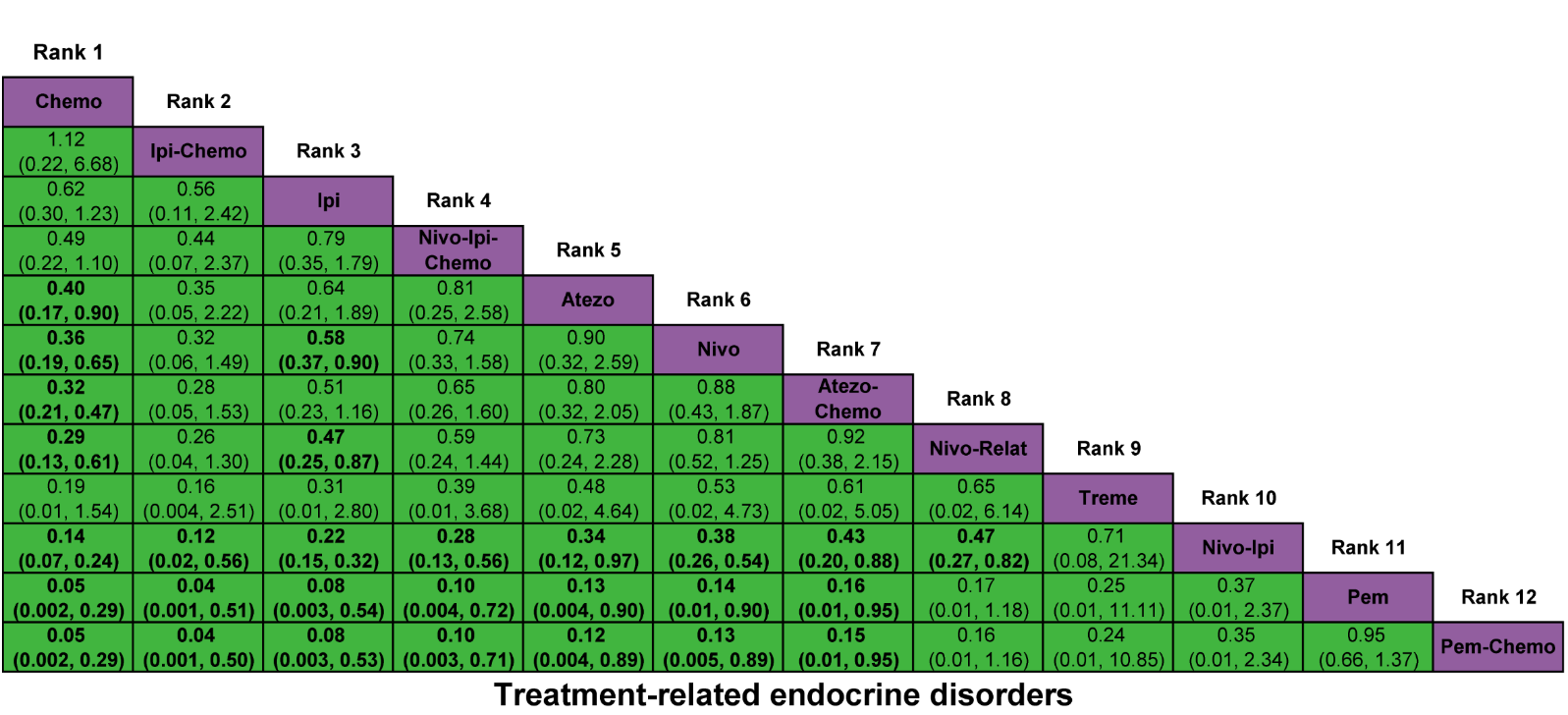


**(C) Gastrointestinal disorders**


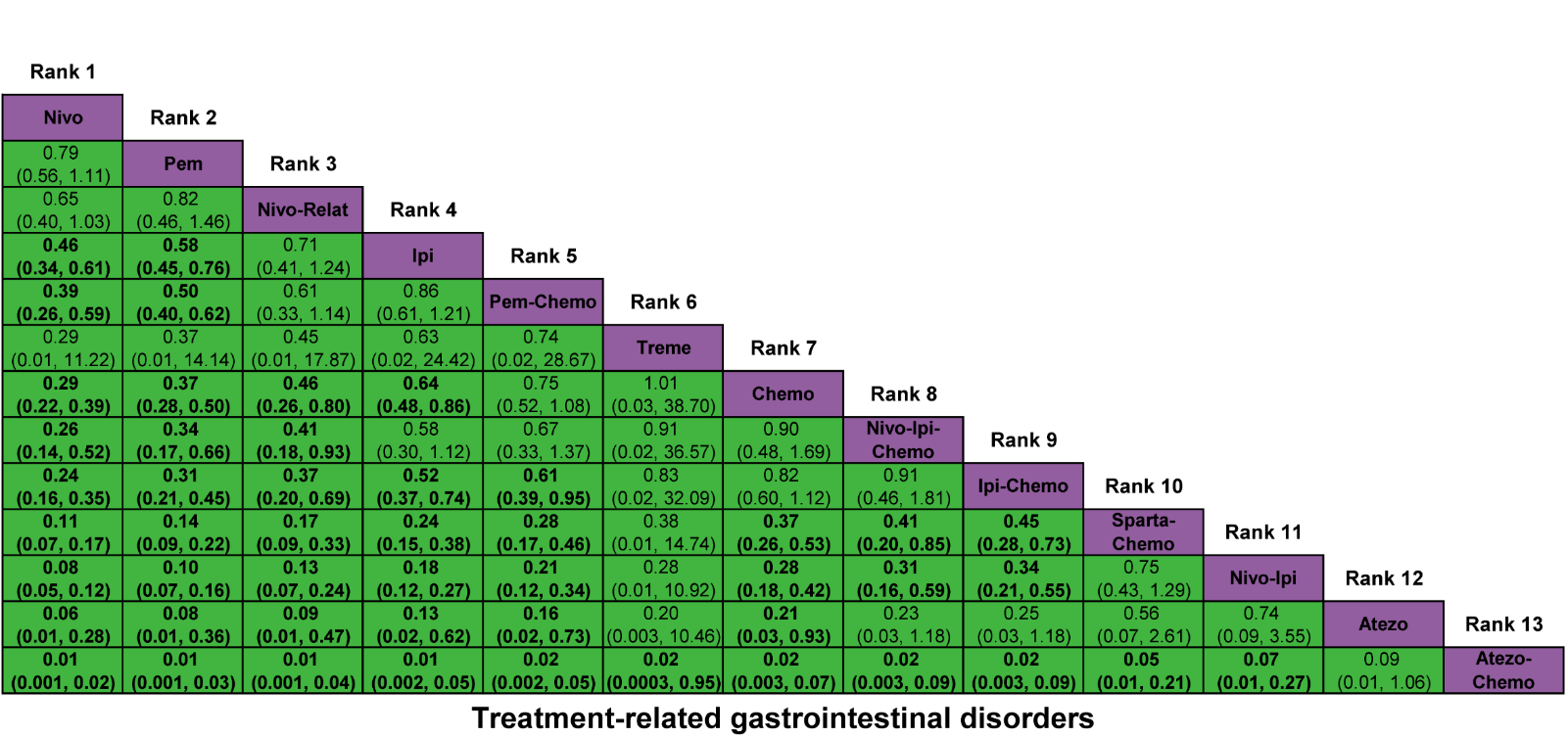


**(D) General disorders and administration site conditions**


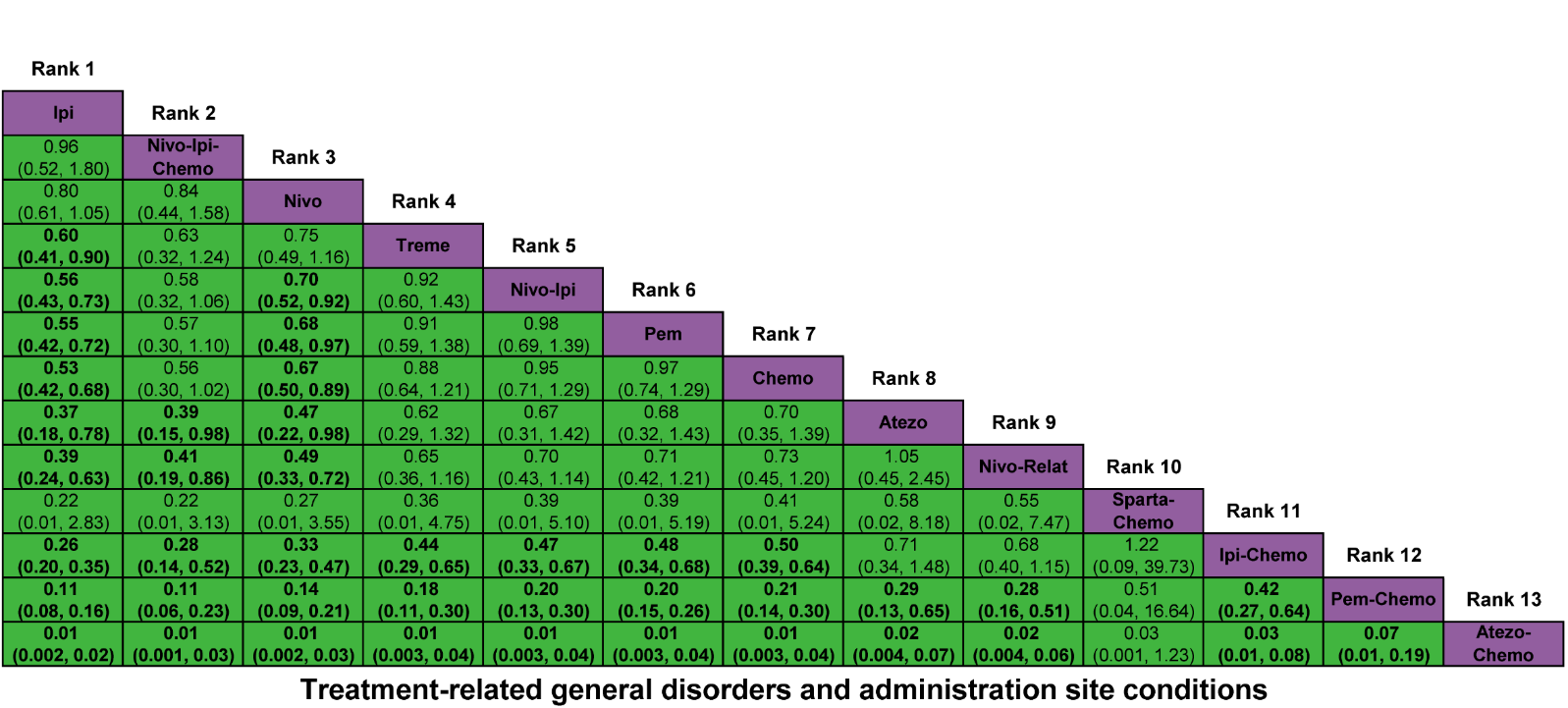


**(E) Investigations**


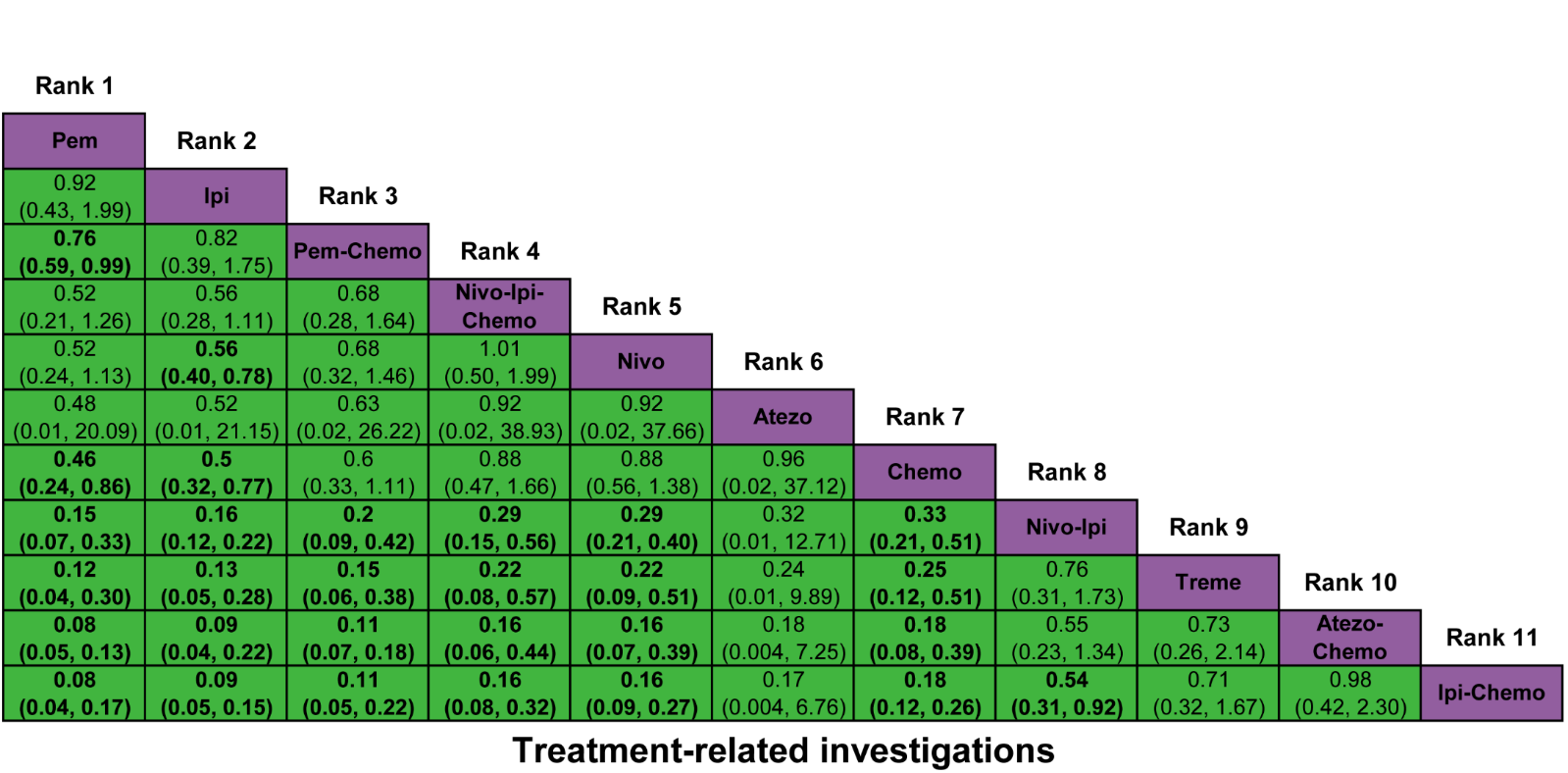


**(F) Metabolism and nutrition disorders**


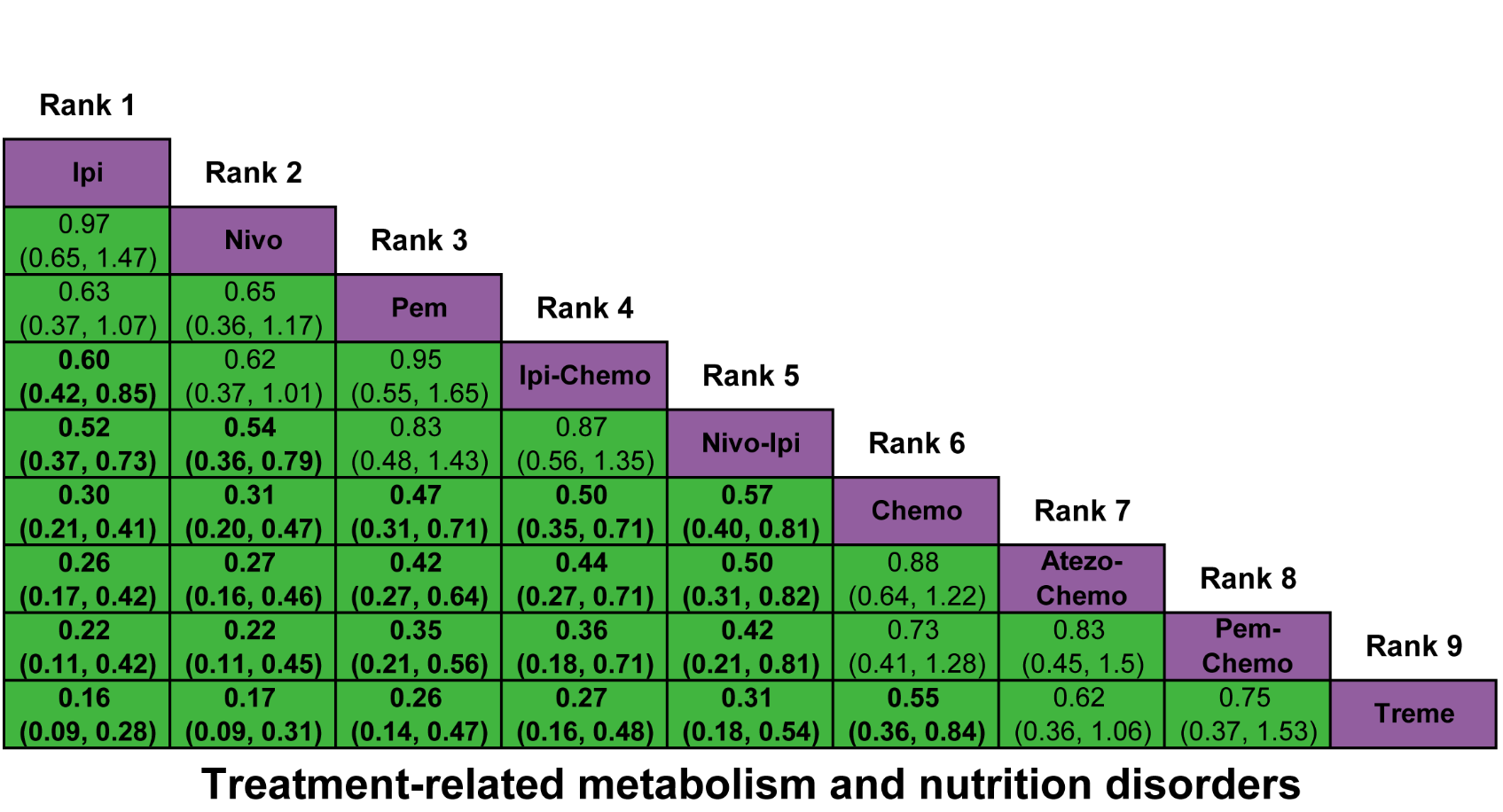


**(G) Musculoskeletal and connective tissue disorders**


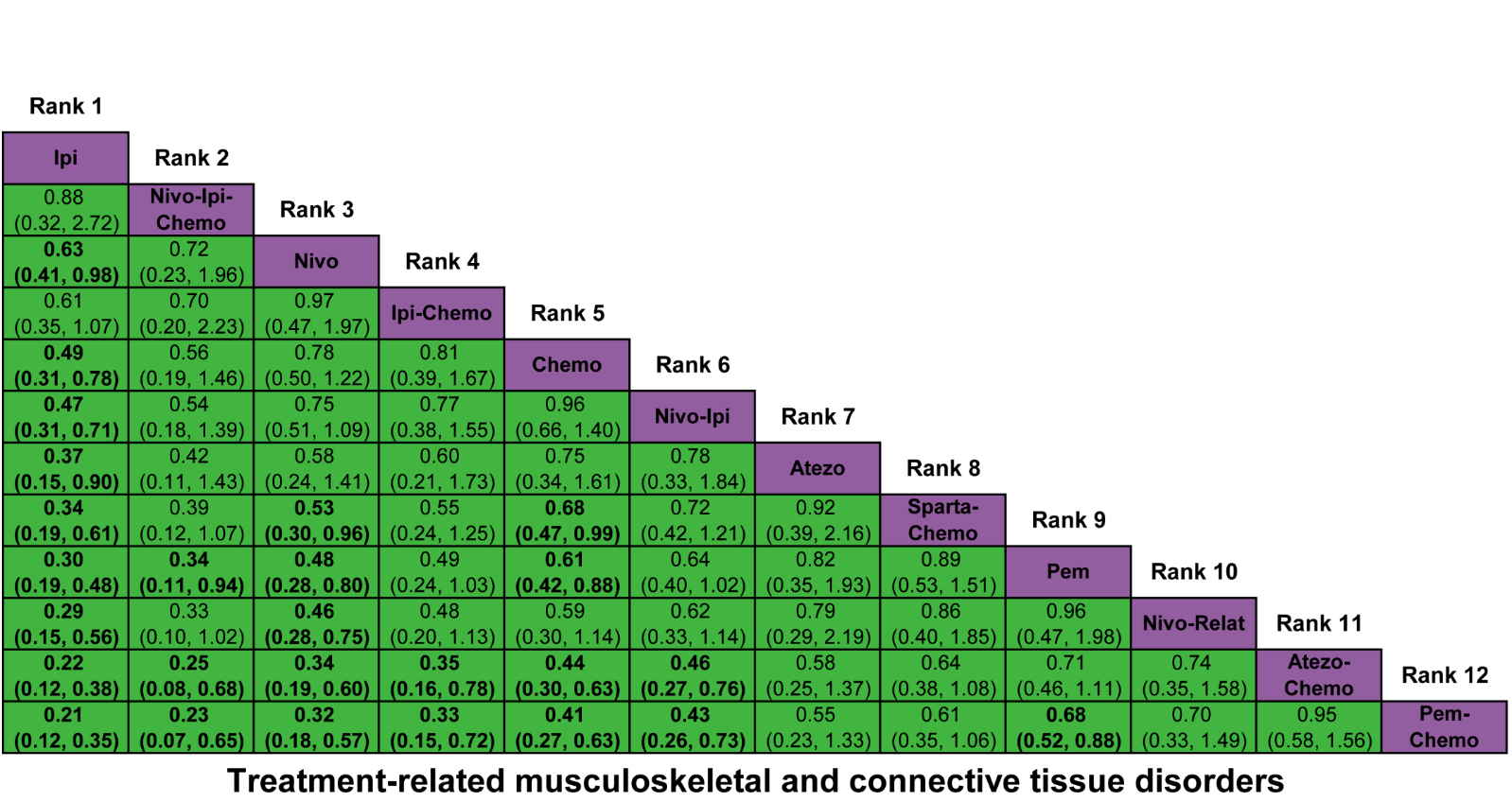


**(H) Nervous system disorders**


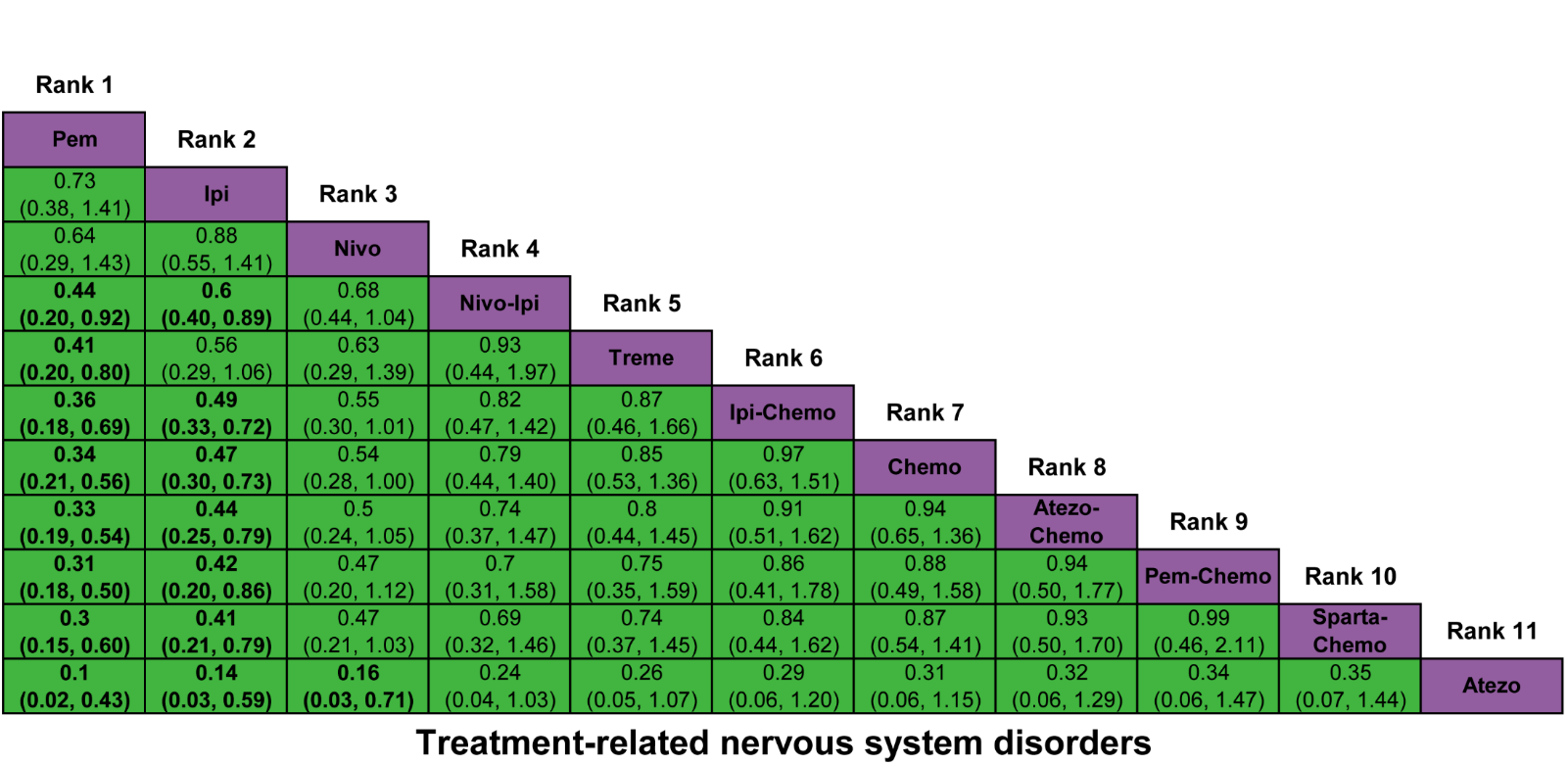


**(I) Respiratory, thoracic and mediastinal disorders**


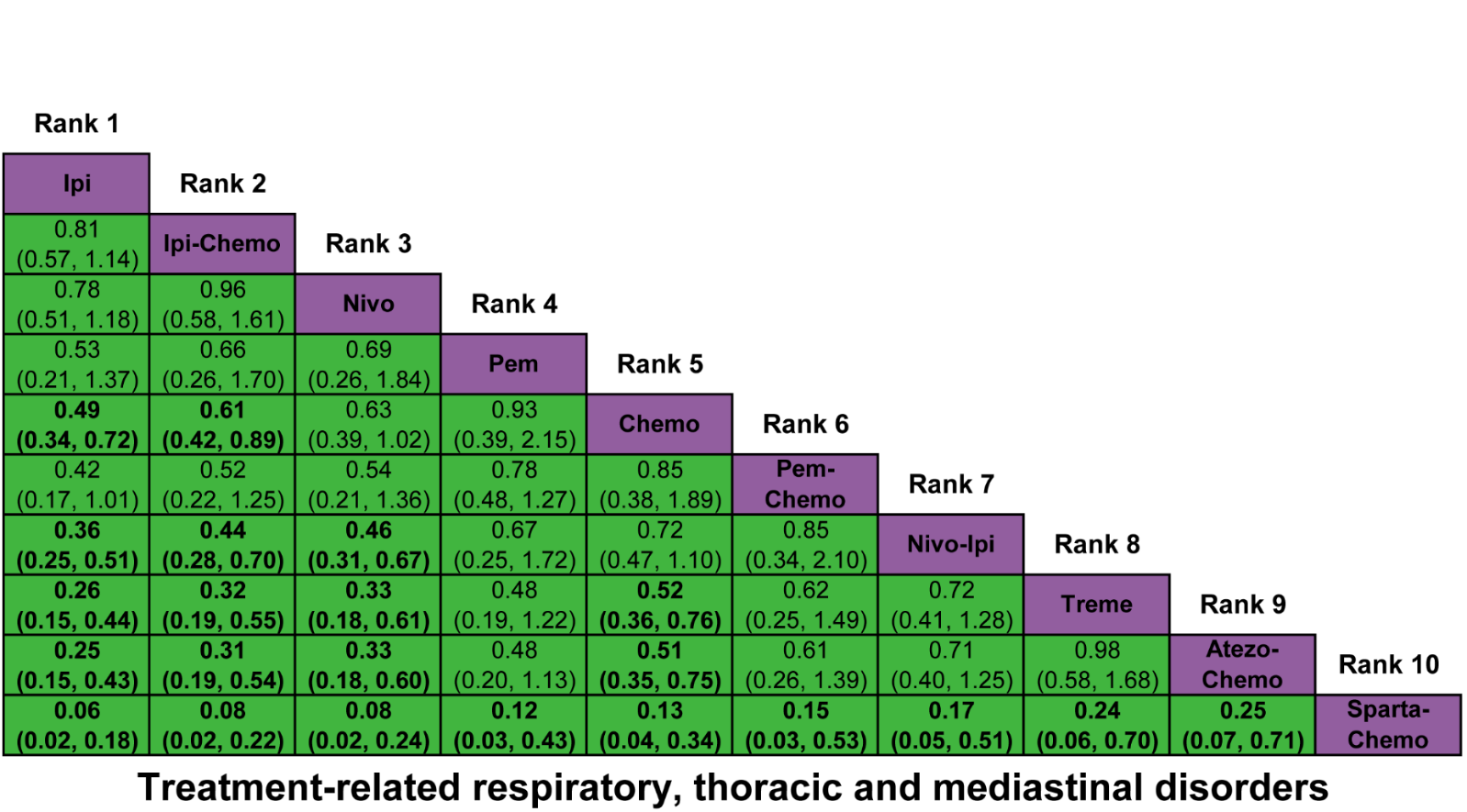


**(J) Skin and subcutaneous tissue disorders**


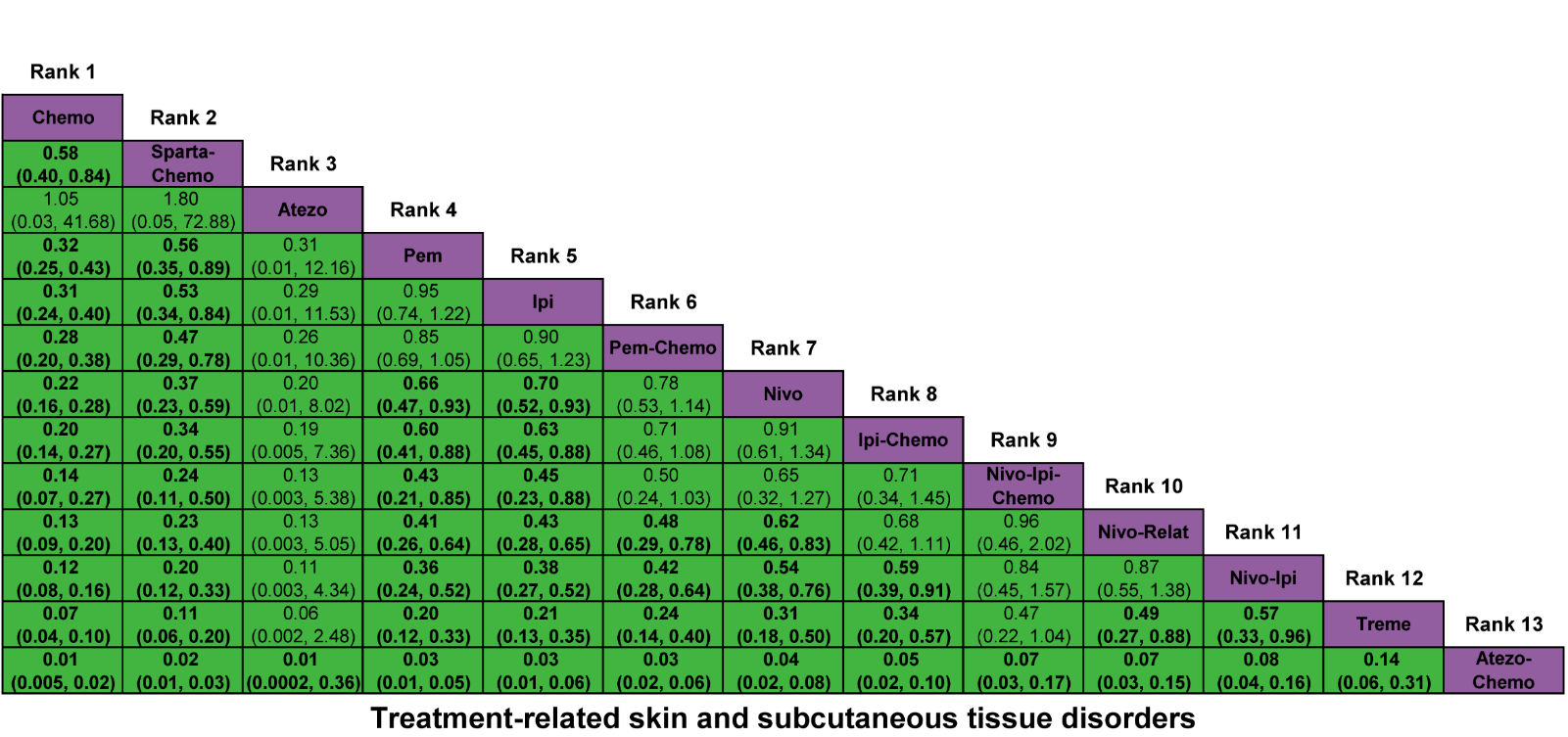


**Supplementary Figure S4**. Odds ratio (95% CrI) of system organ classes specific treatment-related adverse events associated with each treatment regimen. (A) Blood and lymphatic system disorders; (B) Endocrine disorders; (C) Gastrointestinal disorders; (D) General disorders and administration site conditions; (E) Investigations; (F) Metabolism and nutrition disorders; (G) Musculoskeletal and connective tissue disorders; (H) Nervous system disorders; (I) Respiratory, thoracic and mediastinal disorders; (J) Skin and subcutaneous tissue disorders. Atezo: atezolizumab; Chemo, chemotherapy; Ipi: ipilimumab; Nivo: nivolumab; Pem: pembrolizumab; Relat: relatlimab; Sparta: spartalizumab; Treme: tremelimumab.

**(A) Endocrine disorders**


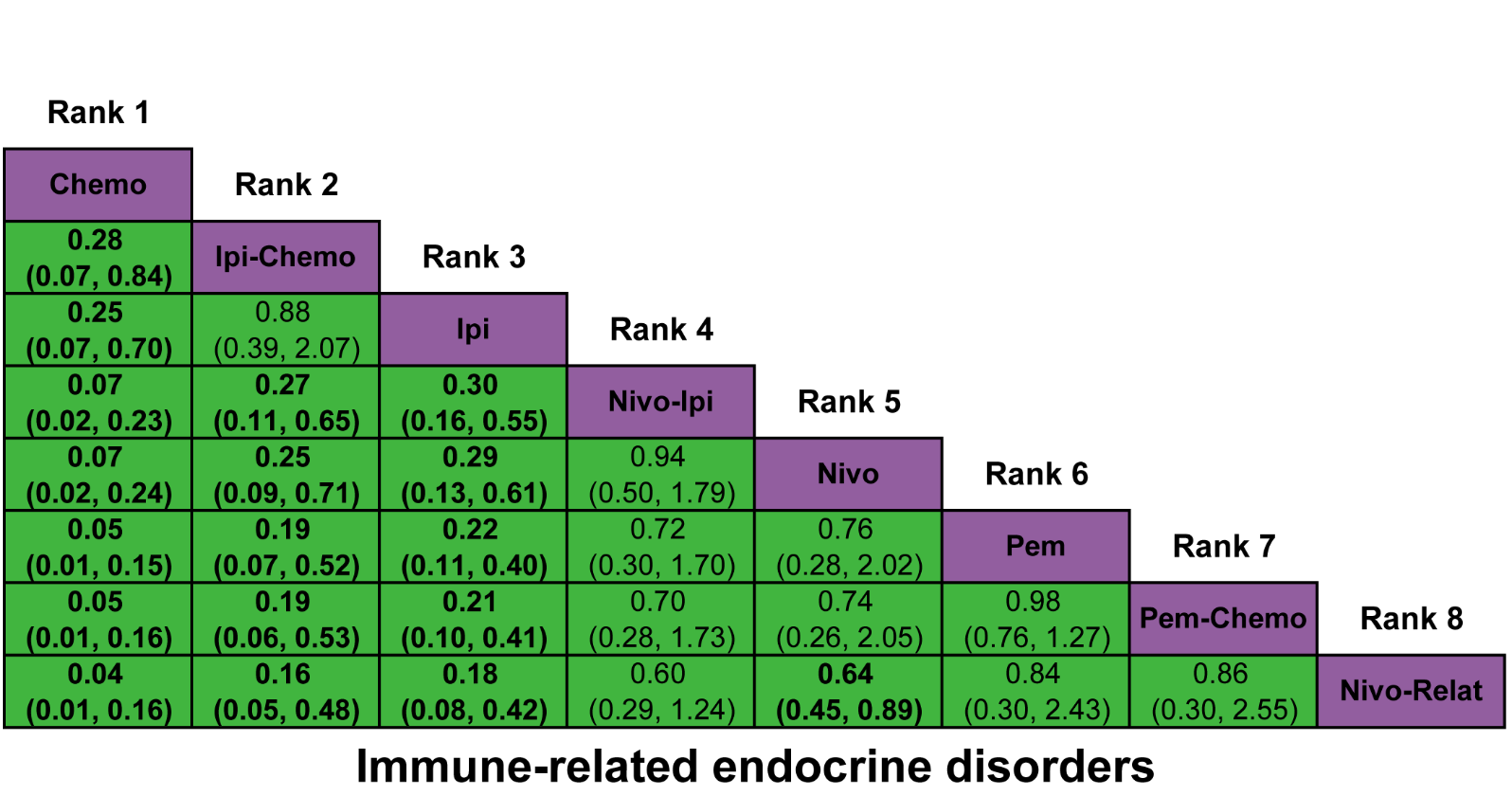


**(B) Gastrointestinal disorders**


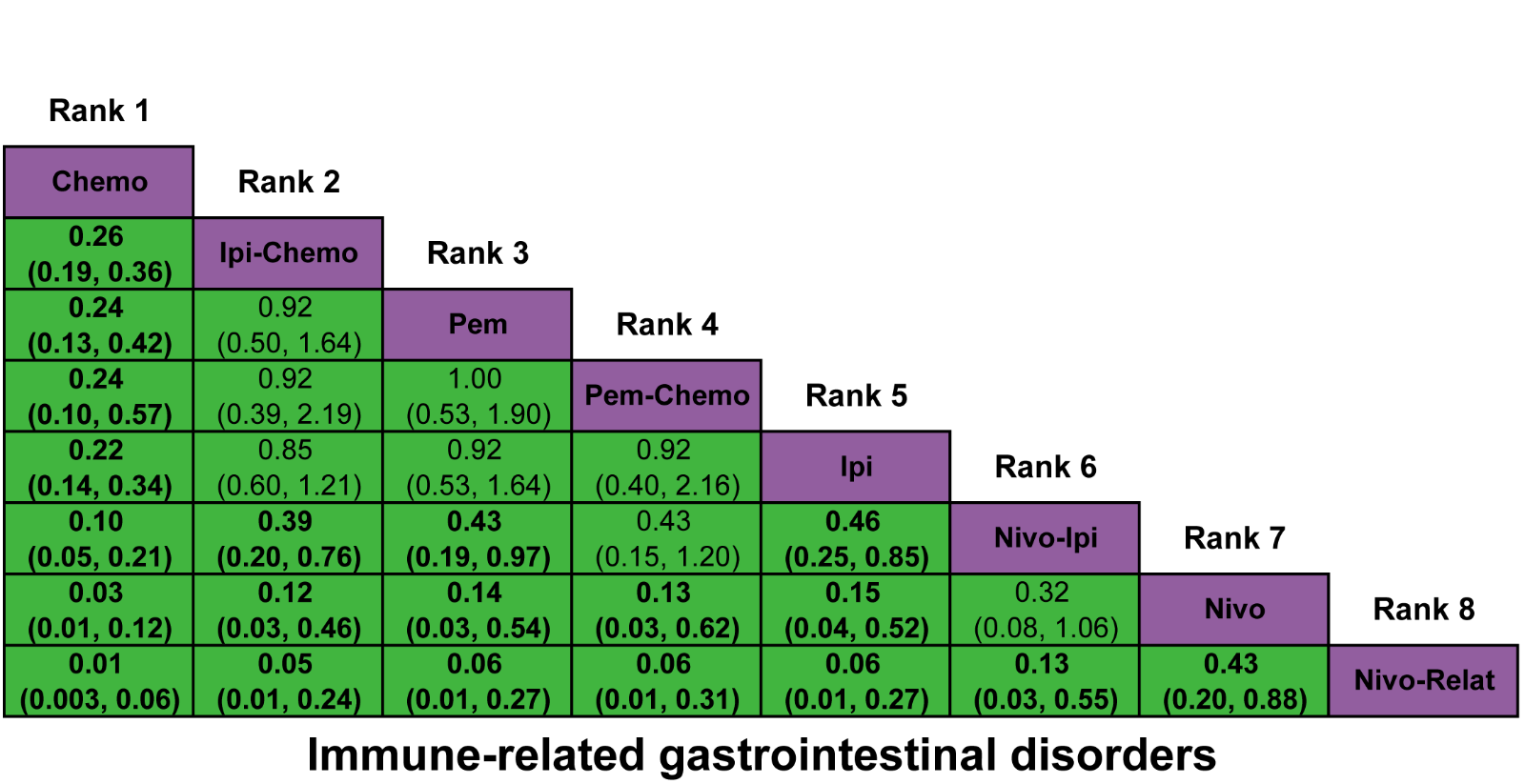


**(C) Respiratory, thoracic and mediastinal disorders**


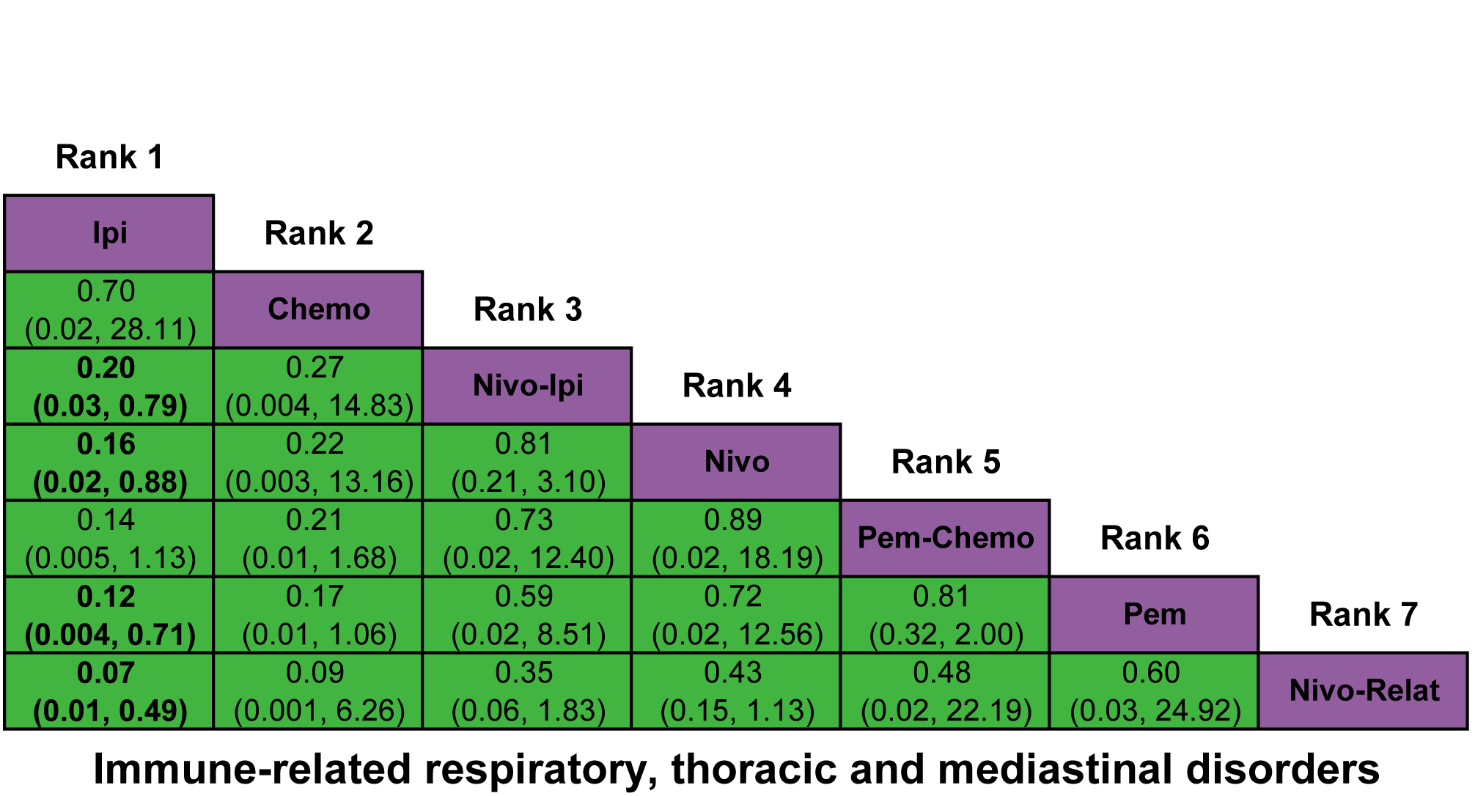


**(D) Skin and subcutaneous tissue disorders**


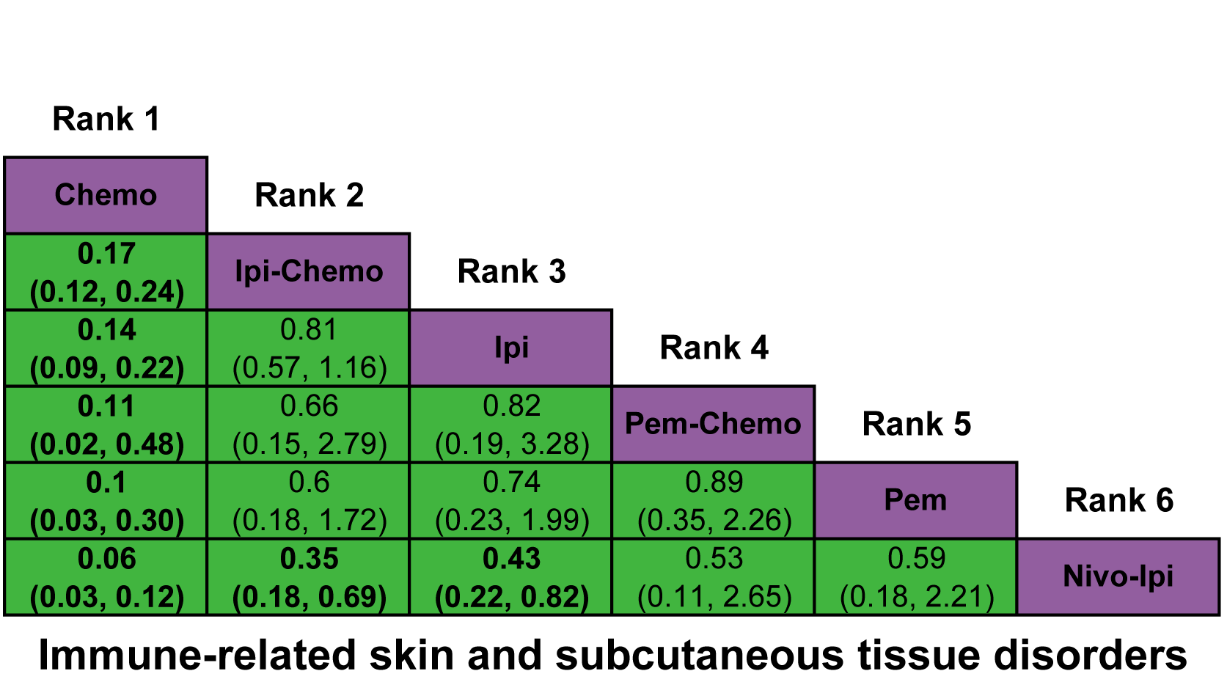


**Supplementary Figure S5**. Odds ratio (95% CrI) of system organ classes specific immune-related adverse events associated with each treatment regimen. (A) Endocrine disorders; (B) Gastrointestinal disorders; (C) Respiratory, thoracic and mediastinal disorders; (D) Skin and subcutaneous tissue disorders. Chemo, chemotherapy; Ipi: ipilimumab; Nivo: nivolumab; Pem: pembrolizumab; Relat: relatlimab.

**(A)**


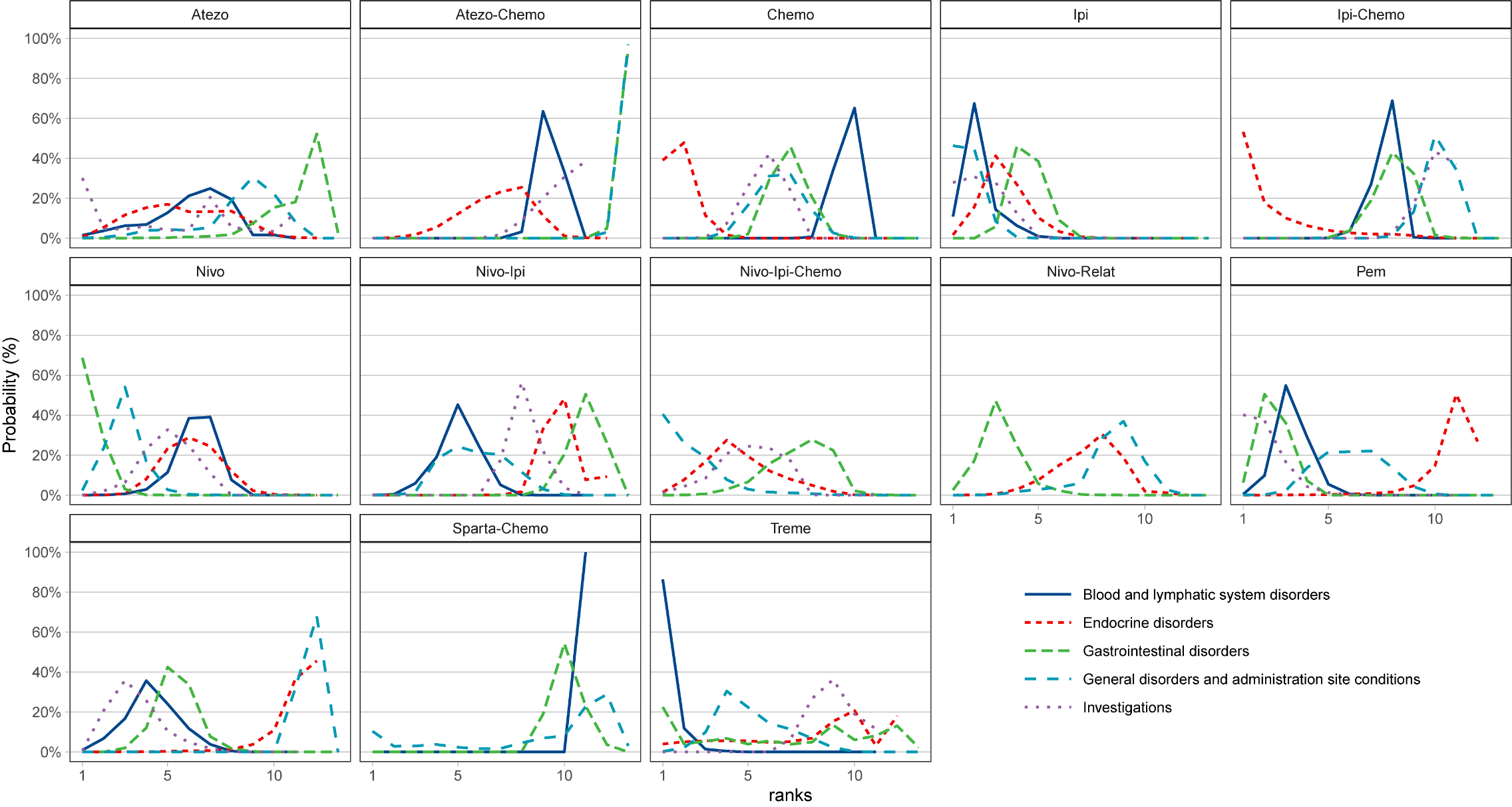


**(B)**


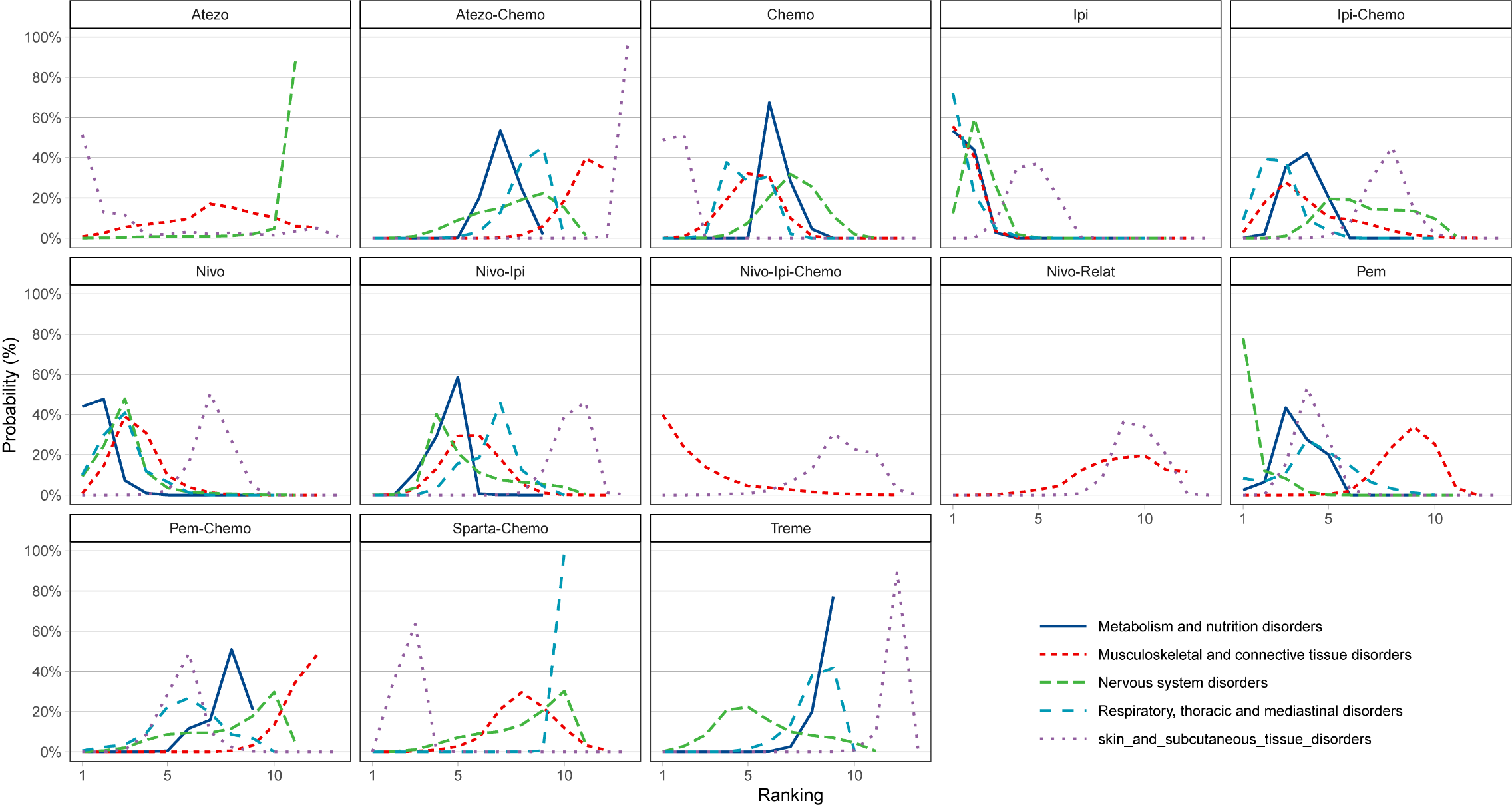


**Supplementary Figure S6.** Ranking of the probability of being the best treatment regimen in system organ classes specific treatment-related adverse events. (A) Ranking of the probability of the results of blood and lymphatic system disorders, endocrine disorders, gastrointestinal disorders, general disorders and administration site conditions and investigations; (B) Ranking of the probability of the results of metabolism and nutrition disorders, musculoskeletal and connective tissue disorders, nervous system disorders, respiratory, thoracic and mediastinal disorders and skin and subcutaneous tissue disorders. Atezo: atezolizumab; Chemo, chemotherapy; Ipi: ipilimumab; Nivo: nivolumab; Pem: pembrolizumab; Relat: relatlimab; Sparta: spartalizumab; Treme: tremelimumab.


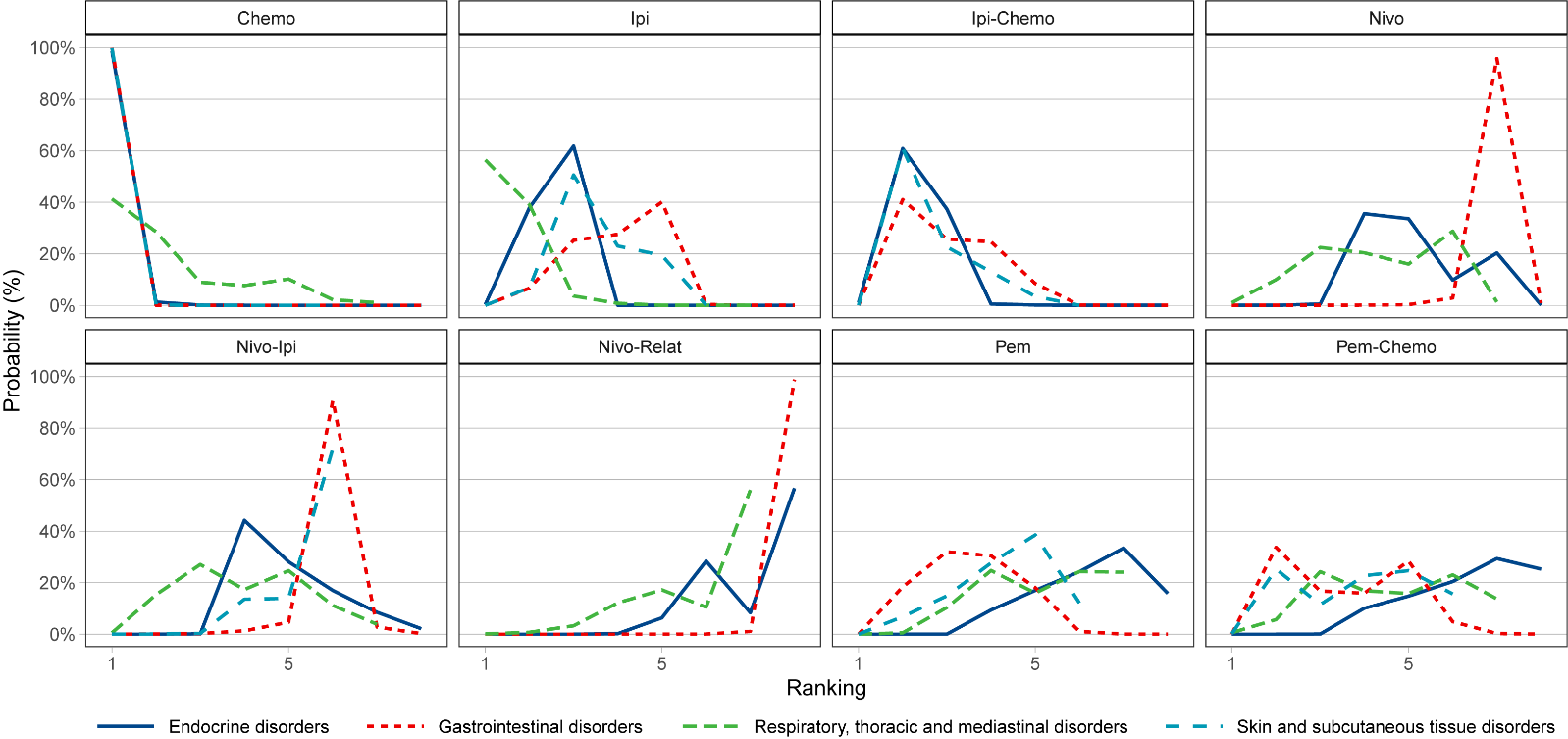


**Supplementary Figure S7.** Ranking of the probability of being the best treatment regimen in system organ classes specific immune-related adverse events. Included the results of endocrine disorders, gastrointestinal disorders, respiratory, thoracic and mediastinal disorders, skin and subcutaneous tissue disorders. Chemo, chemotherapy; Ipi: ipilimumab; Nivo: nivolumab; Pem: pembrolizumab; Relat: relatlimab.

**(A) any 3-5 treatment-related adverse events**


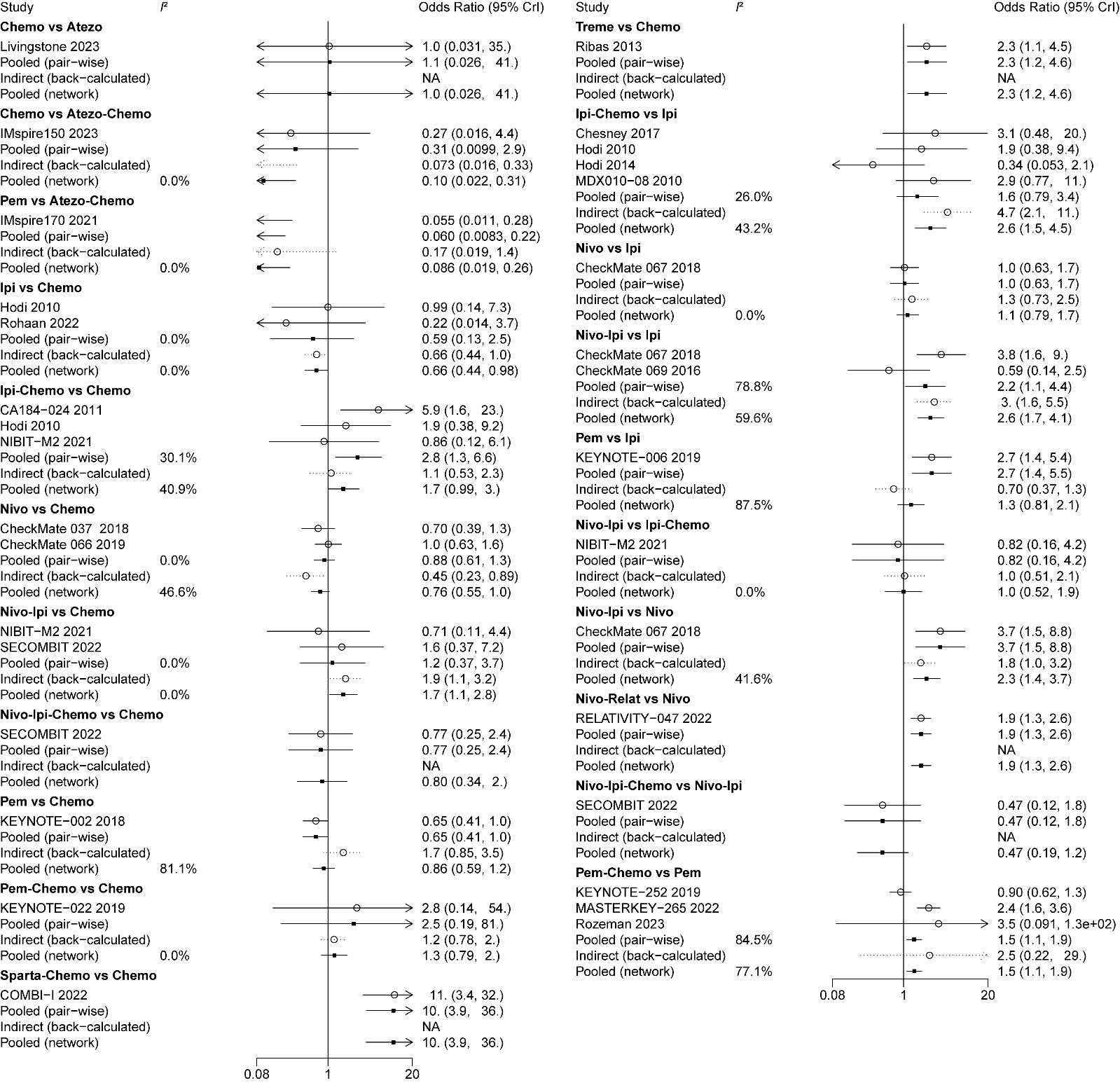


**(B) grades 3-5 treatment-related adverse events**


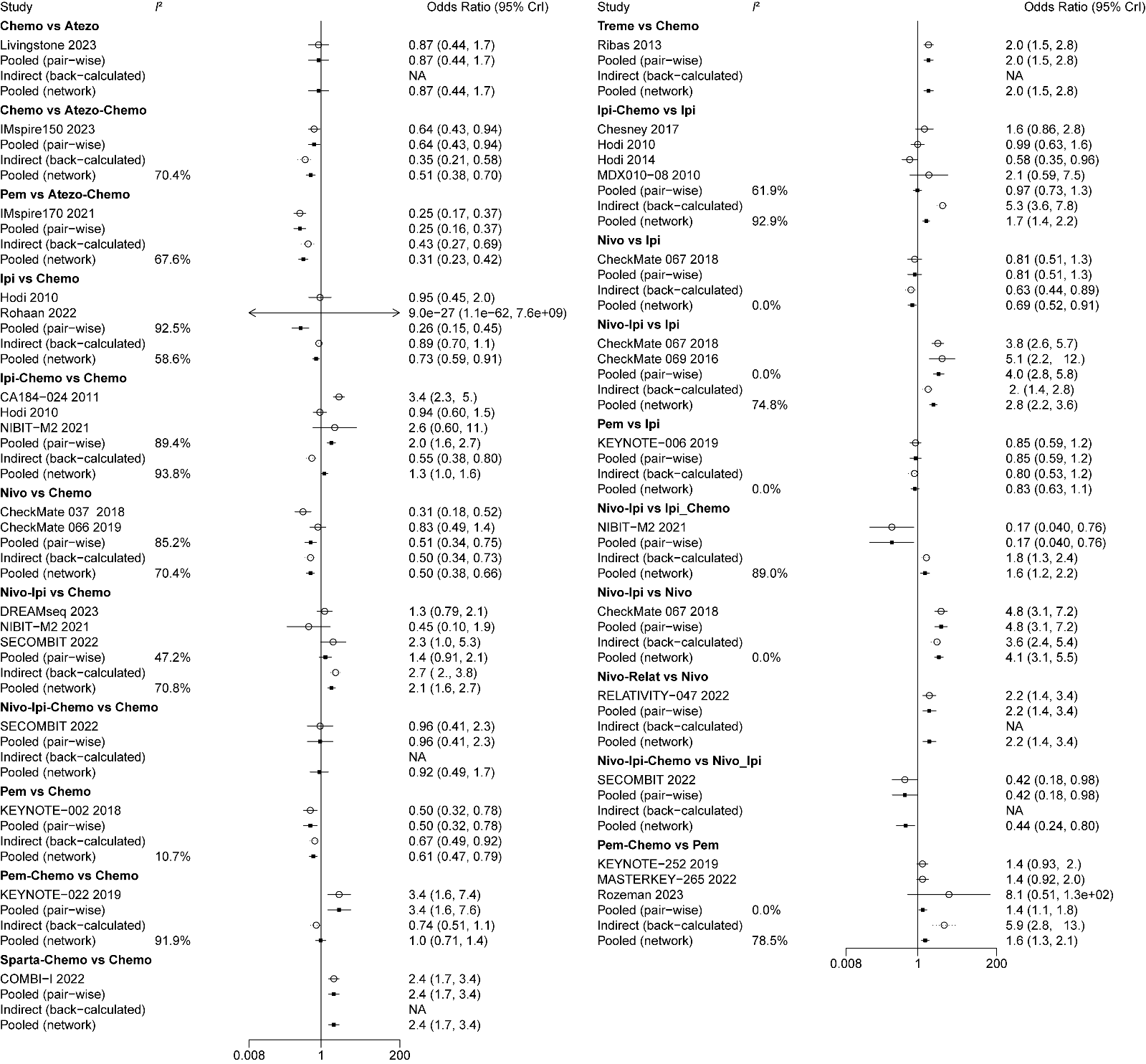


**(C) any 3-5** **immune-related adverse events**


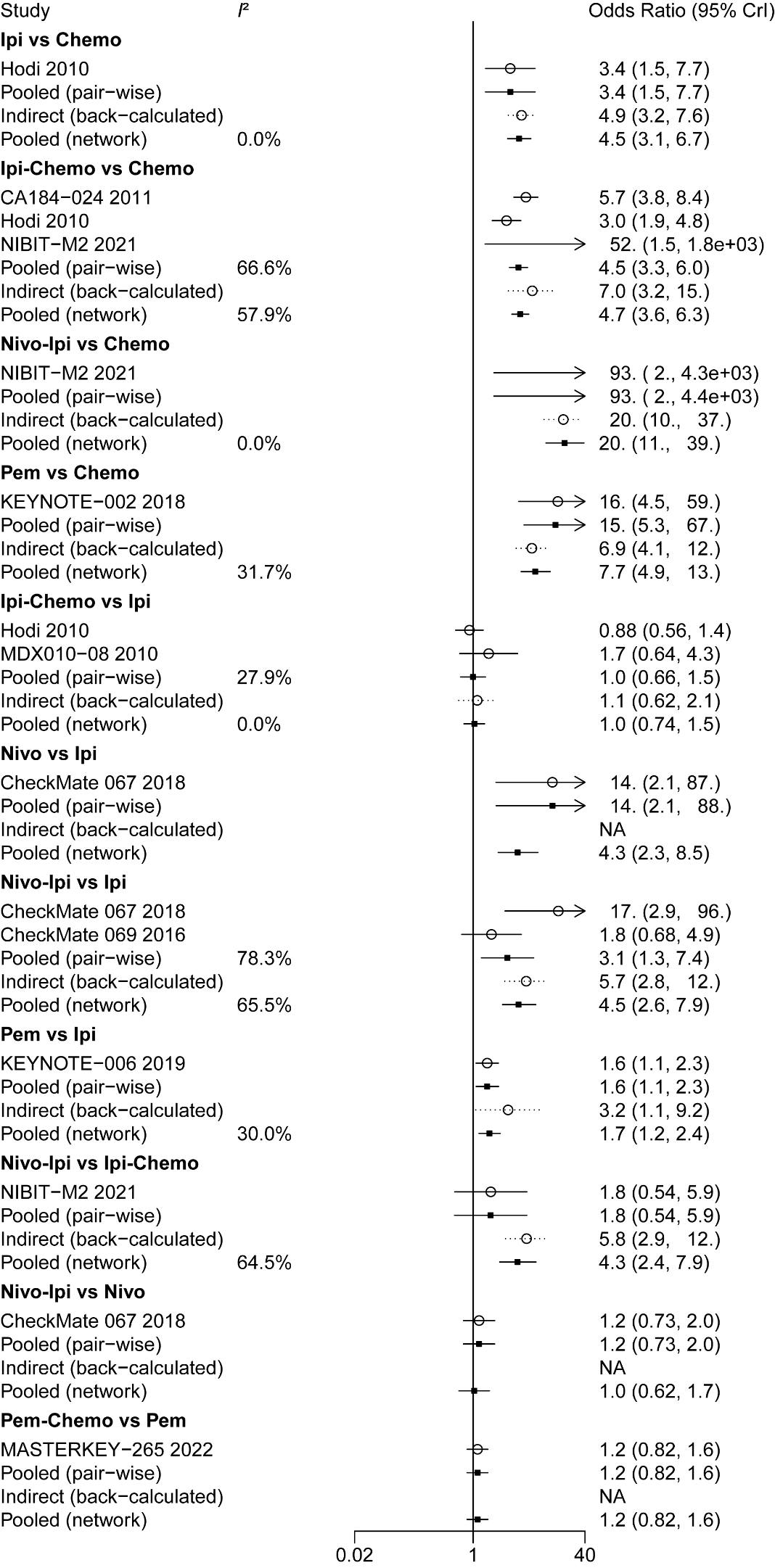


**(D) grade 3-5 immune-related adverse events**


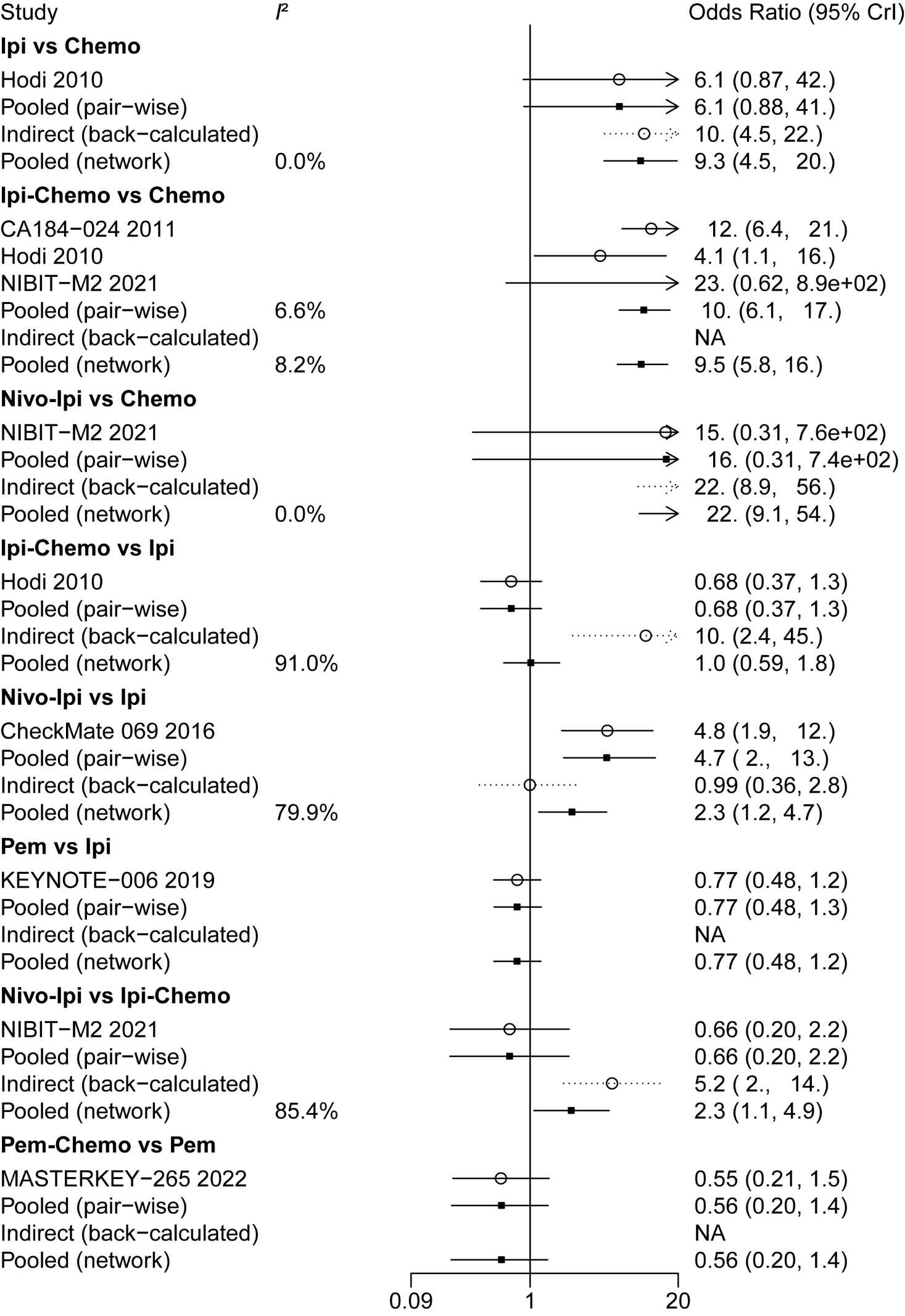


**Supplementary Figure S8.** Heterogeneity and inconsistency analysis of network meta-analysis results.


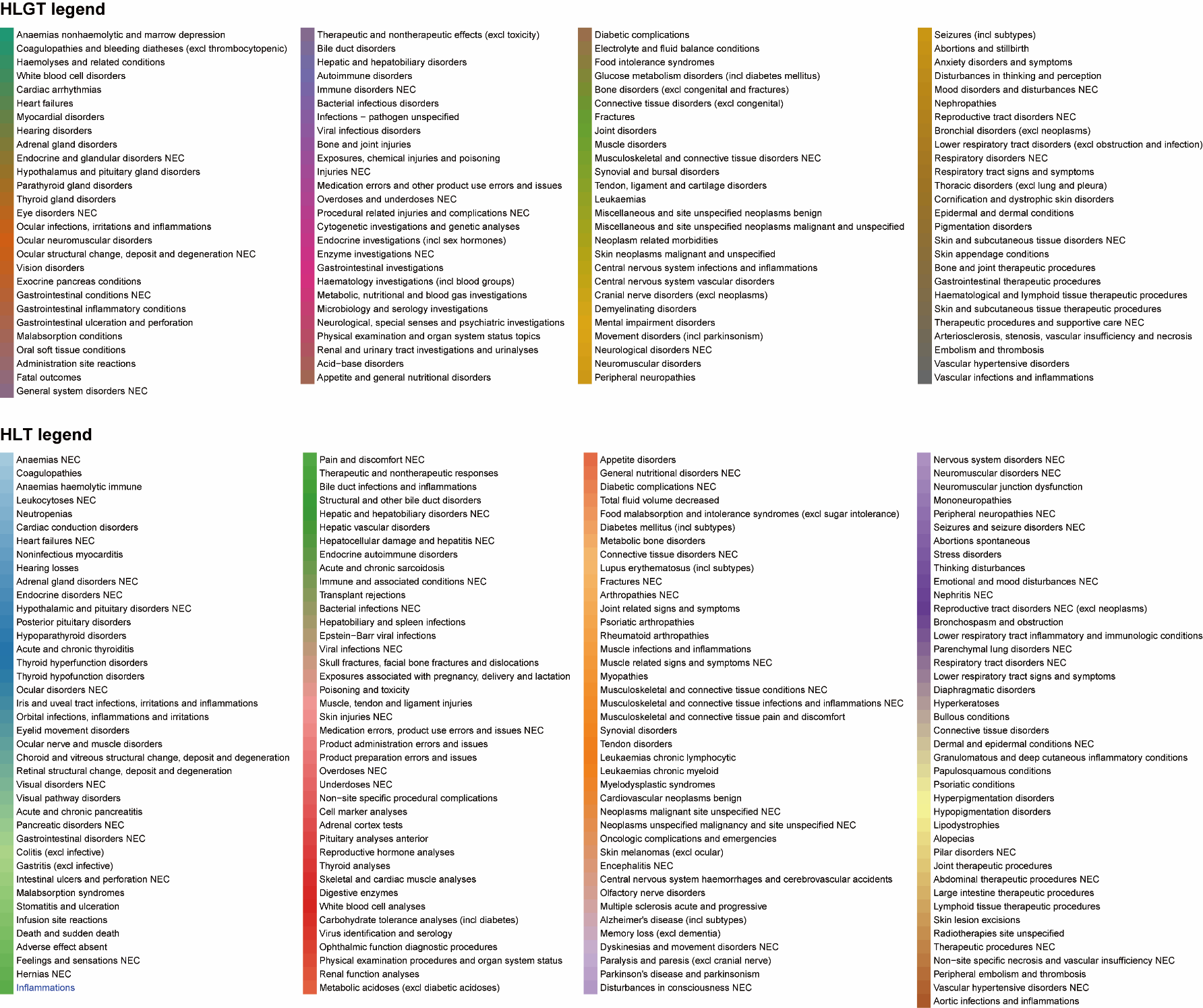


**Supplementary Figure S9.** HLGT and HLT legend of **Fig. 5**. HLGT, high level group term; HLT, high level term.

**Supplementary Table S1.** Characteristics of included randomized controlled trials for melanoma.

| Author & year | Study | Source | Registered ID | (Median Age/y) | Stage | Line of treatment | Arm 1 | Arm 2 | Arm 3 |
| --- | --- | --- | --- | --- | --- | --- | --- | --- | --- |
|  | (Phase, Design) | (year) | (Randomization) |  | (Male/Female) |  |  |  |  |
| Ascierto 2017 | CA184-169 (III, double-blind) | 2017 | NCT01515189 (1:1) | 365/362 (62/62) | III-IV (450/277) | 1/2L | Ipilimumab 10 mg/kg Q3W | Ipilimumab 3 mg/kg Q3W |  |
| Ascierto 2019 | KEYNOTE-022 (II, double-blind) | 2019 | NCT02130466 (1:1) | 60/60 (54/58) | III-IV (72/48) | 1L | Pembrolizumab 2 mg/kg Q3W + dabrafenib 150 mg BID + trametinib 2 mg QD | Dabrafenib 150 mg BID + trametinib 2 mg QD |  |
| Ascierto 2019b | CheckMate 066 (III, double-blind) | 2019 | NCT01721772 (1:1) | 210/218 (64/66) | III-IV (246/172) | 1L | Nivolumab 3 mg/kg Q2W | Dacarbazine 1000 mg/m2 Q3W |  |
| Ascierto 2022 | SECOMBIT (II, open-label) | 2022 | NCT02631447 (1:1:1) | 69/71/69（55/55/51） | III-IV (118/91) | 1L | Ipilimumab 3 mg/kg Q3W + nivolumab 1 mg/kg Q3W | Encorafenib 450 mg QD + Binimetinib 45 mg BID follow by ipilimumab 3 mg/kg Q3W + nivolumab 1 mg/kg Q3W | Encorafenib 450 mg QD + binimetinib 45 mg BID |
| Ascierto 2023 | IMspire150 (III, double-blind) | 2023 | NCT02908672 (1:1) | 256/258 (54/53.5) | III-IV (299/215) | 1L | Atezolizumab 840 mg Q2W + vemurafenib 720 mg BID + cobimetinib 60 mg QD | Vemurafenib 960 mg BID + cobimetinib 60 mg QD |  |
| Atkins 2023 | EA6134 (III, open-label) | 2023 | NCT02224781 (1:1) | 133/132 (61/61) | III-IV (167/98) | 1L | Nivolumab 1 mg/kg Q3W + ipilimumab 3 mg/kg Q3W follow by nivolumab 240 mg Q2W | Dabrafenib 150 mg BID + trametinib 2 mg QD |  |
| Chesney 2017 | Chesney 2017 (II, open-label) | 2017 | NCT01740297 (1:1) | 98/100 (65/64) | III-IV (117/81) | 1L | Ipilimumab 3 mg/kg Q3W + talimogene laherparepvec 106-108 plaque-forming units/mL Q3W | Ipilimumab 3 mg/kg Q3W |  |
| Chesney 2022 | MASTERKEY-265 (III, double-blind) | 2022 | NCT02263508(1:1) | 346/3346(64/64) | IIIB-IV (418/274) | 1L | Pembrolizumab 200 mg Q3W + T-VEC | Pembrolizumab 200 mg Q3W | |
| Dummer 2022 | COMBI-I (III, double-blind) | 2022 | NCT02967692 (1:1) | 267/265 (56/55) | III-IV (307/225) | 1L | Spartalizumab 400 mg Q4W + dabrafenib 150 mg BID + trametinib 2 mg QD | Dabrafenib 150 mg BID + trametinib 2 mg QD |  |
| Di Giacomo 2021 | NIBIT-M2 (II, open-label) | 2021 | NCT02460068(1:1:1) | 27/26/23 (57/60/56) | IV (48/28) | 1L | Ipilimumab 3 mg/kg Q3W + nivolumab 1 mg/kg Q3W | Ipilimumab 10 mg/kg + fotemustine100 mg/m2 QW | Fotemustine100 mg/m2 QW |
| Gogas 2021 | IMspire170 (III, open-label) | 2021 | NCT03273153 (1:1) | 222/224 (66/66) | III-IV (270/176) | 1L | Atezolizumab 840 mg Q2W + cobimetinib 60 mg QD | Pembrolizumab 200 mg Q3W |  |
| Hamid 2011 | CA184-004 (II, double-blind) | 2011 | NCT00261365 (1:1) | 40/42 (53.9/56.2) | III-IV (52/30) | 1L | Ipilimumab 3 mg/kg Q3W | Ipilimumab 10 mg/kg Q3W |  |
| Hamid 2018 | KEYNOTE-002 (II, double-blind) | 2018 | NCT01704287 (1:1:1) | 180/181/179 (62/60/63) | III-IV (327/213) | 2L | Pembrolizumab 2 mg/kg Q3W | Pembrolizumab 10 mg/kg Q3W | Investigator-choice chemotherapy |
| Hamid 2023 | Hamid 2023 (Ib, open-label) | 2023 | NCT02535078 | 43/13/29(59/52/58) | III-IV | 1L | Durvalumab 20 mg/kg + tremelimumab 10 mg/kg Q4W + tebentafusp | Durvalumab 20 mg/kg Q4W + tebentafusp | Tebentafusp |
| Hersh 2010 | MDX010-08 (II, open-label) | 2010 | NCT00050102 (1:1) | 37/35 (66/60) | IV (47/25) | 1L | Ipilimumab 3 mg/kg | Ipilimumab 3 mg/kg + dacarbazine 250 mg/m2 |  |
| Hodi 2010 | Hodi 2010 (III, double-blind) | 2010 | NCT00094653 (3:1:1) | 403/137/136 (55.6/56.8/57.4) | III-IV (401/275) | 2L | Ipilimumab 3 mg/kg Q3W + glycoprotein 100 Q3W | Ipilimumab 3 mg/kg Q3W | Glycoprotein 100 Q3W |
| Hodi 2014 | Hodi 2014 (II, single-blind) | 2014 | NCT01134614 (1:1) | 123/122 (61/64) | III-IV (163/82) | 1L | Ipilimumab 10 mg/kg Q3W + sargramostim 250 μg Q12W | Ipilimumab 10 mg/kg Q3W |  |
| Hodi 2016 | CheckMate 069 (II, double-blind) | 2016 | NCT01927419 (2:1) | 95/47 (64/67) | III-IV (95/47) | 1L | Nivolumab 1 mg/kg Q3W + ipilimumab 3 mg/kg Q3W | Ipilimumab 3 mg/kg Q3W |  |
| Hodi 2018 | CheckMate 067 (III, double-blind) | 2018 | NCT01844505 (1:1) | 314/316/315 (61/60/62) | III-IV (610/335) | 1L | Nivolumab 1 mg/kg Q3W + ipilimumab 3 mg/kg Q3W | Nivolumab 3 mg/kg Q2W | Ipilimumab 3 mg/kg Q3W |
| Larkin 2018 | CheckMate 037 (III, open-label) | 2018 | NCT01721746 (2:1) | 272/133 (59/62) | III-IV (261/144) | 2L | Nivolumab 3 mg/kg Q2W | Investigator-choice chemotherapy |  |
| Lebbé 2019 | CheckMate 511 (III-IV, double-blind) | 2019 | NCT02714218 (1:1) | 180/178 (58.5/58.5) | III-IV (206/152) | 1L | Nivolumab 3 mg/kg Q3W + ipilimumab 1 mg/kg Q3W | Nivolumab 1 mg/kg Q3W + ipilimumab 3 mg/kg Q3W |  |
| Livingstone 2023 | Livingstone 2023 (II, open-label) | 2023 | NCT02902029 (1:1) | 69/66(58/56) | IV (117/68) | 1L | Atezolizumab 1200 mg Q3W | Vemurafenib 960 mg QD D1-28 + cobimetinib 60 mg QD D1-21 of Q4W |  |
| Long 2019 | KEYNOTE-252 (III, double-blind) | 2019 | NCT02752074 (1:1) | 354/352 (64/63) | III-IV (423/283) | 1L | Pembrolizumab 200 mg Q3W + epacadostat 100 mg BID | Pembrolizumab 200 mg Q3W |  |
| Ribas 2013 | Ribas 2013 (III, open-label) | 2013 | NCT00257205 (1:1) | 328/327 (57/56) | III-IV (372/283) | 1L | Tremelimumab 15 mg/kg once every 90 days | Investigator-choice chemotherapy |  |
| Robert 2011 | CA184-024 (III, double-blind) | 2011 | NCT00324155 (1:1) | 250/252 (57.5/56.4) | III-IV (301/201) | 1L | Ipilimumab 10 mg/kg Q3W + dacarbazine 850 mg/m2 Q3W | Dacarbazine 850 mg/m2 Q3W |  |
| Robert 2014 | Robert 2014 (I, open-label) | 2014 | NCT01295827 (1:1) | 89/84 (57/60.7) | III-IV (105/68) | 2L | Pembrolizumab 2 mg/kg | Pembrolizumab 10 mg/kg |  |
| Robert 2019 | KEYNOTE-006 (III, open-label) | 2019 | NCT01866319 (1:1) | 556/278 (62/62) | III-IV (497/337) | 2L | Pembrolizumab 10 mg/kg Q2W or Q3W | Ipilimumab 3 mg/kg Q3W |  |
| Rohaan 2022 | Rohaan 2022 (III, open-label) | 2022 | NCT02278887 (1:1) | 84/84(59/59) | IIIC-IV (100/68) | 1L | Ipilimumab 3 mg/kg Q3W | Cyclophosphamide 60 mg/kg for 2 days + fludarabine 25 mg/AUC for 5 days |  |
| Rozeman 2023 | IMPemBra (II, open-label) | 2023 | NCT02625337(1:1:1:1) | 8/8/8/8(58/52/56/54) | III-IV (18/18) | 1L | Pembrolizumab 200 mg Q3W for 2 cycles + pembrolizumab 200 mg Q3W for 2 cycles + pembrolizumab 200 mg Q3W up to 2 years | Pembrolizumab 200 mg Q3W for 2 cycles + pembrolizumab 200 mg Q3W for 2 cycles+ dabrafenib 150 mg BID + trametinib 2 mg QD for 1W + pembrolizumab 200 mg Q3W up to 2 years | Pembrolizumab 200 mg Q3W for 2 cycles + pembrolizumab 200 mg Q3W for 2 cycles+ dabrafenib 150 mg BID + trametinib 2 mg QD for 2W + pembrolizumab 200 mg Q3W up to 2 years |
| Tawbi 2022 | RELATIVITY-047 (II-III, double-blind) | 2022 | NCT03470922 (1:1) | 355/359 (63/62) | III-IV (416/298) | 1L | Nivolumab 480 mg Q4W + relatlimab 160 mg Q4W | Nivolumab 480 mg Q4W |  |
| Wolchok 2010 | CA184-022 (II, double-blind) | 2010 | NCT00289640 (1:1:1) | 73/72/72 (59/59/56) | III-IV (144/73) | 2L | Ipilimumab 0.3 mg/kg Q3W | Ipilimumab 3 mg/kg Q3W | Ipilimumab 10 mg/kg Q3W |

**Supplementary Table S2.** Risk of bias assessment of the included studies.

| **Study** | **D1** | **D2** | **D3** | **D4** | **D5** | **Overall** |
| --- | --- | --- | --- | --- | --- | --- |
| CA184-169 | Low | Low | Low | Low | Low | Low |
| KEYNOTE-022 | Some concerns | Low | Low | Low | Low | Low |
| CheckMate 066 | Some concerns | Low | Low | Low | Low | Low |
| SECOMBIT | Some concerns | Some concerns | Low | Low | Low | Some concerns |
| IMspire150 | Low | Low | Low | Low | Low | Low |
| EA6134 | Some concerns | Some concerns | Low | Low | Low | Some concerns |
| Chesney 2017 | Some concerns | Some concerns | Low | Low | Low | Some concerns |
| MASTERKEY-265 | Some concerns | Low | Low | Low | Low | Low |
| COMBI-I | Low | Low | Low | Low | Low | Low |
| NIBIT-M2 | Some concerns | Low | Low | Low | Some concerns | Some concerns |
| IMspire170 | Low | Some concerns | Low | Low | Low | Some concerns |
| CA184-004 | Some concerns | Low | Some concerns | Low | Some concerns | Some concerns |
| KEYNOTE-002 | Low | Low | Low | Low | Low | Low |
| Hamid 2023 | Some concerns | Some concerns | Low | Low | Low | Some concerns |
| MDX010-08 | Some concerns | Some concerns | Some concerns | Low | Some concerns | Some concerns |
| Hodi 2010 | Some concerns | Low | Low | Low | Low | Low |
| Hodi 2014 | Low | Some concerns | Low | Low | Low | Some concerns |
| CheckMate 069 | Low | Low | Low | Low | Low | Low |
| CheckMate 067 | Low | Low | Low | Low | Low | Low |
| CheckMate 037 | Some concerns | Some concerns | Low | Low | Low | Some concerns |
| CheckMate 511 | Low | Low | Low | Low | Low | Low |
| Livingstone 2023 | Some concerns | Some concerns | Low | Low | Low | Some concerns |
| KEYNOTE-252 | Low | Low | Low | Low | Low | Low |
| Ribas 2013 | Some concerns | Some concerns | Low | Low | Low | Some concerns |
| CA184-024 | Low | Low | Low | Low | Low | Low |
| Robert 2014 | Low | Some concerns | Low | Low | Low | Some concerns |
| KEYNOTE-006 | Low | Some concerns | Low | Low | Low | Some concerns |
| Rohaan 2022 | Some concerns | Low | Low | Low | Low | Low |
| IMPemBra | Some concerns | Low | Low | Low | Low | Low |
| RELATIVITY-047 | Low | Low | Low | Low | Low | Low |
| CA184-022 | Low | Low | Low | Low | Some concerns | Some concerns |

D1: Bias due to randomisation.

D2: Bias due to deviations from intended intervention.

D3: Bias due to missing data.

D4: Bias due to outcome measurement.

D5: Bias due to selection of reported result.

**Supplementary Table S3.** Characteristics of reports with ICI-related adverse events in melanoma patients.

| **Clinical characteristics** | **Primary suspect (N = 22,706)** |
| --- | --- |
| **Gender** |  |
| Male | 12,419 (60%) |
| Female | 8,243 (40%) |
| Missing | 2,044 |
| **Age** |  |
| Median (IQR) | 64 (52, 73) |
| Missing | 5,699 |
| **Age group** |  |
| 18–64 | 8,844 (52%) |
| 65–75 | 4,765 (28%) |
| ≥75 | 3,398 (20%) |
| Missing | 5,699 |
| **Country** |  |
| United States of America | 9,694 (43%) |
| Japan | 2,204 (9.7%) |
| France | 2,040 (9.0%) |
| Germany | 1,732 (7.6%) |
| Australia | 991 (4.4%) |
| United Kingdom of Great Britain and Northern Ireland | 690 (3.0%) |
| Canada | 678 (3.0%) |
| Italy | 549 (2.4%) |
| Other country | 4,128 (18%) |
| **Received year** |  |
| 2012 | 263 (1.2%) |
| 2013 | 585 (2.6%) |
| 2014 | 1,080 (4.8%) |
| 2015 | 2,066 (9.1%) |
| 2016 | 2,299 (10%) |
| 2017 | 3,005 (13%) |
| 2018 | 3,038 (13%) |
| 2019 | 3,051 (13%) |
| 2020 | 2,276 (10%) |
| 2021 | 2,369 (10%) |
| 2022 | 2,188 (9.6%) |
| 2023 | 486 (2.1%) |
| **Case priority** |  |
| Direct | 346 (1.5%) |
| Expedited | 19,168 (84%) |
| Non-expedited | 3,192 (14%) |
| **Reporter typee** |  |
| Consumer | 6,202 (27%) |
| Healthcare professional | 16,449 (73%) |
| Lawyer | 13 (<0.1%) |
| Missing | 42 |
| **Treatment strategy** |  |
| Nivo-Ipi | 6,822 (30%) |
| Nivo | 5,393 (24%) |
| Ipi | 5,043 (22%) |
| Pem | 4,706 (21%) |
| Pem-Ipi | 405 (1.8%) |
| Atezo | 207 (0.9%) |
| Cemip | 29 (0.1%) |
| Ipi-Chemo | 27 (0.1%) |
| Nivo-Ipi-Chemo | 18 (<0.1%) |
| Pem-Chemo | 17 (<0.1%) |
| Durva | 10 (<0.1%) |
| Nivo-Chemo | 10 (<0.1%) |
| Atezo-Ipi | 7 (<0.1%) |
| Dostar | 3 (<0.1%) |
| Pem-Ipi-Chemo | 3 (<0.1%) |
| Durva-Treme | 2 (<0.1%) |
| Atezo-Chemo | 1 (<0.1%) |
| Atezo-Ipi-Treme | 1 (<0.1%) |
| Ave | 1 (<0.1%) |
| Durva-Ipi-Treme | 1 (<0.1%) |

**Supplementary Table S4.** The case number of different adverse events in melanoma cases receiving ICI treatment in FAERS database.

| **Adverse Event (preferred term)** | **Frequency** | **Percentage (%)** |
| --- | --- | --- |
| Malignant neoplasm progression | 3251 | 14.34% |
| Diarrhoea | 1750 | 7.72% |
| Death | 1666 | 7.35% |
| Colitis | 1410 | 6.22% |
| Fatigue | 1213 | 5.35% |
| Rash | 1073 | 4.73% |
| Pyrexia | 995 | 4.39% |
| Nausea | 778 | 3.43% |
| Hypophysitis | 757 | 3.34% |
| Off label use | 750 | 3.31% |
| Pruritus | 614 | 2.71% |
| Hypothyroidism | 602 | 2.65% |
| Decreased appetite | 591 | 2.61% |
| Headache | 571 | 2.52% |
| Dyspnoea | 565 | 2.49% |
| Vomiting | 563 | 2.48% |
| Acute kidney injury | 485 | 2.14% |
| Arthralgia | 477 | 2.10% |
| Asthenia | 475 | 2.09% |
| Weight decreased | 470 | 2.07% |
| Pneumonitis | 451 | 1.99% |
| Pneumonia | 441 | 1.94% |
| Adrenal insufficiency | 397 | 1.75% |
| Hepatitis | 378 | 1.67% |
| Malaise | 360 | 1.59% |
| Dehydration | 357 | 1.57% |
| Pain | 332 | 1.46% |
| Anaemia | 327 | 1.44% |
| Abdominal pain | 311 | 1.37% |
| Immune-mediated enterocolitis | 310 | 1.37% |
| Prescribed overdose | 307 | 1.35% |
| Hyperthyroidism | 297 | 1.31% |
| General physical health deterioration | 293 | 1.29% |
| Myocarditis | 291 | 1.28% |
| Liver disorder | 275 | 1.21% |
| Adverse event | 275 | 1.21% |
| Autoimmune hepatitis | 273 | 1.20% |
| Cough | 271 | 1.20% |
| Alanine aminotransferase increased | 266 | 1.17% |
| Sepsis | 264 | 1.16% |
| Myalgia | 263 | 1.16% |
| Aspartate aminotransferase increased | 259 | 1.14% |
| Hyponatraemia | 259 | 1.14% |
| Constipation | 257 | 1.13% |
| Hepatic function abnormal | 257 | 1.13% |
| Hypotension | 254 | 1.12% |
| Back pain | 249 | 1.10% |
| Muscular weakness | 243 | 1.07% |
| Dizziness | 234 | 1.03% |
| Vitiligo | 232 | 1.02% |
| Diabetes mellitus | 226 | 1.00% |
| Type 1 diabetes mellitus | 222 | 0.98% |
| Myositis | 221 | 0.97% |
| Diabetic ketoacidosis | 214 | 0.94% |
| Drug ineffective | 214 | 0.94% |
| Pemphigoid | 209 | 0.92% |
| Chills | 203 | 0.90% |
| Enterocolitis | 202 | 0.89% |
| Interstitial lung disease | 195 | 0.86% |
| Arthritis | 194 | 0.86% |
| Immune-mediated adverse reaction | 191 | 0.84% |
| Metastases to central nervous system | 189 | 0.83% |
| Autoimmune colitis | 188 | 0.83% |
| Pleural effusion | 184 | 0.81% |
| Thrombocytopenia | 183 | 0.81% |
| Renal failure | 182 | 0.80% |
| Immune-mediated hepatitis | 181 | 0.80% |
| Hypopituitarism | 179 | 0.79% |
| Uveitis | 176 | 0.78% |
| Atrial fibrillation | 172 | 0.76% |
| Rash maculo-papular | 169 | 0.75% |
| Pain in extremity | 166 | 0.73% |
| Neuropathy peripheral | 165 | 0.73% |
| Confusional state | 164 | 0.72% |
| Thyroiditis | 163 | 0.72% |
| Insomnia | 162 | 0.71% |
| Hepatic enzyme increased | 160 | 0.71% |
| Fall | 158 | 0.70% |
| Toxicity to various agents | 153 | 0.67% |
| Pancreatitis | 152 | 0.67% |
| Pulmonary embolism | 148 | 0.65% |
| Myasthenia gravis | 147 | 0.65% |
| Hypertension | 145 | 0.64% |
| Intentional product use issue | 145 | 0.64% |
| Hyperglycaemia | 144 | 0.64% |
| Urinary tract infection | 144 | 0.64% |
| Hypokalaemia | 143 | 0.63% |
| Inappropriate schedule of product administration | 140 | 0.62% |
| Respiratory failure | 137 | 0.60% |
| Dry mouth | 136 | 0.60% |
| Rash pruritic | 136 | 0.60% |
| Gait disturbance | 134 | 0.59% |
| Abdominal pain upper | 133 | 0.59% |
| Hepatotoxicity | 130 | 0.57% |
| Tubulointerstitial nephritis | 127 | 0.56% |
| Dysphagia | 125 | 0.55% |
| Lymphadenopathy | 125 | 0.55% |
| Erythema | 124 | 0.55% |
| Infusion related reaction | 123 | 0.54% |
| Prescribed underdose | 123 | 0.54% |
| Lipase increased | 122 | 0.54% |
| Somnolence | 122 | 0.54% |
| Blood alkaline phosphatase increased | 121 | 0.53% |
| Neutropenia | 120 | 0.53% |
| Thyroid disorder | 119 | 0.52% |
| Chest pain | 118 | 0.52% |
| Gastritis | 118 | 0.52% |
| Transaminases increased | 118 | 0.52% |
| Vision blurred | 117 | 0.52% |
| Cardiac failure | 114 | 0.50% |
| Seizure | 113 | 0.50% |
| Syncope | 113 | 0.50% |
| Weight increased | 112 | 0.49% |
| Gamma-glutamyltransferase increased | 111 | 0.49% |
| Visual impairment | 109 | 0.48% |
| Hepatic failure | 108 | 0.48% |
| Oedema peripheral | 108 | 0.48% |
| Platelet count decreased | 108 | 0.48% |
| Drug-induced liver injury | 108 | 0.48% |
| Dermatitis | 107 | 0.47% |
| Depression | 105 | 0.46% |
| Lung disorder | 105 | 0.46% |
| Blood creatine phosphokinase increased | 104 | 0.46% |
| Renal impairment | 103 | 0.45% |
| Blood creatinine increased | 101 | 0.45% |
| Cellulitis | 101 | 0.45% |
| Hepatocellular injury | 101 | 0.45% |
| Paraesthesia | 101 | 0.45% |
| Cholestasis | 100 | 0.44% |
| Rheumatoid arthritis | 100 | 0.44% |
| Intestinal perforation | 98 | 0.43% |
| Lymphocytic hypophysitis | 98 | 0.43% |
| Anxiety | 97 | 0.43% |
| Autoimmune thyroiditis | 97 | 0.43% |
| Multiple organ dysfunction syndrome | 97 | 0.43% |
| Hypoaesthesia | 96 | 0.42% |
| Condition aggravated | 94 | 0.41% |
| Encephalitis | 94 | 0.41% |
| Haemophagocytic lymphohistiocytosis | 94 | 0.41% |
| Gastrointestinal disorder | 93 | 0.41% |
| Infection | 93 | 0.41% |
| Rhabdomyolysis | 92 | 0.41% |
| Peripheral swelling | 92 | 0.41% |
| Immune-mediated myocarditis | 92 | 0.41% |
| C-reactive protein increased | 91 | 0.40% |
| Autoimmune disorder | 89 | 0.39% |
| Fulminant type 1 diabetes mellitus | 89 | 0.39% |
| Enteritis | 88 | 0.39% |
| Meningitis aseptic | 88 | 0.39% |
| Skin disorder | 88 | 0.39% |
| Dry skin | 85 | 0.37% |
| Large intestine perforation | 85 | 0.37% |
| Pancytopenia | 85 | 0.37% |
| Septic shock | 85 | 0.37% |
| Cerebrovascular accident | 82 | 0.36% |
| Myocardial infarction | 81 | 0.36% |
| Influenza like illness | 80 | 0.35% |
| Stomatitis | 80 | 0.35% |
| Facial paralysis | 79 | 0.35% |
| Hypothalamo-pituitary disorder | 79 | 0.35% |
| Leukoderma | 79 | 0.35% |
| Disease progression | 79 | 0.35% |
| Immune thrombocytopenia | 79 | 0.35% |
| Guillain-Barre syndrome | 78 | 0.34% |
| Polyarthritis | 78 | 0.34% |
| Cytokine release syndrome | 78 | 0.34% |
| Secondary adrenocortical insufficiency | 77 | 0.34% |
| Nephritis | 75 | 0.33% |
| Urticaria | 75 | 0.33% |
| Blood lactate dehydrogenase increased | 73 | 0.32% |
| Hypersensitivity | 72 | 0.32% |
| Deep vein thrombosis | 72 | 0.32% |
| Hospitalisation | 72 | 0.32% |
| Muscle spasms | 71 | 0.31% |
| Tremor | 71 | 0.31% |
| Liver function test increased | 71 | 0.31% |
| Immune-mediated lung disease | 71 | 0.31% |
| Alopecia | 70 | 0.31% |
| Ascites | 70 | 0.31% |
| Cerebral haemorrhage | 70 | 0.31% |
| Tumour pseudoprogression | 70 | 0.31% |
| Blood glucose increased | 69 | 0.30% |
| Cardiac arrest | 69 | 0.30% |
| Immune-mediated hepatic disorder | 69 | 0.30% |
| Disseminated intravascular coagulation | 68 | 0.30% |
| Feeling abnormal | 68 | 0.30% |
| Sarcoidosis | 68 | 0.30% |
| Brain oedema | 68 | 0.30% |
| Blood bilirubin increased | 67 | 0.30% |
| Eosinophilia | 67 | 0.30% |
| Metastases to liver | 67 | 0.30% |
| Musculoskeletal pain | 66 | 0.29% |
| Pericardial effusion | 66 | 0.29% |
| Amylase increased | 65 | 0.29% |
| Gastrointestinal haemorrhage | 65 | 0.29% |
| Abdominal discomfort | 64 | 0.28% |
| Haemoglobin decreased | 64 | 0.28% |
| Nasopharyngitis | 64 | 0.28% |
| Blood thyroid stimulating hormone increased | 63 | 0.28% |
| Conjunctivitis | 63 | 0.28% |
| Autoimmune haemolytic anaemia | 63 | 0.28% |
| COVID-19 | 63 | 0.28% |
| Hyperhidrosis | 62 | 0.27% |
| Tachycardia | 62 | 0.27% |
| Inflammation | 62 | 0.27% |
| Product use issue | 62 | 0.27% |
| Diplopia | 60 | 0.26% |
| Encephalopathy | 60 | 0.26% |
| Sjogren's syndrome | 59 | 0.26% |
| Vertigo | 59 | 0.26% |
| Lichenoid keratosis | 59 | 0.26% |
| Drug eruption | 58 | 0.26% |
| Iridocyclitis | 58 | 0.26% |
| Metastases to lung | 58 | 0.26% |
| Abdominal distension | 57 | 0.25% |
| Blood thyroid stimulating hormone decreased | 57 | 0.25% |
| Adrenocorticotropic hormone deficiency | 57 | 0.25% |
| Immune-mediated myositis | 57 | 0.25% |
| Oedema | 56 | 0.25% |
| Polymyalgia rheumatica | 56 | 0.25% |
| Immune-mediated thyroiditis | 56 | 0.25% |
| Blister | 55 | 0.24% |
| Herpes zoster | 55 | 0.24% |
| Hypoxia | 55 | 0.24% |
| Neck pain | 55 | 0.24% |
| Cholangitis | 54 | 0.24% |
| Colitis ulcerative | 54 | 0.24% |
| Influenza | 54 | 0.24% |
| Joint swelling | 54 | 0.24% |
| Eyelid ptosis | 53 | 0.23% |
| Lethargy | 53 | 0.23% |
| Neoplasm | 53 | 0.23% |
| Psoriasis | 53 | 0.23% |
| Meningitis | 52 | 0.23% |
| Optic neuritis | 52 | 0.23% |
| Oropharyngeal pain | 52 | 0.23% |
| Therapy partial responder | 52 | 0.23% |
| Cortisol decreased | 51 | 0.22% |
| Loss of consciousness | 51 | 0.22% |
| Mucosal inflammation | 51 | 0.22% |
| Swelling | 51 | 0.22% |
| Musculoskeletal stiffness | 51 | 0.22% |
| Dry eye | 50 | 0.22% |
| Metastatic malignant melanoma | 50 | 0.22% |
| Hepatic cytolysis | 50 | 0.22% |
| Bronchitis | 49 | 0.22% |
| Haematochezia | 49 | 0.22% |
| Troponin increased | 49 | 0.22% |
| Adverse drug reaction | 49 | 0.22% |
| Product use in unapproved indication | 49 | 0.22% |
| Product dose omission issue | 49 | 0.22% |
| Chest discomfort | 48 | 0.21% |
| Malignant melanoma | 48 | 0.21% |
| Thyroid function test abnormal | 48 | 0.21% |
| Balance disorder | 48 | 0.21% |
| Diverticulitis | 47 | 0.21% |
| Dyspnoea exertional | 47 | 0.21% |
| Erythema multiforme | 47 | 0.21% |
| Hypoalbuminaemia | 47 | 0.21% |
| Liver function test abnormal | 47 | 0.21% |
| Lymphocyte count decreased | 47 | 0.21% |
| Skin exfoliation | 47 | 0.21% |
| Haemorrhage | 47 | 0.21% |
| Memory impairment | 46 | 0.20% |
| Nervous system disorder | 46 | 0.20% |
| Oesophagitis | 46 | 0.20% |
| Drug reaction with eosinophilia and systemic symptoms | 46 | 0.20% |
| Rash macular | 45 | 0.20% |
| Rectal haemorrhage | 45 | 0.20% |
| No adverse event | 45 | 0.20% |
| Delirium | 44 | 0.19% |
| Epilepsy | 44 | 0.19% |
| Generalised oedema | 44 | 0.19% |
| Night sweats | 44 | 0.19% |
| Skin toxicity | 44 | 0.19% |
| Adrenal disorder | 43 | 0.19% |
| Adrenocortical insufficiency acute | 43 | 0.19% |
| Chromaturia | 43 | 0.19% |
| Hypoglycaemia | 43 | 0.19% |
| Ileus | 43 | 0.19% |
| Skin discolouration | 43 | 0.19% |
| Stevens-Johnson syndrome | 43 | 0.19% |
| Transfusion | 43 | 0.19% |
| Melanoma recurrent | 43 | 0.19% |
| Dysgeusia | 42 | 0.19% |
| Hypercalcaemia | 42 | 0.19% |
| Jaundice | 42 | 0.19% |
| Myopathy | 42 | 0.19% |
| Renal disorder | 42 | 0.19% |
| Sinusitis | 42 | 0.19% |
| Skin lesion | 42 | 0.19% |
| Sleep disorder | 42 | 0.19% |
| Cardiac disorder | 42 | 0.19% |
| Organising pneumonia | 42 | 0.19% |
| Autoimmune nephritis | 42 | 0.19% |
| Hyperkalaemia | 41 | 0.18% |
| Polyneuropathy | 41 | 0.18% |
| Agranulocytosis | 40 | 0.18% |
| Blindness | 40 | 0.18% |
| Eczema | 40 | 0.18% |
| Endocrine disorder | 40 | 0.18% |
| Gastroenteritis | 40 | 0.18% |
| Pneumonia aspiration | 40 | 0.18% |
| White blood cell count increased | 40 | 0.18% |
| Cataract | 39 | 0.17% |
| Dyspepsia | 39 | 0.17% |
| Dysphonia | 39 | 0.17% |
| Contusion | 39 | 0.17% |
| Immune-mediated pancreatitis | 39 | 0.17% |
| Immune-mediated hypothyroidism | 39 | 0.17% |
| Deafness | 38 | 0.17% |
| Duodenitis | 38 | 0.17% |
| Intestinal obstruction | 38 | 0.17% |
| Thrombosis | 38 | 0.17% |
| Autoimmune pancreatitis | 38 | 0.17% |
| Retinal detachment | 37 | 0.16% |
| Urinary retention | 37 | 0.16% |
| Therapy non-responder | 37 | 0.16% |
| Toxic skin eruption | 37 | 0.16% |
| Aphasia | 36 | 0.16% |
| Blood pressure increased | 36 | 0.16% |
| Leukocytosis | 36 | 0.16% |
| Neutrophil count decreased | 36 | 0.16% |
| Productive cough | 36 | 0.16% |
| Tumour lysis syndrome | 36 | 0.16% |
| Tumour haemorrhage | 36 | 0.16% |
| Pneumocystis jirovecii pneumonia | 36 | 0.16% |
| Haemolytic anaemia | 35 | 0.15% |
| Leukopenia | 35 | 0.15% |
| Rash erythematous | 35 | 0.15% |
| Metastasis | 35 | 0.15% |
| Aplasia pure red cell | 34 | 0.15% |
| Bone pain | 34 | 0.15% |
| Diarrhoea haemorrhagic | 34 | 0.15% |
| Erysipelas | 34 | 0.15% |
| Immune system disorder | 34 | 0.15% |
| Kidney transplant rejection | 34 | 0.15% |
| Myasthenic syndrome | 34 | 0.15% |
| Clostridium difficile infection | 34 | 0.15% |
| Blood pressure decreased | 33 | 0.15% |
| Flushing | 33 | 0.15% |
| Haemorrhage intracranial | 33 | 0.15% |
| Lower respiratory tract infection | 33 | 0.15% |
| Surgery | 33 | 0.15% |
| Toxic epidermal necrolysis | 33 | 0.15% |
| White blood cell count decreased | 33 | 0.15% |
| Embolism | 33 | 0.15% |
| Liver injury | 33 | 0.15% |
| Cholecystitis | 32 | 0.14% |
| Epistaxis | 32 | 0.14% |
| Peripheral sensory neuropathy | 32 | 0.14% |
| Speech disorder | 32 | 0.14% |
| Visual acuity reduced | 32 | 0.14% |
| Pulmonary mass | 32 | 0.14% |
| Pneumonia bacterial | 32 | 0.14% |
| Immune-mediated uveitis | 32 | 0.14% |
| Arrhythmia | 31 | 0.14% |
| Cardiomyopathy | 31 | 0.14% |
| Cytomegalovirus infection | 31 | 0.14% |
| Eosinophilic fasciitis | 31 | 0.14% |
| Pulmonary oedema | 31 | 0.14% |
| Rash papular | 31 | 0.14% |
| Tumour pain | 31 | 0.14% |
| Mental status changes | 31 | 0.14% |
| Hot flush | 31 | 0.14% |
| Appetite disorder | 31 | 0.14% |
| Brain neoplasm | 31 | 0.14% |
| Acute respiratory failure | 30 | 0.13% |
| Anaphylactic reaction | 30 | 0.13% |
| Coma | 30 | 0.13% |
| Disorientation | 30 | 0.13% |
| Dysarthria | 30 | 0.13% |
| Haematuria | 30 | 0.13% |
| Metastases to bone | 30 | 0.13% |
| Pericarditis | 30 | 0.13% |
| Peritonitis | 30 | 0.13% |
| Body temperature increased | 29 | 0.13% |
| Enterocolitis haemorrhagic | 29 | 0.13% |
| Eye pain | 29 | 0.13% |
| Hair colour changes | 29 | 0.13% |
| Hemiparesis | 29 | 0.13% |
| Hypocalcaemia | 29 | 0.13% |
| Pollakiuria | 29 | 0.13% |
| Rhinorrhoea | 29 | 0.13% |
| Serous retinal detachment | 29 | 0.13% |
| Weight fluctuation | 29 | 0.13% |
| Systemic inflammatory response syndrome | 29 | 0.13% |
| Autoimmune arthritis | 29 | 0.13% |
| Immune-mediated hypophysitis | 29 | 0.13% |
| Acute hepatic failure | 28 | 0.12% |
| Acute myocardial infarction | 28 | 0.12% |
| Altered state of consciousness | 28 | 0.12% |
| Amnesia | 28 | 0.12% |
| Chronic obstructive pulmonary disease | 28 | 0.12% |
| Eye disorder | 28 | 0.12% |
| Gait inability | 28 | 0.12% |
| Pancreatitis acute | 28 | 0.12% |
| Respiratory distress | 28 | 0.12% |
| Small intestinal obstruction | 28 | 0.12% |
| Vasculitis | 28 | 0.12% |
| Ventricular tachycardia | 28 | 0.12% |
| Hypoacusis | 28 | 0.12% |
| Gastrointestinal toxicity | 28 | 0.12% |
| Encephalitis autoimmune | 28 | 0.12% |
| Immune-mediated dermatitis | 28 | 0.12% |
| Clostridium difficile colitis | 27 | 0.12% |
| Cystitis | 27 | 0.12% |
| Dysuria | 27 | 0.12% |
| Eosinophil count increased | 27 | 0.12% |
| Febrile neutropenia | 27 | 0.12% |
| Gastrooesophageal reflux disease | 27 | 0.12% |
| Hypophosphataemia | 27 | 0.12% |
| Papilloedema | 27 | 0.12% |
| Peripheral motor neuropathy | 27 | 0.12% |
| Ejection fraction decreased | 27 | 0.12% |
| Maternal exposure during pregnancy | 27 | 0.12% |
| Arthropathy | 26 | 0.11% |
| Dermatitis bullous | 26 | 0.11% |
| Discomfort | 26 | 0.11% |
| Flatulence | 26 | 0.11% |
| Irritability | 26 | 0.11% |
| Lichen planus | 26 | 0.11% |
| Melaena | 26 | 0.11% |
| Neurotoxicity | 26 | 0.11% |
| Ocular hyperaemia | 26 | 0.11% |
| Palpitations | 26 | 0.11% |
| Pulmonary sarcoidosis | 26 | 0.11% |
| Swelling face | 26 | 0.11% |
| Addison's disease | 25 | 0.11% |
| Atrioventricular block complete | 25 | 0.11% |
| Basal cell carcinoma | 25 | 0.11% |
| Blood corticotrophin decreased | 25 | 0.11% |
| Eating disorder | 25 | 0.11% |
| Radiculopathy | 25 | 0.11% |
| Therapeutic response decreased | 25 | 0.11% |
| Tri-iodothyronine free decreased | 25 | 0.11% |
| Colitis microscopic | 25 | 0.11% |
| Cognitive disorder | 25 | 0.11% |
| Type 2 diabetes mellitus | 25 | 0.11% |
| Hypertransaminasaemia | 25 | 0.11% |
| Autoimmune hypothyroidism | 25 | 0.11% |
| Immune-mediated arthritis | 25 | 0.11% |
| Blood sodium decreased | 24 | 0.11% |
| Depressed level of consciousness | 24 | 0.11% |
| Gastrointestinal perforation | 24 | 0.11% |
| Granuloma | 24 | 0.11% |
| Haemorrhoids | 24 | 0.11% |
| Hypomagnesaemia | 24 | 0.11% |
| Ketoacidosis | 24 | 0.11% |
| Oral pain | 24 | 0.11% |
| Squamous cell carcinoma | 24 | 0.11% |
| Sudden death | 24 | 0.11% |
| Tinnitus | 24 | 0.11% |
| Wheezing | 24 | 0.11% |
| Pituitary enlargement | 24 | 0.11% |
| Inappropriate antidiuretic hormone secretion | 24 | 0.11% |
| Malnutrition | 24 | 0.11% |
| Ageusia | 23 | 0.10% |
| Blood potassium decreased | 23 | 0.10% |
| Cardio-respiratory arrest | 23 | 0.10% |
| Cerebral infarction | 23 | 0.10% |
| Diabetes insipidus | 23 | 0.10% |
| Electrolyte imbalance | 23 | 0.10% |
| Hepatitis acute | 23 | 0.10% |
| Mass | 23 | 0.10% |
| Myelitis transverse | 23 | 0.10% |
| Photosensitivity reaction | 23 | 0.10% |
| Skin reaction | 23 | 0.10% |
| Skin ulcer | 23 | 0.10% |
| Thirst | 23 | 0.10% |
| Thyrotoxic crisis | 23 | 0.10% |
| Central nervous system lesion | 23 | 0.10% |
| Incorrect dose administered | 23 | 0.10% |
| Chronic kidney disease | 23 | 0.10% |
| Pancreatic failure | 23 | 0.10% |
| Illness | 23 | 0.10% |
| Taste disorder | 23 | 0.10% |
| Angina pectoris | 22 | 0.10% |
| Ataxia | 22 | 0.10% |
| Dermatomyositis | 22 | 0.10% |
| Drug interaction | 22 | 0.10% |
| Metabolic acidosis | 22 | 0.10% |
| Overdose | 22 | 0.10% |
| Polymyositis | 22 | 0.10% |
| Staphylococcal infection | 22 | 0.10% |
| Drug intolerance | 22 | 0.10% |
| Pulmonary toxicity | 22 | 0.10% |
| Pigmentation disorder | 22 | 0.10% |
| Autoimmune myocarditis | 22 | 0.10% |
| Vogt-Koyanagi-Harada disease | 22 | 0.10% |
| Capillary leak syndrome | 21 | 0.09% |
| Carpal tunnel syndrome | 21 | 0.09% |
| Depressed mood | 21 | 0.09% |
| Haemolysis | 21 | 0.09% |
| Hyperthermia | 21 | 0.09% |
| Increased appetite | 21 | 0.09% |
| Intracranial tumour haemorrhage | 21 | 0.09% |
| Movement disorder | 21 | 0.09% |
| Oral candidiasis | 21 | 0.09% |
| Oxygen saturation decreased | 21 | 0.09% |
| Shock | 21 | 0.09% |
| Skin infection | 21 | 0.09% |
| Upper respiratory tract infection | 21 | 0.09% |
| Wound infection | 21 | 0.09% |
| Nodule | 21 | 0.09% |
| Cancer pain | 21 | 0.09% |
| Blood test abnormal | 21 | 0.09% |
| Bone marrow failure | 21 | 0.09% |
| Acute respiratory distress syndrome | 20 | 0.09% |
| Bradycardia | 20 | 0.09% |
| Chronic gastritis | 20 | 0.09% |
| Dementia | 20 | 0.09% |
| Feeling hot | 20 | 0.09% |
| Heart rate increased | 20 | 0.09% |
| Iritis | 20 | 0.09% |
| Metastases to skin | 20 | 0.09% |
| Migraine | 20 | 0.09% |
| Sinus tachycardia | 20 | 0.09% |
| Supraventricular tachycardia | 20 | 0.09% |
| Urinary incontinence | 20 | 0.09% |
| Viral infection | 20 | 0.09% |
| Acute coronary syndrome | 20 | 0.09% |
| Metastases to meninges | 20 | 0.09% |
| Radiation skin injury | 20 | 0.09% |
| Basedow's disease | 19 | 0.08% |
| Cholelithiasis | 19 | 0.08% |
| Dermatitis acneiform | 19 | 0.08% |
| Frequent bowel movements | 19 | 0.08% |
| Hyperbilirubinaemia | 19 | 0.08% |
| Lung infiltration | 19 | 0.08% |
| Metastases to lymph nodes | 19 | 0.08% |
| Myelitis | 19 | 0.08% |
| Myelodysplastic syndrome | 19 | 0.08% |
| Paralysis | 19 | 0.08% |
| Retinopathy | 19 | 0.08% |
| Skin hypopigmentation | 19 | 0.08% |
| Musculoskeletal chest pain | 19 | 0.08% |
| Deafness bilateral | 19 | 0.08% |
| Feeding disorder | 19 | 0.08% |
| Ischaemic stroke | 19 | 0.08% |
| Hypophagia | 19 | 0.08% |
| Treatment failure | 19 | 0.08% |
| Glucocorticoid deficiency | 19 | 0.08% |
| Acidosis | 18 | 0.08% |
| Acne | 18 | 0.08% |
| Erythema nodosum | 18 | 0.08% |
| Failure to thrive | 18 | 0.08% |
| Feeling cold | 18 | 0.08% |
| Gastric ulcer | 18 | 0.08% |
| Giant cell arteritis | 18 | 0.08% |
| Haemoptysis | 18 | 0.08% |
| Hallucination | 18 | 0.08% |
| Keratoacanthoma | 18 | 0.08% |
| Lymphopenia | 18 | 0.08% |
| Macular oedema | 18 | 0.08% |
| Neoplasm malignant | 18 | 0.08% |
| Nephropathy toxic | 18 | 0.08% |
| Respiratory disorder | 18 | 0.08% |
| Skin depigmentation | 18 | 0.08% |
| Mobility decreased | 18 | 0.08% |
| Gastric disorder | 18 | 0.08% |
| General physical condition abnormal | 18 | 0.08% |
| Respiratory tract infection | 18 | 0.08% |
| Immune-mediated nephritis | 18 | 0.08% |
| Atrioventricular block | 17 | 0.07% |
| Cardiac failure congestive | 17 | 0.07% |
| Crohn's disease | 17 | 0.07% |
| Diabetes mellitus inadequate control | 17 | 0.07% |
| Goitre | 17 | 0.07% |
| Groin pain | 17 | 0.07% |
| Hepatitis cholestatic | 17 | 0.07% |
| Lymphoedema | 17 | 0.07% |
| Mouth ulceration | 17 | 0.07% |
| Neuralgia | 17 | 0.07% |
| Neuritis | 17 | 0.07% |
| Papule | 17 | 0.07% |
| Psoriatic arthropathy | 17 | 0.07% |
| Pulmonary fibrosis | 17 | 0.07% |
| Therapeutic response unexpected | 17 | 0.07% |
| Transient ischaemic attack | 17 | 0.07% |
| Urosepsis | 17 | 0.07% |
| Dysstasia | 17 | 0.07% |
| Underdose | 17 | 0.07% |
| Neurological symptom | 17 | 0.07% |
| Limb discomfort | 17 | 0.07% |
| Neoplasm progression | 17 | 0.07% |
| Autoimmune neuropathy | 17 | 0.07% |
| Immune-mediated encephalitis | 17 | 0.07% |
| Central hypothyroidism | 17 | 0.07% |
| Agitation | 16 | 0.07% |
| Angioedema | 16 | 0.07% |
| Asthma | 16 | 0.07% |
| Atrial flutter | 16 | 0.07% |
| Eye swelling | 16 | 0.07% |
| Ileus paralytic | 16 | 0.07% |
| Nephrotic syndrome | 16 | 0.07% |
| Pneumothorax | 16 | 0.07% |
| Presyncope | 16 | 0.07% |
| Red blood cell count decreased | 16 | 0.07% |
| Renal tubular necrosis | 16 | 0.07% |
| Splenomegaly | 16 | 0.07% |
| Upper gastrointestinal haemorrhage | 16 | 0.07% |
| Dermatitis psoriasiform | 16 | 0.07% |
| Demyelinating polyneuropathy | 16 | 0.07% |
| Pancreatic enzymes increased | 16 | 0.07% |
| Skin mass | 16 | 0.07% |
| Rheumatic disorder | 16 | 0.07% |
| Atelectasis | 15 | 0.07% |
| Cachexia | 15 | 0.07% |
| Coronary artery disease | 15 | 0.07% |
| Deafness neurosensory | 15 | 0.07% |
| Disturbance in attention | 15 | 0.07% |
| Face oedema | 15 | 0.07% |
| Hepatic pain | 15 | 0.07% |
| Hip fracture | 15 | 0.07% |
| Hyperpyrexia | 15 | 0.07% |
| Impaired gastric emptying | 15 | 0.07% |
| International normalised ratio increased | 15 | 0.07% |
| Macule | 15 | 0.07% |
| Nephrolithiasis | 15 | 0.07% |
| Ophthalmoplegia | 15 | 0.07% |
| Osteoarthritis | 15 | 0.07% |
| Premature delivery | 15 | 0.07% |
| Primary hypothyroidism | 15 | 0.07% |
| Neoplasm recurrence | 15 | 0.07% |
| Faeces soft | 15 | 0.07% |
| Blood albumin decreased | 14 | 0.06% |
| Blood testosterone decreased | 14 | 0.06% |
| Bronchopulmonary aspergillosis | 14 | 0.06% |
| Chorioretinitis | 14 | 0.06% |
| Coagulopathy | 14 | 0.06% |
| Drug hypersensitivity | 14 | 0.06% |
| Ear pain | 14 | 0.06% |
| Gastrointestinal necrosis | 14 | 0.06% |
| Haematocrit decreased | 14 | 0.06% |
| Hypersomnia | 14 | 0.06% |
| Intussusception | 14 | 0.06% |
| Mood altered | 14 | 0.06% |
| Proteinuria | 14 | 0.06% |
| Pulmonary granuloma | 14 | 0.06% |
| Pyelonephritis | 14 | 0.06% |
| Subdural haematoma | 14 | 0.06% |
| Mental disorder | 14 | 0.06% |
| Non-cardiac chest pain | 14 | 0.06% |
| Poor quality sleep | 14 | 0.06% |
| Device related infection | 14 | 0.06% |
| Radiation necrosis | 14 | 0.06% |
| Candida infection | 14 | 0.06% |
| COVID-19 pneumonia | 14 | 0.06% |
| Aplastic anaemia | 13 | 0.06% |
| Blood thyroid stimulating hormone abnormal | 13 | 0.06% |
| Breast cancer | 13 | 0.06% |
| Cardiogenic shock | 13 | 0.06% |
| Eye inflammation | 13 | 0.06% |
| Hepatitis fulminant | 13 | 0.06% |
| Hyperuricaemia | 13 | 0.06% |
| Impaired healing | 13 | 0.06% |
| Joint stiffness | 13 | 0.06% |
| Keratitis | 13 | 0.06% |
| Muscle atrophy | 13 | 0.06% |
| Muscle disorder | 13 | 0.06% |
| Oesophageal candidiasis | 13 | 0.06% |
| Orthostatic hypotension | 13 | 0.06% |
| Pancreatic atrophy | 13 | 0.06% |
| Scar | 13 | 0.06% |
| Sialoadenitis | 13 | 0.06% |
| Squamous cell carcinoma of skin | 13 | 0.06% |
| Stress | 13 | 0.06% |
| Throat irritation | 13 | 0.06% |
| Cytomegalovirus colitis | 13 | 0.06% |
| Meningoradiculitis | 13 | 0.06% |
| Facial paresis | 13 | 0.06% |
| Cell death | 13 | 0.06% |
| Troponin I increased | 13 | 0.06% |
| Troponin T increased | 13 | 0.06% |
| Drug resistance | 13 | 0.06% |
| Bacterial infection | 13 | 0.06% |
| Optic neuropathy | 13 | 0.06% |
| Hepatic lesion | 13 | 0.06% |
| Seronegative arthritis | 13 | 0.06% |
| Immune-mediated gastritis | 13 | 0.06% |
| Immune-mediated adrenal insufficiency | 13 | 0.06% |
| Abdominal pain lower | 12 | 0.05% |
| Acute febrile neutrophilic dermatosis | 12 | 0.05% |
| Blood creatine phosphokinase MB increased | 12 | 0.05% |
| Blood glucose abnormal | 12 | 0.05% |
| Blood iron decreased | 12 | 0.05% |
| Burning sensation | 12 | 0.05% |
| Cheilitis | 12 | 0.05% |
| Emotional disorder | 12 | 0.05% |
| Epstein-Barr virus infection | 12 | 0.05% |
| Fluid retention | 12 | 0.05% |
| Haematoma | 12 | 0.05% |
| Head injury | 12 | 0.05% |
| Intracranial pressure increased | 12 | 0.05% |
| Laboratory test abnormal | 12 | 0.05% |
| Leukoencephalopathy | 12 | 0.05% |
| Melanocytic naevus | 12 | 0.05% |
| Metastases to spine | 12 | 0.05% |
| Morphoea | 12 | 0.05% |
| Nasal congestion | 12 | 0.05% |
| Neutrophil count increased | 12 | 0.05% |
| Palmar-plantar erythrodysaesthesia syndrome | 12 | 0.05% |
| Polyuria | 12 | 0.05% |
| Synovitis | 12 | 0.05% |
| Ulcer | 12 | 0.05% |
| Cardiotoxicity | 12 | 0.05% |
| Performance status decreased | 12 | 0.05% |
| Wound | 12 | 0.05% |
| Chronic inflammatory demyelinating polyradiculoneuropathy | 12 | 0.05% |
| Hypogonadism | 12 | 0.05% |
| Adrenalitis | 12 | 0.05% |
| Gastrointestinal inflammation | 12 | 0.05% |
| Therapeutic product effect incomplete | 12 | 0.05% |
| Autoimmune myositis | 12 | 0.05% |
| Bacteraemia | 11 | 0.05% |
| Blindness unilateral | 11 | 0.05% |
| Blood urea increased | 11 | 0.05% |
| Cortisol increased | 11 | 0.05% |
| Cutaneous sarcoidosis | 11 | 0.05% |
| Duodenal ulcer | 11 | 0.05% |
| Gout | 11 | 0.05% |
| Hepatic encephalopathy | 11 | 0.05% |
| Hepatic steatosis | 11 | 0.05% |
| Hypersensitivity vasculitis | 11 | 0.05% |
| Incontinence | 11 | 0.05% |
| Iron deficiency anaemia | 11 | 0.05% |
| Jaundice cholestatic | 11 | 0.05% |
| Joint effusion | 11 | 0.05% |
| Lactic acidosis | 11 | 0.05% |
| Lymphadenopathy mediastinal | 11 | 0.05% |
| Lymphoma | 11 | 0.05% |
| Pain of skin | 11 | 0.05% |
| Pallor | 11 | 0.05% |
| Pleurisy | 11 | 0.05% |
| Respiratory arrest | 11 | 0.05% |
| Skin hyperpigmentation | 11 | 0.05% |
| Status epilepticus | 11 | 0.05% |
| Tenosynovitis | 11 | 0.05% |
| Tooth disorder | 11 | 0.05% |
| Cerebral disorder | 11 | 0.05% |
| Staphylococcal sepsis | 11 | 0.05% |
| Subacute cutaneous lupus erythematosus | 11 | 0.05% |
| Bicytopenia | 11 | 0.05% |
| Motor dysfunction | 11 | 0.05% |
| Blood disorder | 11 | 0.05% |
| Colectomy | 11 | 0.05% |
| Hepatobiliary disease | 11 | 0.05% |
| Ulcerative keratitis | 11 | 0.05% |
| Stress cardiomyopathy | 11 | 0.05% |
| Oral herpes | 11 | 0.05% |
| Adverse reaction | 11 | 0.05% |
| Prerenal failure | 11 | 0.05% |
| Limbic encephalitis | 11 | 0.05% |
| Abnormal faeces | 10 | 0.04% |
| Aspiration | 10 | 0.04% |
| Blood pressure abnormal | 10 | 0.04% |
| Cholangitis sclerosing | 10 | 0.04% |
| Chronic lymphocytic leukaemia | 10 | 0.04% |
| Chronic myeloid leukaemia | 10 | 0.04% |
| Extravasation | 10 | 0.04% |
| Eye irritation | 10 | 0.04% |
| Femur fracture | 10 | 0.04% |
| Flank pain | 10 | 0.04% |
| Gastrointestinal infection | 10 | 0.04% |
| Hepatomegaly | 10 | 0.04% |
| Lip swelling | 10 | 0.04% |
| Nephropathy | 10 | 0.04% |
| Normochromic normocytic anaemia | 10 | 0.04% |
| Osteoporosis | 10 | 0.04% |
| Pancreatic disorder | 10 | 0.04% |
| Panniculitis | 10 | 0.04% |
| Parosmia | 10 | 0.04% |
| Polyserositis | 10 | 0.04% |
| Portal vein thrombosis | 10 | 0.04% |
| Radiotherapy | 10 | 0.04% |
| Rash pustular | 10 | 0.04% |
| Rash vesicular | 10 | 0.04% |
| Raynaud's phenomenon | 10 | 0.04% |
| Retching | 10 | 0.04% |
| Sciatica | 10 | 0.04% |
| Scleroderma | 10 | 0.04% |
| Thrombotic microangiopathy | 10 | 0.04% |
| Transplant rejection | 10 | 0.04% |
| Venoocclusive liver disease | 10 | 0.04% |
| Venous thrombosis | 10 | 0.04% |
| VIth nerve paralysis | 10 | 0.04% |
| Vitreous floaters | 10 | 0.04% |
| Shock haemorrhagic | 10 | 0.04% |
| Lower gastrointestinal haemorrhage | 10 | 0.04% |
| Platelet count increased | 10 | 0.04% |
| Musculoskeletal discomfort | 10 | 0.04% |
| Abdominal sepsis | 10 | 0.04% |
| Cystoid macular oedema | 10 | 0.04% |
| Angiopathy | 10 | 0.04% |
| Gastrointestinal motility disorder | 10 | 0.04% |
| Abnormal behaviour | 10 | 0.04% |
| Ill-defined disorder | 10 | 0.04% |
| Lung neoplasm | 10 | 0.04% |
| Myasthenia gravis crisis | 10 | 0.04% |
| Chorioretinopathy | 10 | 0.04% |
| Skin haemorrhage | 10 | 0.04% |
| Diabetic metabolic decompensation | 10 | 0.04% |
| Autoimmune dermatitis | 10 | 0.04% |
| Autoimmune uveitis | 10 | 0.04% |
| Thyroid hormones decreased | 10 | 0.04% |
| Anal incontinence | 10 | 0.04% |
| Product administration error | 10 | 0.04% |
| Red blood cell transfusion | 10 | 0.04% |
| Alanine aminotransferase abnormal | 9 | 0.04% |
| Alveolitis | 9 | 0.04% |
| Anaphylactic shock | 9 | 0.04% |
| Aphthous ulcer | 9 | 0.04% |
| Appendicitis | 9 | 0.04% |
| Blood potassium increased | 9 | 0.04% |
| Cardiac tamponade | 9 | 0.04% |
| Cholecystitis acute | 9 | 0.04% |
| Choroidal detachment | 9 | 0.04% |
| Colon cancer | 9 | 0.04% |
| Demyelination | 9 | 0.04% |
| Dermatitis exfoliative | 9 | 0.04% |
| Diaphragmatic paralysis | 9 | 0.04% |
| Dyskinesia | 9 | 0.04% |
| Electrocardiogram QT prolonged | 9 | 0.04% |
| Eosinophilic pneumonia | 9 | 0.04% |
| Faeces discoloured | 9 | 0.04% |
| Fasciitis | 9 | 0.04% |
| Fungal infection | 9 | 0.04% |
| Generalised tonic-clonic seizure | 9 | 0.04% |
| Glaucoma | 9 | 0.04% |
| Haematemesis | 9 | 0.04% |
| Hydronephrosis | 9 | 0.04% |
| Hypopnoea | 9 | 0.04% |
| Hypovolaemia | 9 | 0.04% |
| Lacrimation increased | 9 | 0.04% |
| Lymphadenitis | 9 | 0.04% |
| Megacolon | 9 | 0.04% |
| Monocyte count increased | 9 | 0.04% |
| Multiple sclerosis | 9 | 0.04% |
| Muscle twitching | 9 | 0.04% |
| Osteonecrosis | 9 | 0.04% |
| Photopsia | 9 | 0.04% |
| Proctitis | 9 | 0.04% |
| Rash morbilliform | 9 | 0.04% |
| Sensory loss | 9 | 0.04% |
| Sinus bradycardia | 9 | 0.04% |
| Spinal cord compression | 9 | 0.04% |
| Thrombophlebitis | 9 | 0.04% |
| Thyroxine decreased | 9 | 0.04% |
| Tooth abscess | 9 | 0.04% |
| Vestibular disorder | 9 | 0.04% |
| Cytomegalovirus enterocolitis | 9 | 0.04% |
| Lipodystrophy acquired | 9 | 0.04% |
| Left ventricular dysfunction | 9 | 0.04% |
| Achromotrichia acquired | 9 | 0.04% |
| Pulmonary sepsis | 9 | 0.04% |
| Respiratory tract congestion | 9 | 0.04% |
| Autoimmune neutropenia | 9 | 0.04% |
| Secondary hypogonadism | 9 | 0.04% |
| Prostate cancer | 9 | 0.04% |
| Partial seizures | 9 | 0.04% |
| Autonomic neuropathy | 9 | 0.04% |
| Hepatic enzyme abnormal | 9 | 0.04% |
| Mixed liver injury | 9 | 0.04% |
| Posterior reversible encephalopathy syndrome | 9 | 0.04% |
| Spinal pain | 9 | 0.04% |
| Necrotising myositis | 9 | 0.04% |
| Immune-mediated endocrinopathy | 9 | 0.04% |
| Aphonia | 8 | 0.04% |
| Arteriosclerosis coronary artery | 8 | 0.04% |
| Biopsy | 8 | 0.04% |
| Bursitis | 8 | 0.04% |
| Cardiac pacemaker insertion | 8 | 0.04% |
| Cardiovascular disorder | 8 | 0.04% |
| Circulatory collapse | 8 | 0.04% |
| Cold sweat | 8 | 0.04% |
| Cushing's syndrome | 8 | 0.04% |
| Cyanosis | 8 | 0.04% |
| Dermatitis exfoliative generalised | 8 | 0.04% |
| Diverticulum intestinal | 8 | 0.04% |
| Dysaesthesia | 8 | 0.04% |
| Endocarditis | 8 | 0.04% |
| Faeces hard | 8 | 0.04% |
| Gastritis erosive | 8 | 0.04% |
| Glomerulonephritis | 8 | 0.04% |
| Hepatic cirrhosis | 8 | 0.04% |
| Hepatic necrosis | 8 | 0.04% |
| Hiatus hernia | 8 | 0.04% |
| Hyperkeratosis | 8 | 0.04% |
| Hypernatraemia | 8 | 0.04% |
| Hypogeusia | 8 | 0.04% |
| Lichen sclerosus | 8 | 0.04% |
| Localised infection | 8 | 0.04% |
| Malignant pleural effusion | 8 | 0.04% |
| Mental impairment | 8 | 0.04% |
| Neuromyopathy | 8 | 0.04% |
| Ototoxicity | 8 | 0.04% |
| Pain in jaw | 8 | 0.04% |
| Panic attack | 8 | 0.04% |
| Pathological fracture | 8 | 0.04% |
| Photophobia | 8 | 0.04% |
| Protein total decreased | 8 | 0.04% |
| Purpura | 8 | 0.04% |
| Renal cyst | 8 | 0.04% |
| Renal tubular acidosis | 8 | 0.04% |
| Restlessness | 8 | 0.04% |
| Scab | 8 | 0.04% |
| Sensory disturbance | 8 | 0.04% |
| Sunburn | 8 | 0.04% |
| Systemic lupus erythematosus | 8 | 0.04% |
| Tendonitis | 8 | 0.04% |
| Throat tightness | 8 | 0.04% |
| Thrombocytosis | 8 | 0.04% |
| Thrombotic thrombocytopenic purpura | 8 | 0.04% |
| Toothache | 8 | 0.04% |
| Multiple sclerosis relapse | 8 | 0.04% |
| Localised oedema | 8 | 0.04% |
| Neck mass | 8 | 0.04% |
| Transient acantholytic dermatosis | 8 | 0.04% |
| Small intestinal haemorrhage | 8 | 0.04% |
| Drug tolerance decreased | 8 | 0.04% |
| Nerve injury | 8 | 0.04% |
| Vanishing bile duct syndrome | 8 | 0.04% |
| Thyroxine free decreased | 8 | 0.04% |
| Thyroxine free increased | 8 | 0.04% |
| Paraesthesia oral | 8 | 0.04% |
| Infected neoplasm | 8 | 0.04% |
| Skin neoplasm excision | 8 | 0.04% |
| Blood alkaline phosphatase abnormal | 8 | 0.04% |
| Abdominal abscess | 8 | 0.04% |
| Cranial nerve disorder | 8 | 0.04% |
| Hormone level abnormal | 8 | 0.04% |
| Cytopenia | 8 | 0.04% |
| Latent autoimmune diabetes in adults | 8 | 0.04% |
| Acute polyneuropathy | 8 | 0.04% |
| Oral disorder | 8 | 0.04% |
| Cholestatic liver injury | 8 | 0.04% |
| Type 3 diabetes mellitus | 8 | 0.04% |
| Granulomatous lymphadenitis | 8 | 0.04% |
| Product storage error | 8 | 0.04% |
| Intestinal metastasis | 8 | 0.04% |
| Hypersensitivity pneumonitis | 8 | 0.04% |
| Oligoarthritis | 8 | 0.04% |
| Abscess | 7 | 0.03% |
| Acute pulmonary oedema | 7 | 0.03% |
| Alopecia areata | 7 | 0.03% |
| Anuria | 7 | 0.03% |
| Aortic dissection | 7 | 0.03% |
| Arterial thrombosis | 7 | 0.03% |
| Aspartate aminotransferase abnormal | 7 | 0.03% |
| Atypical pneumonia | 7 | 0.03% |
| Axonal neuropathy | 7 | 0.03% |
| Benign prostatic hyperplasia | 7 | 0.03% |
| Blepharitis | 7 | 0.03% |
| Breast pain | 7 | 0.03% |
| Bronchiolitis | 7 | 0.03% |
| Cerebral ischaemia | 7 | 0.03% |
| Coeliac disease | 7 | 0.03% |
| Colitis ischaemic | 7 | 0.03% |
| Decubitus ulcer | 7 | 0.03% |
| Eye infection | 7 | 0.03% |
| Furuncle | 7 | 0.03% |
| Gastritis haemorrhagic | 7 | 0.03% |
| Gingivitis | 7 | 0.03% |
| Glossitis | 7 | 0.03% |
| Blood urine present | 7 | 0.03% |
| Haemodialysis | 7 | 0.03% |
| Head discomfort | 7 | 0.03% |
| Hemiplegia | 7 | 0.03% |
| Herpes simplex | 7 | 0.03% |
| Hydrocephalus | 7 | 0.03% |
| Hypothermia | 7 | 0.03% |
| Inflammatory bowel disease | 7 | 0.03% |
| Iris adhesions | 7 | 0.03% |
| Kidney infection | 7 | 0.03% |
| Liver transplant rejection | 7 | 0.03% |
| Metastases to adrenals | 7 | 0.03% |
| Muscle injury | 7 | 0.03% |
| Obesity | 7 | 0.03% |
| Orbital myositis | 7 | 0.03% |
| Orchitis | 7 | 0.03% |
| Papillitis | 7 | 0.03% |
| Paraplegia | 7 | 0.03% |
| Peripheral coldness | 7 | 0.03% |
| Peripheral ischaemia | 7 | 0.03% |
| Phlebitis | 7 | 0.03% |
| Plasmapheresis | 7 | 0.03% |
| Platelet transfusion | 7 | 0.03% |
| Proctalgia | 7 | 0.03% |
| Pulmonary congestion | 7 | 0.03% |
| Pulmonary hypertension | 7 | 0.03% |
| Quadriplegia | 7 | 0.03% |
| Seborrhoeic keratosis | 7 | 0.03% |
| Skin erosion | 7 | 0.03% |
| Skin irritation | 7 | 0.03% |
| Sneezing | 7 | 0.03% |
| Spinal fracture | 7 | 0.03% |
| Tooth loss | 7 | 0.03% |
| Vestibular neuronitis | 7 | 0.03% |
| Wound secretion | 7 | 0.03% |
| Tooth infection | 7 | 0.03% |
| Deafness unilateral | 7 | 0.03% |
| Seasonal allergy | 7 | 0.03% |
| Lymphadenectomy | 7 | 0.03% |
| Post procedural haemorrhage | 7 | 0.03% |
| Cardiopulmonary failure | 7 | 0.03% |
| Chylothorax | 7 | 0.03% |
| Metastases to spleen | 7 | 0.03% |
| Tumour necrosis | 7 | 0.03% |
| Peripheral sensorimotor neuropathy | 7 | 0.03% |
| Pneumatosis intestinalis | 7 | 0.03% |
| Post procedural complication | 7 | 0.03% |
| Restless legs syndrome | 7 | 0.03% |
| Parophthalmia | 7 | 0.03% |
| Paraneoplastic syndrome | 7 | 0.03% |
| Limb injury | 7 | 0.03% |
| Neutrophil count abnormal | 7 | 0.03% |
| Psychotic disorder | 7 | 0.03% |
| Choroidal effusion | 7 | 0.03% |
| Infusion site extravasation | 7 | 0.03% |
| Procedural pain | 7 | 0.03% |
| Eastern Cooperative Oncology Group performance status worsened | 7 | 0.03% |
| Brain injury | 7 | 0.03% |
| Hospice care | 7 | 0.03% |
| Granulomatosis with polyangiitis | 7 | 0.03% |
| Product preparation error | 7 | 0.03% |
| Intracranial mass | 7 | 0.03% |
| Systemic scleroderma | 7 | 0.03% |
| Loss of personal independence in daily activities | 7 | 0.03% |
| Steroid diabetes | 7 | 0.03% |
| Spinal stenosis | 7 | 0.03% |
| Acute myeloid leukaemia | 6 | 0.03% |
| Ankle fracture | 6 | 0.03% |
| Aortitis | 6 | 0.03% |
| Atrioventricular block second degree | 6 | 0.03% |
| Autonomic nervous system imbalance | 6 | 0.03% |
| Blood creatine increased | 6 | 0.03% |
| Blood glucose decreased | 6 | 0.03% |
| Blood lactic acid increased | 6 | 0.03% |
| Blood magnesium decreased | 6 | 0.03% |
| Blood pressure systolic increased | 6 | 0.03% |
| Bone disorder | 6 | 0.03% |
| Campylobacter gastroenteritis | 6 | 0.03% |
| Cardiac failure acute | 6 | 0.03% |
| Cardiomegaly | 6 | 0.03% |
| Cerebellar syndrome | 6 | 0.03% |
| Clavicle fracture | 6 | 0.03% |
| Coronary artery occlusion | 6 | 0.03% |
| Cyst | 6 | 0.03% |
| Diabetic coma | 6 | 0.03% |
| Diabetic ketosis | 6 | 0.03% |
| Dry throat | 6 | 0.03% |
| Ecchymosis | 6 | 0.03% |
| Fibrin D dimer increased | 6 | 0.03% |
| Fracture | 6 | 0.03% |
| Full blood count decreased | 6 | 0.03% |
| Gallbladder disorder | 6 | 0.03% |
| Gastric cancer | 6 | 0.03% |
| Gastric haemorrhage | 6 | 0.03% |
| Gastroenteritis eosinophilic | 6 | 0.03% |
| Gastroenteritis viral | 6 | 0.03% |
| Gastrointestinal pain | 6 | 0.03% |
| Glycosylated haemoglobin increased | 6 | 0.03% |
| Haemoglobin abnormal | 6 | 0.03% |
| Heart rate abnormal | 6 | 0.03% |
| Heart rate decreased | 6 | 0.03% |
| Hernia | 6 | 0.03% |
| Humerus fracture | 6 | 0.03% |
| Hypoparathyroidism | 6 | 0.03% |
| Immunisation reaction | 6 | 0.03% |
| Large intestinal ulcer | 6 | 0.03% |
| Liver abscess | 6 | 0.03% |
| Lymphocyte count increased | 6 | 0.03% |
| Mood swings | 6 | 0.03% |
| Myelopathy | 6 | 0.03% |
| Myelosuppression | 6 | 0.03% |
| Myocardial fibrosis | 6 | 0.03% |
| Nail disorder | 6 | 0.03% |
| Necrosis | 6 | 0.03% |
| Oral mucosal blistering | 6 | 0.03% |
| Paraparesis | 6 | 0.03% |
| Platelet disorder | 6 | 0.03% |
| Polydipsia | 6 | 0.03% |
| Prurigo | 6 | 0.03% |
| Pseudomembranous colitis | 6 | 0.03% |
| Pulmonary thrombosis | 6 | 0.03% |
| Pulmonary tuberculosis | 6 | 0.03% |
| Recurrent cancer | 6 | 0.03% |
| Retinal artery occlusion | 6 | 0.03% |
| Retinitis | 6 | 0.03% |
| Rib fracture | 6 | 0.03% |
| Skin atrophy | 6 | 0.03% |
| Skin cancer | 6 | 0.03% |
| Small intestinal perforation | 6 | 0.03% |
| Spermatogenesis abnormal | 6 | 0.03% |
| Spinal compression fracture | 6 | 0.03% |
| Spinal osteoarthritis | 6 | 0.03% |
| Subarachnoid haemorrhage | 6 | 0.03% |
| Suicidal ideation | 6 | 0.03% |
| Wound dehiscence | 6 | 0.03% |
| Musculoskeletal disorder | 6 | 0.03% |
| Energy increased | 6 | 0.03% |
| Anal abscess | 6 | 0.03% |
| Emotional distress | 6 | 0.03% |
| Treatment noncompliance | 6 | 0.03% |
| Metastases to pancreas | 6 | 0.03% |
| Intervertebral disc protrusion | 6 | 0.03% |
| Cytomegalovirus test positive | 6 | 0.03% |
| Escherichia urinary tract infection | 6 | 0.03% |
| Large intestinal haemorrhage | 6 | 0.03% |
| Urinary tract infection bacterial | 6 | 0.03% |
| Thyroxine free abnormal | 6 | 0.03% |
| Congestive cardiomyopathy | 6 | 0.03% |
| Cutaneous lupus erythematosus | 6 | 0.03% |
| Skin laceration | 6 | 0.03% |
| Enterocolitis infectious | 6 | 0.03% |
| Intestinal haemorrhage | 6 | 0.03% |
| Arthritis infective | 6 | 0.03% |
| Pseudomonas infection | 6 | 0.03% |
| Renal injury | 6 | 0.03% |
| Renal neoplasm | 6 | 0.03% |
| Parkinson's disease | 6 | 0.03% |
| Obstructive airways disorder | 6 | 0.03% |
| Soft tissue infection | 6 | 0.03% |
| Bowel movement irregularity | 6 | 0.03% |
| Circumstance or information capable of leading to medication error | 6 | 0.03% |
| Oral mucosa erosion | 6 | 0.03% |
| Pregnancy on contraceptive | 6 | 0.03% |
| Overlap syndrome | 6 | 0.03% |
| Clostridium test positive | 6 | 0.03% |
| Maternal exposure before pregnancy | 6 | 0.03% |
| Aspergillus infection | 6 | 0.03% |
| Pulmonary pain | 6 | 0.03% |
| Noninfective encephalitis | 6 | 0.03% |
| Limb mass | 6 | 0.03% |
| Autoimmune endocrine disorder | 6 | 0.03% |
| Silent thyroiditis | 6 | 0.03% |
| Autoimmune lung disease | 6 | 0.03% |
| Product prescribing error | 6 | 0.03% |
| Central nervous system vasculitis | 6 | 0.03% |
| Large intestine infection | 6 | 0.03% |
| Immune-mediated hyperthyroidism | 6 | 0.03% |
| Biliary obstruction | 6 | 0.03% |
| SARS-CoV-2 test positive | 6 | 0.03% |
| Accidental overdose | 5 | 0.02% |
| Actinic keratosis | 5 | 0.02% |
| Activated partial thromboplastin time prolonged | 5 | 0.02% |
| Aggression | 5 | 0.02% |
| Anal fissure | 5 | 0.02% |
| Antiphospholipid syndrome | 5 | 0.02% |
| Arteriosclerosis | 5 | 0.02% |
| Arthropod bite | 5 | 0.02% |
| Back disorder | 5 | 0.02% |
| Bile duct stone | 5 | 0.02% |
| Bladder cancer | 5 | 0.02% |
| Blood calcium decreased | 5 | 0.02% |
| Blood calcium increased | 5 | 0.02% |
| Blood chloride decreased | 5 | 0.02% |
| Blood creatinine abnormal | 5 | 0.02% |
| Bronchospasm | 5 | 0.02% |
| Bundle branch block right | 5 | 0.02% |
| Colostomy | 5 | 0.02% |
| Conjunctival oedema | 5 | 0.02% |
| Coordination abnormal | 5 | 0.02% |
| Diaphragm muscle weakness | 5 | 0.02% |
| Dizziness postural | 5 | 0.02% |
| Ear disorder | 5 | 0.02% |
| Ear infection | 5 | 0.02% |
| Escherichia sepsis | 5 | 0.02% |
| Eye discharge | 5 | 0.02% |
| Eyelid oedema | 5 | 0.02% |
| Facial pain | 5 | 0.02% |
| Fear | 5 | 0.02% |
| Fistula | 5 | 0.02% |
| Food poisoning | 5 | 0.02% |
| Gingival bleeding | 5 | 0.02% |
| Gingival pain | 5 | 0.02% |
| Glomerular filtration rate decreased | 5 | 0.02% |
| Granulomatous liver disease | 5 | 0.02% |
| Haemorrhagic stroke | 5 | 0.02% |
| Hepatitis toxic | 5 | 0.02% |
| Hepatosplenomegaly | 5 | 0.02% |
| Herpes virus infection | 5 | 0.02% |
| Hypokinesia | 5 | 0.02% |
| IgA nephropathy | 5 | 0.02% |
| IIIrd nerve paralysis | 5 | 0.02% |
| Incoherent | 5 | 0.02% |
| Inguinal hernia | 5 | 0.02% |
| Intestinal ischaemia | 5 | 0.02% |
| Intestinal pseudo-obstruction | 5 | 0.02% |
| Laryngitis | 5 | 0.02% |
| Lichenification | 5 | 0.02% |
| Lung consolidation | 5 | 0.02% |
| Lymphangitis | 5 | 0.02% |
| Lymphocyte count abnormal | 5 | 0.02% |
| Maculopathy | 5 | 0.02% |
| Mania | 5 | 0.02% |
| Mastication disorder | 5 | 0.02% |
| Melanosis | 5 | 0.02% |
| Meningism | 5 | 0.02% |
| Metastases to abdominal cavity | 5 | 0.02% |
| Metastases to kidney | 5 | 0.02% |
| Mucosal dryness | 5 | 0.02% |
| Mucous stools | 5 | 0.02% |
| Myoclonus | 5 | 0.02% |
| Necrotising fasciitis | 5 | 0.02% |
| Neurodermatitis | 5 | 0.02% |
| Neurogenic bladder | 5 | 0.02% |
| Nystagmus | 5 | 0.02% |
| Optic ischaemic neuropathy | 5 | 0.02% |
| Pancreatic carcinoma | 5 | 0.02% |
| Pemphigus | 5 | 0.02% |
| Periodontitis | 5 | 0.02% |
| Periorbital oedema | 5 | 0.02% |
| Personality change | 5 | 0.02% |
| Pneumonia klebsiella | 5 | 0.02% |
| Postoperative wound infection | 5 | 0.02% |
| Pulmonary alveolar haemorrhage | 5 | 0.02% |
| Pulmonary haemorrhage | 5 | 0.02% |
| Respiration abnormal | 5 | 0.02% |
| Retinal oedema | 5 | 0.02% |
| Retinal vasculitis | 5 | 0.02% |
| Retinal vein occlusion | 5 | 0.02% |
| Rhinitis | 5 | 0.02% |
| Road traffic accident | 5 | 0.02% |
| Scleroderma-like reaction | 5 | 0.02% |
| Scratch | 5 | 0.02% |
| Seroma | 5 | 0.02% |
| Skin fissures | 5 | 0.02% |
| Swollen tongue | 5 | 0.02% |
| Synovial cyst | 5 | 0.02% |
| Tenderness | 5 | 0.02% |
| Thinking abnormal | 5 | 0.02% |
| Thyroxine increased | 5 | 0.02% |
| Tongue discolouration | 5 | 0.02% |
| Tri-iodothyronine decreased | 5 | 0.02% |
| Unresponsive to stimuli | 5 | 0.02% |
| Vitamin D deficiency | 5 | 0.02% |
| Vitritis | 5 | 0.02% |
| Stent placement | 5 | 0.02% |
| Miller Fisher syndrome | 5 | 0.02% |
| Lumbar vertebral fracture | 5 | 0.02% |
| Muscle strain | 5 | 0.02% |
| Metastases to chest wall | 5 | 0.02% |
| Coronavirus infection | 5 | 0.02% |
| Eye pruritus | 5 | 0.02% |
| Primary adrenal insufficiency | 5 | 0.02% |
| Wound complication | 5 | 0.02% |
| Acquired haemophilia | 5 | 0.02% |
| Depressive symptom | 5 | 0.02% |
| Hepatic infection | 5 | 0.02% |
| Gastrointestinal oedema | 5 | 0.02% |
| Thyroid mass | 5 | 0.02% |
| Urine output decreased | 5 | 0.02% |
| Endocrine ophthalmopathy | 5 | 0.02% |
| Joint injury | 5 | 0.02% |
| Abdominal neoplasm | 5 | 0.02% |
| Dialysis | 5 | 0.02% |
| Intervertebral disc degeneration | 5 | 0.02% |
| Intra-abdominal haemorrhage | 5 | 0.02% |
| Ischaemia | 5 | 0.02% |
| Meningeal disorder | 5 | 0.02% |
| Metastatic neoplasm | 5 | 0.02% |
| Sudden hearing loss | 5 | 0.02% |
| White blood cell disorder | 5 | 0.02% |
| Disease recurrence | 5 | 0.02% |
| Food intolerance | 5 | 0.02% |
| Intestinal operation | 5 | 0.02% |
| Lymphocytic infiltration | 5 | 0.02% |
| Vitamin D decreased | 5 | 0.02% |
| Transaminases abnormal | 5 | 0.02% |
| Thyroid hormones increased | 5 | 0.02% |
| Cataract operation | 5 | 0.02% |
| Osteonecrosis of jaw | 5 | 0.02% |
| Functional gastrointestinal disorder | 5 | 0.02% |
| Central nervous system haemorrhage | 5 | 0.02% |
| Dropped head syndrome | 5 | 0.02% |
| Cancer surgery | 5 | 0.02% |
| Myocardial necrosis marker increased | 5 | 0.02% |
| Pancreatic toxicity | 5 | 0.02% |
| Ulcerative gastritis | 5 | 0.02% |
| Perforation | 5 | 0.02% |
| Incorrect product administration duration | 5 | 0.02% |
| Lung opacity | 5 | 0.02% |
| Blood loss anaemia | 5 | 0.02% |
| Immune-mediated encephalopathy | 5 | 0.02% |
| Warm autoimmune haemolytic anaemia | 5 | 0.02% |
| Superficial inflammatory dermatosis | 5 | 0.02% |
| Abdominal mass | 4 | 0.02% |
| Abnormal loss of weight | 4 | 0.02% |
| Abortion spontaneous | 4 | 0.02% |
| Alopecia universalis | 4 | 0.02% |
| Aneurysm | 4 | 0.02% |
| Anger | 4 | 0.02% |
| Anosmia | 4 | 0.02% |
| Apathy | 4 | 0.02% |
| Asterixis | 4 | 0.02% |
| Azotaemia | 4 | 0.02% |
| Back injury | 4 | 0.02% |
| Bilirubin conjugated increased | 4 | 0.02% |
| Blindness transient | 4 | 0.02% |
| Blood lactate dehydrogenase abnormal | 4 | 0.02% |
| Blood lactate dehydrogenase decreased | 4 | 0.02% |
| Blood potassium abnormal | 4 | 0.02% |
| Blood urea decreased | 4 | 0.02% |
| Blood uric acid increased | 4 | 0.02% |
| Bone cancer | 4 | 0.02% |
| Bronchiectasis | 4 | 0.02% |
| Cholangitis acute | 4 | 0.02% |
| Cholecystectomy | 4 | 0.02% |
| Choroiditis | 4 | 0.02% |
| Chronic sinusitis | 4 | 0.02% |
| Corneal perforation | 4 | 0.02% |
| Cortisol abnormal | 4 | 0.02% |
| Creatinine renal clearance increased | 4 | 0.02% |
| Decreased activity | 4 | 0.02% |
| Dermatitis allergic | 4 | 0.02% |
| Dermatitis contact | 4 | 0.02% |
| Embolism venous | 4 | 0.02% |
| Endophthalmitis | 4 | 0.02% |
| Epididymitis | 4 | 0.02% |
| Episcleritis | 4 | 0.02% |
| Exophthalmos | 4 | 0.02% |
| Extrasystoles | 4 | 0.02% |
| Facial bones fracture | 4 | 0.02% |
| Folliculitis | 4 | 0.02% |
| Fungal skin infection | 4 | 0.02% |
| Gastrointestinal carcinoma | 4 | 0.02% |
| Gouty arthritis | 4 | 0.02% |
| Gravitational oedema | 4 | 0.02% |
| Haemangioma | 4 | 0.02% |
| Haematocrit increased | 4 | 0.02% |
| Haemorrhoids thrombosed | 4 | 0.02% |
| Hair growth abnormal | 4 | 0.02% |
| Hair texture abnormal | 4 | 0.02% |
| Hallucination, visual | 4 | 0.02% |
| Heart rate irregular | 4 | 0.02% |
| Hepatic cyst | 4 | 0.02% |
| Hepatorenal failure | 4 | 0.02% |
| Hiccups | 4 | 0.02% |
| Hilar lymphadenopathy | 4 | 0.02% |
| Hyperprolactinaemia | 4 | 0.02% |
| Hypertensive crisis | 4 | 0.02% |
| Hypochromic anaemia | 4 | 0.02% |
| Hypotonia | 4 | 0.02% |
| Intraocular pressure increased | 4 | 0.02% |
| Knee arthroplasty | 4 | 0.02% |
| Libido decreased | 4 | 0.02% |
| Lipoma | 4 | 0.02% |
| Listless | 4 | 0.02% |
| Lymph node pain | 4 | 0.02% |
| Lymphocytosis | 4 | 0.02% |
| Meningitis viral | 4 | 0.02% |
| Miliaria | 4 | 0.02% |
| Mitral valve incompetence | 4 | 0.02% |
| Monoplegia | 4 | 0.02% |
| Muscle necrosis | 4 | 0.02% |
| Myocardial ischaemia | 4 | 0.02% |
| Nerve compression | 4 | 0.02% |
| Nocturia | 4 | 0.02% |
| Normocytic anaemia | 4 | 0.02% |
| Obstruction gastric | 4 | 0.02% |
| Odynophagia | 4 | 0.02% |
| Oesophageal achalasia | 4 | 0.02% |
| Oesophageal ulcer | 4 | 0.02% |
| Osteolysis | 4 | 0.02% |
| Osteomyelitis | 4 | 0.02% |
| Osteoporotic fracture | 4 | 0.02% |
| Paronychia | 4 | 0.02% |
| Parotid gland enlargement | 4 | 0.02% |
| Parotitis | 4 | 0.02% |
| Pelvic pain | 4 | 0.02% |
| Petit mal epilepsy | 4 | 0.02% |
| Pharyngitis | 4 | 0.02% |
| Pleuritic pain | 4 | 0.02% |
| Polyglandular disorder | 4 | 0.02% |
| Progressive multifocal leukoencephalopathy | 4 | 0.02% |
| Prostatitis | 4 | 0.02% |
| Protein total increased | 4 | 0.02% |
| Pseudolymphoma | 4 | 0.02% |
| Pulmonary artery thrombosis | 4 | 0.02% |
| Pulse abnormal | 4 | 0.02% |
| Rales | 4 | 0.02% |
| Respiratory depression | 4 | 0.02% |
| Retinal disorder | 4 | 0.02% |
| Retinal haemorrhage | 4 | 0.02% |
| Retroperitoneal haemorrhage | 4 | 0.02% |
| Rosacea | 4 | 0.02% |
| Sarcoma | 4 | 0.02% |
| Sluggishness | 4 | 0.02% |
| Subcutaneous abscess | 4 | 0.02% |
| Superinfection | 4 | 0.02% |
| Tachypnoea | 4 | 0.02% |
| Tendon rupture | 4 | 0.02% |
| Thrombocytopenic purpura | 4 | 0.02% |
| Thyroiditis acute | 4 | 0.02% |
| Tonsillitis | 4 | 0.02% |
| Urinary tract disorder | 4 | 0.02% |
| Vaginal haemorrhage | 4 | 0.02% |
| Vasculitis necrotising | 4 | 0.02% |
| Ventricular arrhythmia | 4 | 0.02% |
| Viral upper respiratory tract infection | 4 | 0.02% |
| Vitreous detachment | 4 | 0.02% |
| Vocal cord paralysis | 4 | 0.02% |
| Vulvovaginal candidiasis | 4 | 0.02% |
| Wrist fracture | 4 | 0.02% |
| Xerosis | 4 | 0.02% |
| Pneumoperitoneum | 4 | 0.02% |
| Tumour marker increased | 4 | 0.02% |
| Lymphocele | 4 | 0.02% |
| Bedridden | 4 | 0.02% |
| Axillary mass | 4 | 0.02% |
| Neutropenic sepsis | 4 | 0.02% |
| Ocular myasthenia | 4 | 0.02% |
| Sudden cardiac death | 4 | 0.02% |
| Tachyarrhythmia | 4 | 0.02% |
| Anal haemorrhage | 4 | 0.02% |
| Quadriparesis | 4 | 0.02% |
| Pituitary haemorrhage | 4 | 0.02% |
| Muscle tightness | 4 | 0.02% |
| Groin abscess | 4 | 0.02% |
| Tumour ulceration | 4 | 0.02% |
| Subileus | 4 | 0.02% |
| Lupus-like syndrome | 4 | 0.02% |
| Staphylococcal bacteraemia | 4 | 0.02% |
| Bile duct stenosis | 4 | 0.02% |
| Physical deconditioning | 4 | 0.02% |
| Large intestine polyp | 4 | 0.02% |
| Metastases to peritoneum | 4 | 0.02% |
| Adrenomegaly | 4 | 0.02% |
| Tumour associated fever | 4 | 0.02% |
| Lymphatic disorder | 4 | 0.02% |
| Lymphangiosis carcinomatosa | 4 | 0.02% |
| Inguinal mass | 4 | 0.02% |
| Adrenal mass | 4 | 0.02% |
| Central venous catheterisation | 4 | 0.02% |
| Arthritis bacterial | 4 | 0.02% |
| Thermal burn | 4 | 0.02% |
| Extraocular muscle disorder | 4 | 0.02% |
| Bacterial sepsis | 4 | 0.02% |
| Escherichia bacteraemia | 4 | 0.02% |
| Disorder of orbit | 4 | 0.02% |
| Temperature intolerance | 4 | 0.02% |
| Hepatic mass | 4 | 0.02% |
| Metabolic disorder | 4 | 0.02% |
| Serositis | 4 | 0.02% |
| Hepatitis B reactivation | 4 | 0.02% |
| Infrequent bowel movements | 4 | 0.02% |
| Pleuropericarditis | 4 | 0.02% |
| Abdominal cavity drainage | 4 | 0.02% |
| Diffuse alveolar damage | 4 | 0.02% |
| Biliary tract disorder | 4 | 0.02% |
| Bladder disorder | 4 | 0.02% |
| Escherichia infection | 4 | 0.02% |
| Eye movement disorder | 4 | 0.02% |
| Female genital tract fistula | 4 | 0.02% |
| Pelvic fracture | 4 | 0.02% |
| Oesophageal disorder | 4 | 0.02% |
| Optic nerve disorder | 4 | 0.02% |
| Oral fungal infection | 4 | 0.02% |
| Spondylitis | 4 | 0.02% |
| Upper limb fracture | 4 | 0.02% |
| Venous thrombosis limb | 4 | 0.02% |
| Gastrointestinal ulcer | 4 | 0.02% |
| Erectile dysfunction | 4 | 0.02% |
| Psychiatric symptom | 4 | 0.02% |
| Polyp | 4 | 0.02% |
| Immunodeficiency | 4 | 0.02% |
| Abdominal operation | 4 | 0.02% |
| Bone lesion | 4 | 0.02% |
| Diverticular perforation | 4 | 0.02% |
| Eye operation | 4 | 0.02% |
| Immunosuppression | 4 | 0.02% |
| Lung operation | 4 | 0.02% |
| Large intestinal obstruction | 4 | 0.02% |
| Metastases to gastrointestinal tract | 4 | 0.02% |
| Sinus disorder | 4 | 0.02% |
| Arterial occlusive disease | 4 | 0.02% |
| Hyperglycaemic hyperosmolar nonketotic syndrome | 4 | 0.02% |
| Solar lentigo | 4 | 0.02% |
| Eosinophilic oesophagitis | 4 | 0.02% |
| Paraneoplastic retinopathy | 4 | 0.02% |
| Aortic arteriosclerosis | 4 | 0.02% |
| Ocular sarcoidosis | 4 | 0.02% |
| Acute interstitial pneumonitis | 4 | 0.02% |
| Post procedural infection | 4 | 0.02% |
| Quality of life decreased | 4 | 0.02% |
| Hyperlipasaemia | 4 | 0.02% |
| Interstitial granulomatous dermatitis | 4 | 0.02% |
| Creatinine renal clearance abnormal | 4 | 0.02% |
| Inflammatory marker increased | 4 | 0.02% |
| Staphylococcus test positive | 4 | 0.02% |
| Macular fibrosis | 4 | 0.02% |
| Hepatic cancer | 4 | 0.02% |
| Intentional product misuse | 4 | 0.02% |
| Body temperature abnormal | 4 | 0.02% |
| Autoimmune encephalopathy | 4 | 0.02% |
| Wrong technique in product usage process | 4 | 0.02% |
| Frustration tolerance decreased | 4 | 0.02% |
| Product supply issue | 4 | 0.02% |
| Ureterolithiasis | 4 | 0.02% |
| Eosinophilic granulomatosis with polyangiitis | 4 | 0.02% |
| Hyperleukocytosis | 4 | 0.02% |
| Immune-mediated neuropathy | 4 | 0.02% |
| Autoimmune thyroid disorder | 4 | 0.02% |
| Tumour hyperprogression | 4 | 0.02% |
| Product administered to patient of inappropriate age | 4 | 0.02% |
| Immune-mediated cholangitis | 4 | 0.02% |
| Immune-mediated cystitis | 4 | 0.02% |
| Abdominal rigidity | 3 | 0.01% |
| Abdominal tenderness | 3 | 0.01% |
| Abortion induced | 3 | 0.01% |
| Abscess intestinal | 3 | 0.01% |
| Adenocarcinoma | 3 | 0.01% |
| Affective disorder | 3 | 0.01% |
| Alanine aminotransferase decreased | 3 | 0.01% |
| Amaurosis | 3 | 0.01% |
| Amyloidosis | 3 | 0.01% |
| Anal fistula | 3 | 0.01% |
| Angina unstable | 3 | 0.01% |
| Angle closure glaucoma | 3 | 0.01% |
| Aptyalism | 3 | 0.01% |
| Arrhythmia supraventricular | 3 | 0.01% |
| Aspiration pleural cavity | 3 | 0.01% |
| Asthenopia | 3 | 0.01% |
| Behaviour disorder | 3 | 0.01% |
| Bell's palsy | 3 | 0.01% |
| Biliary colic | 3 | 0.01% |
| Blepharospasm | 3 | 0.01% |
| Blood cholesterol increased | 3 | 0.01% |
| Blood corticotrophin abnormal | 3 | 0.01% |
| Blood corticotrophin increased | 3 | 0.01% |
| Blood fibrinogen decreased | 3 | 0.01% |
| Blood sodium abnormal | 3 | 0.01% |
| Blood sodium increased | 3 | 0.01% |
| Blood triglycerides increased | 3 | 0.01% |
| Brain abscess | 3 | 0.01% |
| Brain herniation | 3 | 0.01% |
| Brain neoplasm malignant | 3 | 0.01% |
| Breast mass | 3 | 0.01% |
| Bronchitis chronic | 3 | 0.01% |
| Bulbar palsy | 3 | 0.01% |
| Cardiac failure chronic | 3 | 0.01% |
| Cauda equina syndrome | 3 | 0.01% |
| Cerebral atrophy | 3 | 0.01% |
| Cerebral thrombosis | 3 | 0.01% |
| Cerebral venous thrombosis | 3 | 0.01% |
| Chest X-ray abnormal | 3 | 0.01% |
| Choking | 3 | 0.01% |
| Coma hepatic | 3 | 0.01% |
| Completed suicide | 3 | 0.01% |
| Concomitant disease aggravated | 3 | 0.01% |
| Coombs positive haemolytic anaemia | 3 | 0.01% |
| Coronary artery stenosis | 3 | 0.01% |
| Crying | 3 | 0.01% |
| Cryoglobulinaemia | 3 | 0.01% |
| CSF lymphocyte count increased | 3 | 0.01% |
| Cushingoid | 3 | 0.01% |
| Cutaneous vasculitis | 3 | 0.01% |
| Defaecation urgency | 3 | 0.01% |
| Delusion | 3 | 0.01% |
| Dementia Alzheimer's type | 3 | 0.01% |
| Dental caries | 3 | 0.01% |
| Diabetic hyperglycaemic coma | 3 | 0.01% |
| Dyslalia | 3 | 0.01% |
| Electrocardiogram abnormal | 3 | 0.01% |
| Embolic stroke | 3 | 0.01% |
| Emphysema | 3 | 0.01% |
| Encephalomyelitis | 3 | 0.01% |
| Epiglottitis | 3 | 0.01% |
| Femoral neck fracture | 3 | 0.01% |
| Fibrosis | 3 | 0.01% |
| Foot fracture | 3 | 0.01% |
| Formication | 3 | 0.01% |
| Full blood count abnormal | 3 | 0.01% |
| Fungaemia | 3 | 0.01% |
| Gastric ulcer haemorrhage | 3 | 0.01% |
| Genital rash | 3 | 0.01% |
| Gingival disorder | 3 | 0.01% |
| Glomerulonephritis rapidly progressive | 3 | 0.01% |
| Granuloma skin | 3 | 0.01% |
| Haemoglobin increased | 3 | 0.01% |
| Haemolytic uraemic syndrome | 3 | 0.01% |
| Hepatic neoplasm | 3 | 0.01% |
| Hepatitis E | 3 | 0.01% |
| Hepatorenal syndrome | 3 | 0.01% |
| Hunger | 3 | 0.01% |
| Hypermagnesaemia | 3 | 0.01% |
| Hyperpituitarism | 3 | 0.01% |
| Hypertriglyceridaemia | 3 | 0.01% |
| Hypoproteinaemia | 3 | 0.01% |
| Hypotony of eye | 3 | 0.01% |
| Hypovolaemic shock | 3 | 0.01% |
| Immobile | 3 | 0.01% |
| Intraocular pressure decreased | 3 | 0.01% |
| Irritable bowel syndrome | 3 | 0.01% |
| Ischaemic hepatitis | 3 | 0.01% |
| Jejunal perforation | 3 | 0.01% |
| Joint dislocation | 3 | 0.01% |
| Laparotomy | 3 | 0.01% |
| Laryngeal oedema | 3 | 0.01% |
| Leukaemia | 3 | 0.01% |
| Lipase decreased | 3 | 0.01% |
| Lyme disease | 3 | 0.01% |
| Malignant ascites | 3 | 0.01% |
| Mastitis | 3 | 0.01% |
| Medication error | 3 | 0.01% |
| Meningioma | 3 | 0.01% |
| Meningitis bacterial | 3 | 0.01% |
| Mesenteric vein thrombosis | 3 | 0.01% |
| Microcytic anaemia | 3 | 0.01% |
| Micturition disorder | 3 | 0.01% |
| Monoparesis | 3 | 0.01% |
| Motor neurone disease | 3 | 0.01% |
| Mouth haemorrhage | 3 | 0.01% |
| Muscle rupture | 3 | 0.01% |
| Mydriasis | 3 | 0.01% |
| Nervousness | 3 | 0.01% |
| Neutrophilia | 3 | 0.01% |
| Nightmare | 3 | 0.01% |
| Ocular hypertension | 3 | 0.01% |
| Oedema mucosal | 3 | 0.01% |
| Oesophageal stenosis | 3 | 0.01% |
| Oliguria | 3 | 0.01% |
| Optic atrophy | 3 | 0.01% |
| Oral discomfort | 3 | 0.01% |
| Oral lichen planus | 3 | 0.01% |
| Osteitis | 3 | 0.01% |
| Osteochondrosis | 3 | 0.01% |
| Otitis media | 3 | 0.01% |
| Ovarian cancer | 3 | 0.01% |
| Ovarian cyst | 3 | 0.01% |
| Painful respiration | 3 | 0.01% |
| Pancreatic cyst | 3 | 0.01% |
| Papillary thyroid cancer | 3 | 0.01% |
| Parkinsonism | 3 | 0.01% |
| Peroneal nerve palsy | 3 | 0.01% |
| Petechiae | 3 | 0.01% |
| Pharyngeal oedema | 3 | 0.01% |
| Plasma cell myeloma | 3 | 0.01% |
| Platelet count abnormal | 3 | 0.01% |
| Pneumonia cytomegaloviral | 3 | 0.01% |
| Post-traumatic stress disorder | 3 | 0.01% |
| Prostatic specific antigen increased | 3 | 0.01% |
| Psychomotor hyperactivity | 3 | 0.01% |
| Punctate keratitis | 3 | 0.01% |
| Pustule | 3 | 0.01% |
| Pyoderma gangrenosum | 3 | 0.01% |
| Radiation associated pain | 3 | 0.01% |
| Radiation injury | 3 | 0.01% |
| Rectal cancer | 3 | 0.01% |
| Red blood cell count increased | 3 | 0.01% |
| Renal pain | 3 | 0.01% |
| Renal vasculitis | 3 | 0.01% |
| Retrograde amnesia | 3 | 0.01% |
| Rhabdomyosarcoma | 3 | 0.01% |
| Schizophrenia | 3 | 0.01% |
| Skin necrosis | 3 | 0.01% |
| Skin papilloma | 3 | 0.01% |
| Sleep apnoea syndrome | 3 | 0.01% |
| Solar dermatitis | 3 | 0.01% |
| Spleen disorder | 3 | 0.01% |
| Splenic infarction | 3 | 0.01% |
| Suicide attempt | 3 | 0.01% |
| T-cell lymphoma | 3 | 0.01% |
| Tendon disorder | 3 | 0.01% |
| Testicular pain | 3 | 0.01% |
| Tinea pedis | 3 | 0.01% |
| Tongue coated | 3 | 0.01% |
| Tongue ulceration | 3 | 0.01% |
| Tooth injury | 3 | 0.01% |
| Toxic encephalopathy | 3 | 0.01% |
| Tracheobronchitis | 3 | 0.01% |
| Tuberculosis | 3 | 0.01% |
| Tumour flare | 3 | 0.01% |
| Umbilical hernia | 3 | 0.01% |
| Urinary hesitation | 3 | 0.01% |
| Vascular injury | 3 | 0.01% |
| Ventricular extrasystoles | 3 | 0.01% |
| Visual field defect | 3 | 0.01% |
| Volvulus | 3 | 0.01% |
| Wheelchair user | 3 | 0.01% |
| Xerophthalmia | 3 | 0.01% |
| Yellow skin | 3 | 0.01% |
| Fibromyalgia | 3 | 0.01% |
| Lip disorder | 3 | 0.01% |
| Terminal state | 3 | 0.01% |
| Joint range of motion decreased | 3 | 0.01% |
| Acute generalised exanthematous pustulosis | 3 | 0.01% |
| Streptococcal sepsis | 3 | 0.01% |
| Cytomegalovirus gastritis | 3 | 0.01% |
| Coma acidotic | 3 | 0.01% |
| Chapped lips | 3 | 0.01% |
| Oesophagitis ulcerative | 3 | 0.01% |
| Intestinal villi atrophy | 3 | 0.01% |
| Blood phosphorus decreased | 3 | 0.01% |
| Impaired driving ability | 3 | 0.01% |
| Metastases to heart | 3 | 0.01% |
| Blood glucose fluctuation | 3 | 0.01% |
| Blood bilirubin decreased | 3 | 0.01% |
| Pneumomediastinum | 3 | 0.01% |
| Blood phosphorus increased | 3 | 0.01% |
| Abscess limb | 3 | 0.01% |
| Skin tightness | 3 | 0.01% |
| Cytokine storm | 3 | 0.01% |
| Oral dysaesthesia | 3 | 0.01% |
| Lacunar infarction | 3 | 0.01% |
| Wound haemorrhage | 3 | 0.01% |
| Prostatomegaly | 3 | 0.01% |
| Conjunctival hyperaemia | 3 | 0.01% |
| Ocular discomfort | 3 | 0.01% |
| Neutrophil percentage increased | 3 | 0.01% |
| Lymphocyte percentage decreased | 3 | 0.01% |
| Impaired work ability | 3 | 0.01% |
| Gastrointestinal tube insertion | 3 | 0.01% |
| Organ failure | 3 | 0.01% |
| Polypectomy | 3 | 0.01% |
| Type IV hypersensitivity reaction | 3 | 0.01% |
| Keratic precipitates | 3 | 0.01% |
| Brain tumour operation | 3 | 0.01% |
| Thoracic cavity drainage | 3 | 0.01% |
| Skin burning sensation | 3 | 0.01% |
| Mean platelet volume increased | 3 | 0.01% |
| Brain cancer metastatic | 3 | 0.01% |
| Abdominal infection | 3 | 0.01% |
| Catheter site infection | 3 | 0.01% |
| Periorbital swelling | 3 | 0.01% |
| Weight abnormal | 3 | 0.01% |
| Biliary dilatation | 3 | 0.01% |
| Therapeutic embolisation | 3 | 0.01% |
| Hypoaesthesia oral | 3 | 0.01% |
| Paranasal sinus hypersecretion | 3 | 0.01% |
| Cytoreductive surgery | 3 | 0.01% |
| Major depression | 3 | 0.01% |
| Dyslipidaemia | 3 | 0.01% |
| Ocular icterus | 3 | 0.01% |
| Retroperitoneal haematoma | 3 | 0.01% |
| Blood bilirubin abnormal | 3 | 0.01% |
| Cytomegalovirus viraemia | 3 | 0.01% |
| Extremity necrosis | 3 | 0.01% |
| Wound infection staphylococcal | 3 | 0.01% |
| Palliative care | 3 | 0.01% |
| Adhesion | 3 | 0.01% |
| Urine output increased | 3 | 0.01% |
| Anti-thyroid antibody positive | 3 | 0.01% |
| Transplant failure | 3 | 0.01% |
| Appendix disorder | 3 | 0.01% |
| Blood electrolytes abnormal | 3 | 0.01% |
| Clostridial infection | 3 | 0.01% |
| Connective tissue disorder | 3 | 0.01% |
| Electrocardiogram change | 3 | 0.01% |
| Eyelid disorder | 3 | 0.01% |
| Genital haemorrhage | 3 | 0.01% |
| Infarction | 3 | 0.01% |
| Lymphoproliferative disorder | 3 | 0.01% |
| Mucosal erosion | 3 | 0.01% |
| Pneumonia fungal | 3 | 0.01% |
| Spinal disorder | 3 | 0.01% |
| Renal function test abnormal | 3 | 0.01% |
| Rhinovirus infection | 3 | 0.01% |
| Intervertebral disc disorder | 3 | 0.01% |
| Urinary tract obstruction | 3 | 0.01% |
| Respiratory syncytial virus infection | 3 | 0.01% |
| Bone marrow disorder | 3 | 0.01% |
| Brain operation | 3 | 0.01% |
| Chorioretinal disorder | 3 | 0.01% |
| Fat necrosis | 3 | 0.01% |
| Gamma radiation therapy | 3 | 0.01% |
| Tooth extraction | 3 | 0.01% |
| Vascular access complication | 3 | 0.01% |
| Microangiopathy | 3 | 0.01% |
| Skin graft | 3 | 0.01% |
| Heparin-induced thrombocytopenia | 3 | 0.01% |
| Grip strength decreased | 3 | 0.01% |
| Endocrine toxicity | 3 | 0.01% |
| Mesenteric panniculitis | 3 | 0.01% |
| Cystitis noninfective | 3 | 0.01% |
| Gene mutation identification test positive | 3 | 0.01% |
| Incorrect drug administration rate | 3 | 0.01% |
| Breath sounds abnormal | 3 | 0.01% |
| Vulvovaginal mycotic infection | 3 | 0.01% |
| Pulmonary arterial hypertension | 3 | 0.01% |
| Phrenic nerve paralysis | 3 | 0.01% |
| Mitochondrial myopathy acquired | 3 | 0.01% |
| Therapy cessation | 3 | 0.01% |
| Loose tooth | 3 | 0.01% |
| Pharyngeal inflammation | 3 | 0.01% |
| Mucosal infection | 3 | 0.01% |
| Central nervous system necrosis | 3 | 0.01% |
| Autoimmune inner ear disease | 3 | 0.01% |
| Tendon pain | 3 | 0.01% |
| Thyroid cancer | 3 | 0.01% |
| Mechanical ventilation | 3 | 0.01% |
| Skin plaque | 3 | 0.01% |
| Oncologic complication | 3 | 0.01% |
| Oropharyngeal discomfort | 3 | 0.01% |
| C-reactive protein abnormal | 3 | 0.01% |
| Respiratory tract inflammation | 3 | 0.01% |
| Septic encephalopathy | 3 | 0.01% |
| Product quality issue | 3 | 0.01% |
| Subretinal fluid | 3 | 0.01% |
| Autoimmune pancytopenia | 3 | 0.01% |
| Device related sepsis | 3 | 0.01% |
| Pseudomonas test positive | 3 | 0.01% |
| Streptococcal urinary tract infection | 3 | 0.01% |
| Pneumocystis test positive | 3 | 0.01% |
| Distributive shock | 3 | 0.01% |
| Tumour rupture | 3 | 0.01% |
| Faecal calprotectin increased | 3 | 0.01% |
| Peripheral artery thrombosis | 3 | 0.01% |
| Hepatocellular carcinoma | 3 | 0.01% |
| Pneumocystis jirovecii infection | 3 | 0.01% |
| Renal transplant failure | 3 | 0.01% |
| Vein collapse | 3 | 0.01% |
| Noninfective gingivitis | 3 | 0.01% |
| Peripheral venous disease | 3 | 0.01% |
| Bone marrow infiltration | 3 | 0.01% |
| Internal haemorrhage | 3 | 0.01% |
| Mouth swelling | 3 | 0.01% |
| BRAF gene mutation | 3 | 0.01% |
| Gastrointestinal wall thickening | 3 | 0.01% |
| Sinus node dysfunction | 3 | 0.01% |
| Acute motor-sensory axonal neuropathy | 3 | 0.01% |
| Acute motor axonal neuropathy | 3 | 0.01% |
| Complication associated with device | 3 | 0.01% |
| End stage renal disease | 3 | 0.01% |
| Product availability issue | 3 | 0.01% |
| Neuromyelitis optica spectrum disorder | 3 | 0.01% |
| Thyroid stimulating hormone deficiency | 3 | 0.01% |
| Intra-abdominal fluid collection | 3 | 0.01% |
| Cardiac dysfunction | 3 | 0.01% |
| Defaecation disorder | 3 | 0.01% |
| Primary biliary cholangitis | 3 | 0.01% |
| Incorrect route of product administration | 3 | 0.01% |
| Therapeutic product effect decreased | 3 | 0.01% |
| Hyperaesthesia teeth | 3 | 0.01% |
| Infusion related hypersensitivity reaction | 3 | 0.01% |
| Serous retinopathy | 3 | 0.01% |
| Immune-mediated renal disorder | 3 | 0.01% |
| Paranasal sinus inflammation | 3 | 0.01% |
| Suspected COVID-19 | 3 | 0.01% |
| Immune-mediated cholestasis | 3 | 0.01% |
| Immune-mediated neurological disorder | 3 | 0.01% |
| Colorectal adenoma | 3 | 0.01% |
| Acquired generalised lipodystrophy | 3 | 0.01% |
| Abnormal dreams | 2 | 0.01% |
| Abnormal sensation in eye | 2 | 0.01% |
| Abscess drainage | 2 | 0.01% |
| Acetabulum fracture | 2 | 0.01% |
| Adrenal haemorrhage | 2 | 0.01% |
| Adrenal suppression | 2 | 0.01% |
| Alanine aminotransferase | 2 | 0.01% |
| Alcoholism | 2 | 0.01% |
| Amenorrhoea | 2 | 0.01% |
| Amoebic dysentery | 2 | 0.01% |
| Anaphylactoid reaction | 2 | 0.01% |
| Ankylosing spondylitis | 2 | 0.01% |
| Aortic aneurysm | 2 | 0.01% |
| Aortic thrombosis | 2 | 0.01% |
| Aortic valve incompetence | 2 | 0.01% |
| Apnoea | 2 | 0.01% |
| Arachnoiditis | 2 | 0.01% |
| Areflexia | 2 | 0.01% |
| Arterial injury | 2 | 0.01% |
| Aspartate aminotransferase | 2 | 0.01% |
| Astigmatism | 2 | 0.01% |
| Atrial tachycardia | 2 | 0.01% |
| Azoospermia | 2 | 0.01% |
| B-cell lymphoma | 2 | 0.01% |
| Balanoposthitis | 2 | 0.01% |
| Biliary fistula | 2 | 0.01% |
| Bladder neoplasm | 2 | 0.01% |
| Blood blister | 2 | 0.01% |
| Blood calcium abnormal | 2 | 0.01% |
| Blood cholesterol decreased | 2 | 0.01% |
| Blood creatine phosphokinase MB abnormal | 2 | 0.01% |
| Blood creatinine decreased | 2 | 0.01% |
| Blood fibrinogen increased | 2 | 0.01% |
| Blood follicle stimulating hormone decreased | 2 | 0.01% |
| Blood follicle stimulating hormone increased | 2 | 0.01% |
| Blood osmolarity increased | 2 | 0.01% |
| Blood pH increased | 2 | 0.01% |
| Blood pressure diastolic increased | 2 | 0.01% |
| Blood pressure immeasurable | 2 | 0.01% |
| Blood pressure systolic decreased | 2 | 0.01% |
| Blood prolactin abnormal | 2 | 0.01% |
| Blood prolactin decreased | 2 | 0.01% |
| Blood prolactin increased | 2 | 0.01% |
| Blood thyroid stimulating hormone | 2 | 0.01% |
| Bone neoplasm | 2 | 0.01% |
| Bradypnoea | 2 | 0.01% |
| Brain compression | 2 | 0.01% |
| Breast enlargement | 2 | 0.01% |
| Budd-Chiari syndrome | 2 | 0.01% |
| Burkitt's lymphoma | 2 | 0.01% |
| Caesarean section | 2 | 0.01% |
| Cardiac sarcoidosis | 2 | 0.01% |
| Cellulitis staphylococcal | 2 | 0.01% |
| Cerebellar infarction | 2 | 0.01% |
| Cerebrospinal fluid leakage | 2 | 0.01% |
| Cervix carcinoma | 2 | 0.01% |
| Chronic hepatitis | 2 | 0.01% |
| Coccidioidomycosis | 2 | 0.01% |
| Coccydynia | 2 | 0.01% |
| Conduction disorder | 2 | 0.01% |
| Conjunctival haemorrhage | 2 | 0.01% |
| Corneal oedema | 2 | 0.01% |
| Creatinine renal clearance decreased | 2 | 0.01% |
| CSF protein increased | 2 | 0.01% |
| Cutaneous T-cell lymphoma | 2 | 0.01% |
| Cystitis haemorrhagic | 2 | 0.01% |
| Cytomegalovirus hepatitis | 2 | 0.01% |
| Dandruff | 2 | 0.01% |
| Decreased immune responsiveness | 2 | 0.01% |
| Dermal cyst | 2 | 0.01% |
| Dermatitis diaper | 2 | 0.01% |
| Dermatophytosis of nail | 2 | 0.01% |
| Diabetic hyperosmolar coma | 2 | 0.01% |
| Diabetic ketoacidotic hyperglycaemic coma | 2 | 0.01% |
| Diarrhoea infectious | 2 | 0.01% |
| Disability | 2 | 0.01% |
| Diverticulum | 2 | 0.01% |
| Dyspnoea at rest | 2 | 0.01% |
| Eczema herpeticum | 2 | 0.01% |
| Electrocardiogram ST segment depression | 2 | 0.01% |
| Electrocardiogram T wave inversion | 2 | 0.01% |
| Electrolyte depletion | 2 | 0.01% |
| Encephalitis toxic | 2 | 0.01% |
| Encephalitis viral | 2 | 0.01% |
| Endoscopic retrograde cholangiopancreatography | 2 | 0.01% |
| Ephelides | 2 | 0.01% |
| Epstein-Barr virus infection reactivation | 2 | 0.01% |
| Eructation | 2 | 0.01% |
| Erythema of eyelid | 2 | 0.01% |
| Euphoric mood | 2 | 0.01% |
| Faeces pale | 2 | 0.01% |
| Finger amputation | 2 | 0.01% |
| Food allergy | 2 | 0.01% |
| Fournier's gangrene | 2 | 0.01% |
| Gangrene | 2 | 0.01% |
| Gastric ulcer perforation | 2 | 0.01% |
| Gingival swelling | 2 | 0.01% |
| Glioblastoma | 2 | 0.01% |
| Glomerular filtration rate abnormal | 2 | 0.01% |
| Glomerulonephritis minimal lesion | 2 | 0.01% |
| Glossodynia | 2 | 0.01% |
| Gonadotrophin deficiency | 2 | 0.01% |
| Graft versus host disease | 2 | 0.01% |
| Granulocyte count increased | 2 | 0.01% |
| Haemochromatosis | 2 | 0.01% |
| Haemothorax | 2 | 0.01% |
| Hallucination, auditory | 2 | 0.01% |
| Hand fracture | 2 | 0.01% |
| Heart transplant rejection | 2 | 0.01% |
| Hepatic haemorrhage | 2 | 0.01% |
| Hepatic rupture | 2 | 0.01% |
| Hepatic vein thrombosis | 2 | 0.01% |
| Hepatitis A | 2 | 0.01% |
| Hepatitis B | 2 | 0.01% |
| Hepatitis C | 2 | 0.01% |
| Human herpesvirus 6 infection | 2 | 0.01% |
| Hydroureter | 2 | 0.01% |
| Hyperacusis | 2 | 0.01% |
| Hyperadrenocorticism | 2 | 0.01% |
| Hyperaesthesia | 2 | 0.01% |
| Hypermetabolism | 2 | 0.01% |
| Hypermetropia | 2 | 0.01% |
| Hyperproteinaemia | 2 | 0.01% |
| Hypertonia | 2 | 0.01% |
| Hyperventilation | 2 | 0.01% |
| Hypervolaemia | 2 | 0.01% |
| Hypogammaglobulinaemia | 2 | 0.01% |
| Hypohidrosis | 2 | 0.01% |
| Hyporeflexia | 2 | 0.01% |
| Ileostomy | 2 | 0.01% |
| Immunoglobulins decreased | 2 | 0.01% |
| Impetigo | 2 | 0.01% |
| Increased tendency to bruise | 2 | 0.01% |
| Infertility | 2 | 0.01% |
| Injection site haematoma | 2 | 0.01% |
| Injection site reaction | 2 | 0.01% |
| Intertrigo | 2 | 0.01% |
| Intestinal ulcer | 2 | 0.01% |
| Jugular vein thrombosis | 2 | 0.01% |
| Ketosis-prone diabetes mellitus | 2 | 0.01% |
| Labyrinthitis | 2 | 0.01% |
| Lagophthalmos | 2 | 0.01% |
| Left ventricular failure | 2 | 0.01% |
| Leg amputation | 2 | 0.01% |
| Lip oedema | 2 | 0.01% |
| Lip pain | 2 | 0.01% |
| Lip ulceration | 2 | 0.01% |
| Livedo reticularis | 2 | 0.01% |
| Lumbar spinal stenosis | 2 | 0.01% |
| Lung adenocarcinoma | 2 | 0.01% |
| Lupus nephritis | 2 | 0.01% |
| Lymphocytic leukaemia | 2 | 0.01% |
| Macular degeneration | 2 | 0.01% |
| Marrow hyperplasia | 2 | 0.01% |
| Mastectomy | 2 | 0.01% |
| Mean cell haemoglobin concentration decreased | 2 | 0.01% |
| Meniere's disease | 2 | 0.01% |
| Menopausal symptoms | 2 | 0.01% |
| Metastases to breast | 2 | 0.01% |
| Micturition urgency | 2 | 0.01% |
| Middle insomnia | 2 | 0.01% |
| Mole excision | 2 | 0.01% |
| Mononeuropathy multiplex | 2 | 0.01% |
| Mucosal ulceration | 2 | 0.01% |
| Multiple allergies | 2 | 0.01% |
| Multiple fractures | 2 | 0.01% |
| Muscle contractions involuntary | 2 | 0.01% |
| Muscle rigidity | 2 | 0.01% |
| Myopia | 2 | 0.01% |
| Nasal dryness | 2 | 0.01% |
| Neoplasm skin | 2 | 0.01% |
| Nephrectomy | 2 | 0.01% |
| Nephritis allergic | 2 | 0.01% |
| Neuralgic amyotrophy | 2 | 0.01% |
| Neuritis cranial | 2 | 0.01% |
| Nodular melanoma | 2 | 0.01% |
| Non-cardiogenic pulmonary oedema | 2 | 0.01% |
| Normal newborn | 2 | 0.01% |
| Oedema genital | 2 | 0.01% |
| Onychomycosis | 2 | 0.01% |
| Ophthalmic herpes zoster | 2 | 0.01% |
| Otitis externa | 2 | 0.01% |
| Overweight | 2 | 0.01% |
| Oxygen saturation abnormal | 2 | 0.01% |
| Pancreatic injury | 2 | 0.01% |
| Pancreatitis necrotising | 2 | 0.01% |
| Panic reaction | 2 | 0.01% |
| Panniculitis lobular | 2 | 0.01% |
| Paranoia | 2 | 0.01% |
| Parapsoriasis | 2 | 0.01% |
| Paresis | 2 | 0.01% |
| Penile ulceration | 2 | 0.01% |
| Periarthritis | 2 | 0.01% |
| Pharyngeal haemorrhage | 2 | 0.01% |
| Pharyngeal ulceration | 2 | 0.01% |
| Pharyngitis streptococcal | 2 | 0.01% |
| Pituitary infarction | 2 | 0.01% |
| Pneumonia legionella | 2 | 0.01% |
| Polyglandular autoimmune syndrome type II | 2 | 0.01% |
| Priapism | 2 | 0.01% |
| Protein total abnormal | 2 | 0.01% |
| Prothrombin time prolonged | 2 | 0.01% |
| Pterygium | 2 | 0.01% |
| Pulmonary infarction | 2 | 0.01% |
| Pulpitis dental | 2 | 0.01% |
| Pyelonephritis acute | 2 | 0.01% |
| Q fever | 2 | 0.01% |
| Radiation cataract | 2 | 0.01% |
| Rectal ulcer | 2 | 0.01% |
| Renal arteritis | 2 | 0.01% |
| Renal haemorrhage | 2 | 0.01% |
| Renal infarct | 2 | 0.01% |
| Renal vein thrombosis | 2 | 0.01% |
| Retinal depigmentation | 2 | 0.01% |
| Retinal melanoma | 2 | 0.01% |
| Retinal pigment epitheliopathy | 2 | 0.01% |
| Scleritis | 2 | 0.01% |
| Scrotal oedema | 2 | 0.01% |
| Seborrhoeic dermatitis | 2 | 0.01% |
| Second primary malignancy | 2 | 0.01% |
| Septic embolus | 2 | 0.01% |
| Serum ferritin increased | 2 | 0.01% |
| Serum sickness | 2 | 0.01% |
| Sinus congestion | 2 | 0.01% |
| Sinus pain | 2 | 0.01% |
| Skin odour abnormal | 2 | 0.01% |
| Small cell lung cancer | 2 | 0.01% |
| Sputum discoloured | 2 | 0.01% |
| Strabismus | 2 | 0.01% |
| Stress fracture | 2 | 0.01% |
| Superior vena cava syndrome | 2 | 0.01% |
| Swelling of eyelid | 2 | 0.01% |
| Synovial rupture | 2 | 0.01% |
| Tension headache | 2 | 0.01% |
| Testicular disorder | 2 | 0.01% |
| Testicular swelling | 2 | 0.01% |
| Thrombectomy | 2 | 0.01% |
| Thyroiditis chronic | 2 | 0.01% |
| Tinea cruris | 2 | 0.01% |
| Tongue disorder | 2 | 0.01% |
| Tonsillar hypertrophy | 2 | 0.01% |
| Torsade de pointes | 2 | 0.01% |
| Toxocariasis | 2 | 0.01% |
| Transferrin decreased | 2 | 0.01% |
| Tri-iodothyronine increased | 2 | 0.01% |
| Twin pregnancy | 2 | 0.01% |
| Typhoid fever | 2 | 0.01% |
| Unintended pregnancy | 2 | 0.01% |
| Urine abnormality | 2 | 0.01% |
| Urine flow decreased | 2 | 0.01% |
| Uterine disorder | 2 | 0.01% |
| Vaginal infection | 2 | 0.01% |
| Varicella | 2 | 0.01% |
| Vein disorder | 2 | 0.01% |
| Vena cava thrombosis | 2 | 0.01% |
| Ventricular fibrillation | 2 | 0.01% |
| Vertigo positional | 2 | 0.01% |
| Vitamin K deficiency | 2 | 0.01% |
| Vitreous haemorrhage | 2 | 0.01% |
| Vulvovaginal dryness | 2 | 0.01% |
| White blood cell count abnormal | 2 | 0.01% |
| White blood cells urine positive | 2 | 0.01% |
| Hydrothorax | 2 | 0.01% |
| Hypereosinophilic syndrome | 2 | 0.01% |
| Ischaemic cardiomyopathy | 2 | 0.01% |
| Vertebral lesion | 2 | 0.01% |
| Vena cava filter insertion | 2 | 0.01% |
| Encephalitis brain stem | 2 | 0.01% |
| Cytomegalovirus oesophagitis | 2 | 0.01% |
| Pancreatic mass | 2 | 0.01% |
| Osteopenia | 2 | 0.01% |
| Anaemia of malignant disease | 2 | 0.01% |
| Red blood cell sedimentation rate increased | 2 | 0.01% |
| Vocal cord paresis | 2 | 0.01% |
| Short-bowel syndrome | 2 | 0.01% |
| Precancerous skin lesion | 2 | 0.01% |
| Bone density decreased | 2 | 0.01% |
| Muscle fatigue | 2 | 0.01% |
| Upper respiratory tract inflammation | 2 | 0.01% |
| Spinal shock | 2 | 0.01% |
| Metastases to stomach | 2 | 0.01% |
| Metastases to pituitary gland | 2 | 0.01% |
| Cervical vertebral fracture | 2 | 0.01% |
| Thoracic vertebral fracture | 2 | 0.01% |
| Bradyphrenia | 2 | 0.01% |
| Mitochondrial cytopathy | 2 | 0.01% |
| Thirst decreased | 2 | 0.01% |
| Face injury | 2 | 0.01% |
| Anastomotic leak | 2 | 0.01% |
| Ventricular hypokinesia | 2 | 0.01% |
| Walking aid user | 2 | 0.01% |
| Leukocyturia | 2 | 0.01% |
| Liver carcinoma ruptured | 2 | 0.01% |
| Streptococcal bacteraemia | 2 | 0.01% |
| Nodular regenerative hyperplasia | 2 | 0.01% |
| Dyschezia | 2 | 0.01% |
| Hyperhomocysteinaemia | 2 | 0.01% |
| Central nervous system inflammation | 2 | 0.01% |
| Intestinal mucosal hypertrophy | 2 | 0.01% |
| Necrotising colitis | 2 | 0.01% |
| Bile duct stent insertion | 2 | 0.01% |
| Propionibacterium infection | 2 | 0.01% |
| Febrile infection | 2 | 0.01% |
| Haemodynamic instability | 2 | 0.01% |
| Ear discomfort | 2 | 0.01% |
| Eye oedema | 2 | 0.01% |
| Eosinophil percentage increased | 2 | 0.01% |
| Monocyte percentage increased | 2 | 0.01% |
| Catheter site erythema | 2 | 0.01% |
| Systemic mycosis | 2 | 0.01% |
| Biliary cyst | 2 | 0.01% |
| Neuroendocrine tumour | 2 | 0.01% |
| Paranasal sinus discomfort | 2 | 0.01% |
| Ocular neoplasm | 2 | 0.01% |
| Ocular vascular disorder | 2 | 0.01% |
| Catheter placement | 2 | 0.01% |
| Epigastric discomfort | 2 | 0.01% |
| Therapeutic response delayed | 2 | 0.01% |
| Splenic neoplasm malignancy unspecified | 2 | 0.01% |
| Fracture displacement | 2 | 0.01% |
| Secondary hyperthyroidism | 2 | 0.01% |
| Skin swelling | 2 | 0.01% |
| Brain natriuretic peptide increased | 2 | 0.01% |
| Secretion discharge | 2 | 0.01% |
| Infusion site swelling | 2 | 0.01% |
| Type III immune complex mediated reaction | 2 | 0.01% |
| Bone swelling | 2 | 0.01% |
| Vascular occlusion | 2 | 0.01% |
| Tri-iodothyronine free abnormal | 2 | 0.01% |
| Evans syndrome | 2 | 0.01% |
| Red cell distribution width increased | 2 | 0.01% |
| Cerebral haematoma | 2 | 0.01% |
| Cardiac ventricular thrombosis | 2 | 0.01% |
| Postoperative ileus | 2 | 0.01% |
| Enterococcal sepsis | 2 | 0.01% |
| Helicobacter gastritis | 2 | 0.01% |
| Haemorrhoidal haemorrhage | 2 | 0.01% |
| Vertebral column mass | 2 | 0.01% |
| Myoclonic epilepsy | 2 | 0.01% |
| Prostate examination abnormal | 2 | 0.01% |
| Fluid intake reduced | 2 | 0.01% |
| Faecaloma | 2 | 0.01% |
| Mediastinal haemorrhage | 2 | 0.01% |
| Food craving | 2 | 0.01% |
| Acquired epidermolysis bullosa | 2 | 0.01% |
| Vulvovaginal pruritus | 2 | 0.01% |
| Neurological examination abnormal | 2 | 0.01% |
| Subacute hepatic failure | 2 | 0.01% |
| Pharyngeal erythema | 2 | 0.01% |
| Periorbital cellulitis | 2 | 0.01% |
| Infected varicose vein | 2 | 0.01% |
| Daydreaming | 2 | 0.01% |
| Retinal injury | 2 | 0.01% |
| Lymph node palpable | 2 | 0.01% |
| Procedural complication | 2 | 0.01% |
| Computerised tomogram thorax abnormal | 2 | 0.01% |
| Chronic cutaneous lupus erythematosus | 2 | 0.01% |
| Wound sepsis | 2 | 0.01% |
| Wound abscess | 2 | 0.01% |
| Incision site pain | 2 | 0.01% |
| Lung neoplasm malignant | 2 | 0.01% |
| Cytomegalovirus infection reactivation | 2 | 0.01% |
| Skin oedema | 2 | 0.01% |
| Sopor | 2 | 0.01% |
| Meningitis noninfective | 2 | 0.01% |
| Eyelash discolouration | 2 | 0.01% |
| Fungal sepsis | 2 | 0.01% |
| Inferior vena caval occlusion | 2 | 0.01% |
| Intestinal mass | 2 | 0.01% |
| Gastrointestinal ischaemia | 2 | 0.01% |
| Juvenile idiopathic arthritis | 2 | 0.01% |
| Early satiety | 2 | 0.01% |
| Acute hepatitis B | 2 | 0.01% |
| Epidermal necrosis | 2 | 0.01% |
| Protein-losing gastroenteropathy | 2 | 0.01% |
| Postrenal failure | 2 | 0.01% |
| Bacterial test positive | 2 | 0.01% |
| Wound infection pseudomonas | 2 | 0.01% |
| Apoptosis | 2 | 0.01% |
| Blood alkaline phosphatase decreased | 2 | 0.01% |
| CSF test abnormal | 2 | 0.01% |
| Pharyngeal hypoaesthesia | 2 | 0.01% |
| Antinuclear antibody positive | 2 | 0.01% |
| Liver function test | 2 | 0.01% |
| Antineutrophil cytoplasmic antibody positive | 2 | 0.01% |
| Myxoedema coma | 2 | 0.01% |
| Choroidal neovascularisation | 2 | 0.01% |
| Hyperphosphatasaemia | 2 | 0.01% |
| Recall phenomenon | 2 | 0.01% |
| Tinea infection | 2 | 0.01% |
| Calcium ionised increased | 2 | 0.01% |
| Abdominal symptom | 2 | 0.01% |
| Adenovirus infection | 2 | 0.01% |
| Abdominal hernia | 2 | 0.01% |
| Benign neoplasm | 2 | 0.01% |
| Cardiac operation | 2 | 0.01% |
| Central nervous system infection | 2 | 0.01% |
| Coombs test positive | 2 | 0.01% |
| Enterococcal infection | 2 | 0.01% |
| Haematotoxicity | 2 | 0.01% |
| Helminthic infection | 2 | 0.01% |
| Iris disorder | 2 | 0.01% |
| Klebsiella infection | 2 | 0.01% |
| Mantle cell lymphoma | 2 | 0.01% |
| Mental disorder due to a general medical condition | 2 | 0.01% |
| Streptococcal infection | 2 | 0.01% |
| Facial nerve disorder | 2 | 0.01% |
| Feeling of body temperature change | 2 | 0.01% |
| Postoperative wound complication | 2 | 0.01% |
| Proteus infection | 2 | 0.01% |
| Neurofibrosarcoma | 2 | 0.01% |
| Pituitary tumour benign | 2 | 0.01% |
| Ulcer haemorrhage | 2 | 0.01% |
| Lower limb fracture | 2 | 0.01% |
| Amputation | 2 | 0.01% |
| Colon operation | 2 | 0.01% |
| Obstruction | 2 | 0.01% |
| Occult blood positive | 2 | 0.01% |
| Cranial nerve paralysis | 2 | 0.01% |
| Pulmonary function test decreased | 2 | 0.01% |
| Salivary gland disorder | 2 | 0.01% |
| Gastrointestinal obstruction | 2 | 0.01% |
| Leukoplakia | 2 | 0.01% |
| Liver operation | 2 | 0.01% |
| Respiratory tract infection viral | 2 | 0.01% |
| Metabolic encephalopathy | 2 | 0.01% |
| Metastases to soft tissue | 2 | 0.01% |
| Neisseria infection | 2 | 0.01% |
| Urine analysis abnormal | 2 | 0.01% |
| Unevaluable event | 2 | 0.01% |
| Erythema migrans | 2 | 0.01% |
| Erosive duodenitis | 2 | 0.01% |
| Tooth fracture | 2 | 0.01% |
| Peripheral arterial occlusive disease | 2 | 0.01% |
| Gallbladder enlargement | 2 | 0.01% |
| Haemorrhagic diathesis | 2 | 0.01% |
| Increased upper airway secretion | 2 | 0.01% |
| Hyperamylasaemia | 2 | 0.01% |
| Dysplastic naevus | 2 | 0.01% |
| Pruritus allergic | 2 | 0.01% |
| Coagulation test abnormal | 2 | 0.01% |
| Cell marker increased | 2 | 0.01% |
| Incision site cellulitis | 2 | 0.01% |
| Tumour invasion | 2 | 0.01% |
| KL-6 increased | 2 | 0.01% |
| Exfoliative rash | 2 | 0.01% |
| Graft versus host disease in skin | 2 | 0.01% |
| Nail growth abnormal | 2 | 0.01% |
| Waist circumference increased | 2 | 0.01% |
| Tumour perforation | 2 | 0.01% |
| Skin abrasion | 2 | 0.01% |
| Nutritional condition abnormal | 2 | 0.01% |
| Latent tuberculosis | 2 | 0.01% |
| Enterocolitis bacterial | 2 | 0.01% |
| Thymus enlargement | 2 | 0.01% |
| Percutaneous coronary intervention | 2 | 0.01% |
| Bronchial hyperreactivity | 2 | 0.01% |
| Interleukin-2 receptor increased | 2 | 0.01% |
| Orbital apex syndrome | 2 | 0.01% |
| Prostatic calcification | 2 | 0.01% |
| Neutrophilic panniculitis | 2 | 0.01% |
| Surfactant protein increased | 2 | 0.01% |
| Endotracheal intubation | 2 | 0.01% |
| Neurosensory hypoacusis | 2 | 0.01% |
| Disease complication | 2 | 0.01% |
| Toxic neuropathy | 2 | 0.01% |
| Graft versus host disease in lung | 2 | 0.01% |
| Dementia with Lewy bodies | 2 | 0.01% |
| Probiotic therapy | 2 | 0.01% |
| Gastrointestinal surgery | 2 | 0.01% |
| Foreign body reaction | 2 | 0.01% |
| Gastroenteritis norovirus | 2 | 0.01% |
| Tumour compression | 2 | 0.01% |
| Neurological decompensation | 2 | 0.01% |
| Stomatococcal infection | 2 | 0.01% |
| Drain removal | 2 | 0.01% |
| Pineal gland cyst | 2 | 0.01% |
| Vulvovaginal pain | 2 | 0.01% |
| Liquid product physical issue | 2 | 0.01% |
| Osmotic demyelination syndrome | 2 | 0.01% |
| Traumatic lung injury | 2 | 0.01% |
| Bladder dysfunction | 2 | 0.01% |
| Coma scale abnormal | 2 | 0.01% |
| Device issue | 2 | 0.01% |
| Product complaint | 2 | 0.01% |
| Citrobacter test positive | 2 | 0.01% |
| Escherichia test positive | 2 | 0.01% |
| Influenza A virus test positive | 2 | 0.01% |
| Urobilinogen urine increased | 2 | 0.01% |
| Cerebral small vessel ischaemic disease | 2 | 0.01% |
| Craniocerebral injury | 2 | 0.01% |
| Pancreatic cyst rupture | 2 | 0.01% |
| Metastatic lymphoma | 2 | 0.01% |
| Infectious pleural effusion | 2 | 0.01% |
| Stiff person syndrome | 2 | 0.01% |
| Enzyme level increased | 2 | 0.01% |
| Eye colour change | 2 | 0.01% |
| Peritumoural oedema | 2 | 0.01% |
| White matter lesion | 2 | 0.01% |
| Protein deficiency | 2 | 0.01% |
| Meniscus injury | 2 | 0.01% |
| Malignant neoplasm of unknown primary site | 2 | 0.01% |
| Adrenal gland cancer | 2 | 0.01% |
| Abdominal lymphadenopathy | 2 | 0.01% |
| Exposure during pregnancy | 2 | 0.01% |
| Colonic abscess | 2 | 0.01% |
| Escherichia pyelonephritis | 2 | 0.01% |
| Splenic artery thrombosis | 2 | 0.01% |
| Granulomatous dermatitis | 2 | 0.01% |
| Hyperferritinaemia | 2 | 0.01% |
| Chorioretinal folds | 2 | 0.01% |
| Varicella zoster virus infection | 2 | 0.01% |
| Primary hypoparathyroidism | 2 | 0.01% |
| Administration site extravasation | 2 | 0.01% |
| Feeding intolerance | 2 | 0.01% |
| Inability to afford medication | 2 | 0.01% |
| Stenosis | 2 | 0.01% |
| Lacunar stroke | 2 | 0.01% |
| Vascular stent stenosis | 2 | 0.01% |
| Proctectomy | 2 | 0.01% |
| Bilirubin urine present | 2 | 0.01% |
| Neurosarcoidosis | 2 | 0.01% |
| Contraindicated product administered | 2 | 0.01% |
| Nasal injury | 2 | 0.01% |
| Obstructive pancreatitis | 2 | 0.01% |
| Hyposplenism | 2 | 0.01% |
| Autoimmune anaemia | 2 | 0.01% |
| Food refusal | 2 | 0.01% |
| Irregular sleep wake rhythm disorder | 2 | 0.01% |
| Product prescribing issue | 2 | 0.01% |
| Tissue infiltration | 2 | 0.01% |
| Heliotrope rash | 2 | 0.01% |
| Malignant gastrointestinal obstruction | 2 | 0.01% |
| Sensitive skin | 2 | 0.01% |
| Pernio-like erythema | 2 | 0.01% |
| Therapeutic product effect delayed | 2 | 0.01% |
| Therapeutic product effect variable | 2 | 0.01% |
| Mucosal disorder | 2 | 0.01% |
| AST/ALT ratio abnormal | 2 | 0.01% |
| Eosinophilic gastritis | 2 | 0.01% |
| Autoimmune cholangitis | 2 | 0.01% |
| Autoimmune blistering disease | 2 | 0.01% |
| Axonal and demyelinating polyneuropathy | 2 | 0.01% |
| Discouragement | 2 | 0.01% |
| Asymptomatic COVID-19 | 2 | 0.01% |
| Immune-mediated cytopenia | 2 | 0.01% |
| Sarcoid-like reaction | 2 | 0.01% |
| Immune-mediated oesophagitis | 2 | 0.01% |
| Heavy menstrual bleeding | 2 | 0.01% |
| Myocardial injury | 2 | 0.01% |
| Superficial vein thrombosis | 2 | 0.01% |
| Abdominal adhesions | 1 | 0.004% |
| Acalculia | 1 | 0.004% |
| Acanthosis | 1 | 0.004% |
| Acidosis hyperchloraemic | 1 | 0.004% |
| Activated partial thromboplastin time shortened | 1 | 0.004% |
| Acute disseminated encephalomyelitis | 1 | 0.004% |
| Acute leukaemia | 1 | 0.004% |
| Acute promyelocytic leukaemia | 1 | 0.004% |
| Adenocarcinoma of colon | 1 | 0.004% |
| Adenoma benign | 1 | 0.004% |
| Adrenal adenoma | 1 | 0.004% |
| Adrenal atrophy | 1 | 0.004% |
| Adrenal cortex necrosis | 1 | 0.004% |
| Adrenal gland injury | 1 | 0.004% |
| Aesthesioneuroblastoma | 1 | 0.004% |
| Air embolism | 1 | 0.004% |
| Akathisia | 1 | 0.004% |
| Albinism | 1 | 0.004% |
| Albumin globulin ratio abnormal | 1 | 0.004% |
| Albumin globulin ratio decreased | 1 | 0.004% |
| Alcohol abuse | 1 | 0.004% |
| Alcohol interaction | 1 | 0.004% |
| Alexia | 1 | 0.004% |
| Alpha 1 foetoprotein increased | 1 | 0.004% |
| Amyotrophic lateral sclerosis | 1 | 0.004% |
| Anaemia of chronic disease | 1 | 0.004% |
| Anal ulcer | 1 | 0.004% |
| Angiocentric lymphoma | 1 | 0.004% |
| Angioimmunoblastic T-cell lymphoma | 1 | 0.004% |
| Animal bite | 1 | 0.004% |
| Anion gap increased | 1 | 0.004% |
| Anoxia | 1 | 0.004% |
| Anterograde amnesia | 1 | 0.004% |
| Aortic valve sclerosis | 1 | 0.004% |
| Aortic valve stenosis | 1 | 0.004% |
| Apallic syndrome | 1 | 0.004% |
| Appendicectomy | 1 | 0.004% |
| Appendicitis perforated | 1 | 0.004% |
| Apraxia | 1 | 0.004% |
| Arteriovenous malformation | 1 | 0.004% |
| Arteriospasm coronary | 1 | 0.004% |
| Arteritis | 1 | 0.004% |
| Arthropod sting | 1 | 0.004% |
| Aspartate aminotransferase decreased | 1 | 0.004% |
| Aspiration joint | 1 | 0.004% |
| Auditory disorder | 1 | 0.004% |
| Auricular swelling | 1 | 0.004% |
| Axillary vein thrombosis | 1 | 0.004% |
| B-cell lymphoma stage IV | 1 | 0.004% |
| Bacterial diarrhoea | 1 | 0.004% |
| Barrett's oesophagus | 1 | 0.004% |
| Basophil count decreased | 1 | 0.004% |
| Basophil count increased | 1 | 0.004% |
| Basosquamous carcinoma | 1 | 0.004% |
| Behcet's syndrome | 1 | 0.004% |
| Bile duct cancer | 1 | 0.004% |
| Biopsy kidney abnormal | 1 | 0.004% |
| Biopsy liver | 1 | 0.004% |
| Biopsy muscle | 1 | 0.004% |
| Biopsy skin | 1 | 0.004% |
| Bipolar I disorder | 1 | 0.004% |
| Bladder catheterisation | 1 | 0.004% |
| Bladder dilatation | 1 | 0.004% |
| Bladder discomfort | 1 | 0.004% |
| Bladder irritation | 1 | 0.004% |
| Bladder pain | 1 | 0.004% |
| Bladder sphincter atony | 1 | 0.004% |
| Blastomycosis | 1 | 0.004% |
| Blepharochalasis | 1 | 0.004% |
| Blindness cortical | 1 | 0.004% |
| Blood albumin abnormal | 1 | 0.004% |
| Blood aldosterone abnormal | 1 | 0.004% |
| Blood aluminium increased | 1 | 0.004% |
| Blood antidiuretic hormone abnormal | 1 | 0.004% |
| Blood bicarbonate decreased | 1 | 0.004% |
| Blood bicarbonate increased | 1 | 0.004% |
| Blood calcitonin increased | 1 | 0.004% |
| Blood chloride increased | 1 | 0.004% |
| Blood cholinesterase decreased | 1 | 0.004% |
| Blood culture positive | 1 | 0.004% |
| Blood folate decreased | 1 | 0.004% |
| Blood follicle stimulating hormone abnormal | 1 | 0.004% |
| Blood immunoglobulin G decreased | 1 | 0.004% |
| Blood immunoglobulin G increased | 1 | 0.004% |
| Blood immunoglobulin M abnormal | 1 | 0.004% |
| Blood immunoglobulin M decreased | 1 | 0.004% |
| Blood insulin abnormal | 1 | 0.004% |
| Blood insulin increased | 1 | 0.004% |
| Blood luteinising hormone abnormal | 1 | 0.004% |
| Blood luteinising hormone decreased | 1 | 0.004% |
| Blood magnesium increased | 1 | 0.004% |
| Blood oestrogen abnormal | 1 | 0.004% |
| Blood osmolarity decreased | 1 | 0.004% |
| Blood parathyroid hormone decreased | 1 | 0.004% |
| Blood pressure fluctuation | 1 | 0.004% |
| Blood pressure systolic abnormal | 1 | 0.004% |
| Blood triglycerides abnormal | 1 | 0.004% |
| Blood urea abnormal | 1 | 0.004% |
| Blood uric acid decreased | 1 | 0.004% |
| Body temperature | 1 | 0.004% |
| Body temperature decreased | 1 | 0.004% |
| Body tinea | 1 | 0.004% |
| Bone sarcoma | 1 | 0.004% |
| Borderline ovarian tumour | 1 | 0.004% |
| Bowen's disease | 1 | 0.004% |
| Brachial plexus injury | 1 | 0.004% |
| Bradykinesia | 1 | 0.004% |
| Brain hypoxia | 1 | 0.004% |
| Brain neoplasm benign | 1 | 0.004% |
| Brain stem haemorrhage | 1 | 0.004% |
| Brain stem infarction | 1 | 0.004% |
| Breast cellulitis | 1 | 0.004% |
| Breast disorder male | 1 | 0.004% |
| Breath odour | 1 | 0.004% |
| Bronchial obstruction | 1 | 0.004% |
| Bruxism | 1 | 0.004% |
| Bundle branch block | 1 | 0.004% |
| Bundle branch block left | 1 | 0.004% |
| Burn oesophageal | 1 | 0.004% |
| Burns third degree | 1 | 0.004% |
| Calculus bladder | 1 | 0.004% |
| Calculus urinary | 1 | 0.004% |
| Capillary disorder | 1 | 0.004% |
| Cardiac hypertrophy | 1 | 0.004% |
| Cardiac murmur | 1 | 0.004% |
| Cardiac output increased | 1 | 0.004% |
| Cardiolipin antibody positive | 1 | 0.004% |
| Carpal tunnel decompression | 1 | 0.004% |
| Cartilage injury | 1 | 0.004% |
| Cataract cortical | 1 | 0.004% |
| Cataract nuclear | 1 | 0.004% |
| Cataract subcapsular | 1 | 0.004% |
| Central nervous system lymphoma | 1 | 0.004% |
| Central nervous system neoplasm | 1 | 0.004% |
| Cerebellar ataxia | 1 | 0.004% |
| Cerebellar haemorrhage | 1 | 0.004% |
| Cerebrovascular disorder | 1 | 0.004% |
| Cervicobrachial syndrome | 1 | 0.004% |
| Change of bowel habit | 1 | 0.004% |
| Chemical poisoning | 1 | 0.004% |
| Cholangiolitis | 1 | 0.004% |
| Chondritis | 1 | 0.004% |
| Chordoma | 1 | 0.004% |
| Chorea | 1 | 0.004% |
| Chromatopsia | 1 | 0.004% |
| Chronic fatigue syndrome | 1 | 0.004% |
| Clumsiness | 1 | 0.004% |
| CNS ventriculitis | 1 | 0.004% |
| Coagulation time prolonged | 1 | 0.004% |
| Colonoscopy | 1 | 0.004% |
| Colonoscopy abnormal | 1 | 0.004% |
| Colony stimulating factor therapy | 1 | 0.004% |
| Colour blindness | 1 | 0.004% |
| Colour blindness acquired | 1 | 0.004% |
| Compression fracture | 1 | 0.004% |
| Concussion | 1 | 0.004% |
| Convulsions local | 1 | 0.004% |
| Coronary artery bypass | 1 | 0.004% |
| Costochondritis | 1 | 0.004% |
| Crepitations | 1 | 0.004% |
| CREST syndrome | 1 | 0.004% |
| Cryptococcosis | 1 | 0.004% |
| CSF granulocyte count abnormal | 1 | 0.004% |
| Culture urine positive | 1 | 0.004% |
| Cystitis interstitial | 1 | 0.004% |
| Decreased interest | 1 | 0.004% |
| Dengue fever | 1 | 0.004% |
| Derailment | 1 | 0.004% |
| Derealisation | 1 | 0.004% |
| Dermatitis atopic | 1 | 0.004% |
| Dermatitis herpetiformis | 1 | 0.004% |
| Diffuse large B-cell lymphoma | 1 | 0.004% |
| Dislocation of vertebra | 1 | 0.004% |
| Dizziness exertional | 1 | 0.004% |
| Dressler's syndrome | 1 | 0.004% |
| Drop attacks | 1 | 0.004% |
| Drug dependence | 1 | 0.004% |
| Drug level decreased | 1 | 0.004% |
| Drug level increased | 1 | 0.004% |
| Duodenal obstruction | 1 | 0.004% |
| Duodenal perforation | 1 | 0.004% |
| Duodenal ulcer haemorrhage | 1 | 0.004% |
| Dupuytren's contracture | 1 | 0.004% |
| Dyschromatopsia | 1 | 0.004% |
| Dysdiadochokinesis | 1 | 0.004% |
| Dyshidrotic eczema | 1 | 0.004% |
| Dysmenorrhoea | 1 | 0.004% |
| Dysphoria | 1 | 0.004% |
| Dyspraxia | 1 | 0.004% |
| Dystonia | 1 | 0.004% |
| Eczema asteatotic | 1 | 0.004% |
| Eczema nummular | 1 | 0.004% |
| Ejaculation failure | 1 | 0.004% |
| Electrocardiogram QRS complex prolonged | 1 | 0.004% |
| Electrocardiogram ST segment abnormal | 1 | 0.004% |
| Electrocardiogram ST segment elevation | 1 | 0.004% |
| Embolism arterial | 1 | 0.004% |
| Empyema | 1 | 0.004% |
| Endotoxic shock | 1 | 0.004% |
| Enophthalmos | 1 | 0.004% |
| Enterococcal bacteraemia | 1 | 0.004% |
| Enterovirus infection | 1 | 0.004% |
| Enthesopathy | 1 | 0.004% |
| Enuresis | 1 | 0.004% |
| Eosinophilic myocarditis | 1 | 0.004% |
| Erythema annulare | 1 | 0.004% |
| Exostosis | 1 | 0.004% |
| Extradural haematoma | 1 | 0.004% |
| Extrapyramidal disorder | 1 | 0.004% |
| Eye allergy | 1 | 0.004% |
| Eye haemorrhage | 1 | 0.004% |
| Factor VII deficiency | 1 | 0.004% |
| Failure to anastomose | 1 | 0.004% |
| Fat tissue increased | 1 | 0.004% |
| Fibrin degradation products | 1 | 0.004% |
| Fibrinolysis | 1 | 0.004% |
| Fibroadenoma of breast | 1 | 0.004% |
| Fixed eruption | 1 | 0.004% |
| Flat affect | 1 | 0.004% |
| Folate deficiency | 1 | 0.004% |
| Forced expiratory volume decreased | 1 | 0.004% |
| Forearm fracture | 1 | 0.004% |
| Foreign body in eye | 1 | 0.004% |
| Fractured sacrum | 1 | 0.004% |
| Frostbite | 1 | 0.004% |
| Gallbladder injury | 1 | 0.004% |
| Gamma-glutamyltransferase | 1 | 0.004% |
| Gastric dilatation | 1 | 0.004% |
| Gastric perforation | 1 | 0.004% |
| Gastroduodenal ulcer | 1 | 0.004% |
| Gastroenteritis clostridial | 1 | 0.004% |
| Gastroenteritis staphylococcal | 1 | 0.004% |
| Gastrointestinal angiodysplasia | 1 | 0.004% |
| Generalised anxiety disorder | 1 | 0.004% |
| Generalised resistance to thyroid hormone | 1 | 0.004% |
| Genital herpes | 1 | 0.004% |
| Gestational diabetes | 1 | 0.004% |
| Gingival erosion | 1 | 0.004% |
| Gliosis | 1 | 0.004% |
| Globulins increased | 1 | 0.004% |
| Glomerular filtration rate | 1 | 0.004% |
| Glomerulonephritis chronic | 1 | 0.004% |
| Glomerulonephritis membranous | 1 | 0.004% |
| Gram stain positive | 1 | 0.004% |
| Granuloma annulare | 1 | 0.004% |
| Gynaecomastia | 1 | 0.004% |
| Haemangioma of liver | 1 | 0.004% |
| Haemoglobinuria | 1 | 0.004% |
| Haemorrhage subcutaneous | 1 | 0.004% |
| Haemorrhagic disorder | 1 | 0.004% |
| Hairy cell leukaemia | 1 | 0.004% |
| Hallucination, tactile | 1 | 0.004% |
| Hallucinations, mixed | 1 | 0.004% |
| Hangover | 1 | 0.004% |
| Hemianopia | 1 | 0.004% |
| Hemianopia homonymous | 1 | 0.004% |
| Henoch-Schonlein purpura | 1 | 0.004% |
| Hepatic fibrosis | 1 | 0.004% |
| Hepatitis G | 1 | 0.004% |
| Hepatitis viral | 1 | 0.004% |
| Herpes simplex encephalitis | 1 | 0.004% |
| Hidradenitis | 1 | 0.004% |
| Hip arthroplasty | 1 | 0.004% |
| Hodgkin's disease | 1 | 0.004% |
| Hordeolum | 1 | 0.004% |
| Hormone replacement therapy | 1 | 0.004% |
| Horner's syndrome | 1 | 0.004% |
| Hyperadrenalism | 1 | 0.004% |
| Hyperaemia | 1 | 0.004% |
| Hyperaldosteronism | 1 | 0.004% |
| Hyperammonaemia | 1 | 0.004% |
| Hypercalcaemia of malignancy | 1 | 0.004% |
| Hyperchlorhydria | 1 | 0.004% |
| Hypercholesterolaemia | 1 | 0.004% |
| Hypergammaglobulinaemia benign monoclonal | 1 | 0.004% |
| Hyperlactacidaemia | 1 | 0.004% |
| Hypertensive encephalopathy | 1 | 0.004% |
| Hyperthermia malignant | 1 | 0.004% |
| Hypertonic bladder | 1 | 0.004% |
| Hypertrichosis | 1 | 0.004% |
| Hypertrophic cardiomyopathy | 1 | 0.004% |
| Hypochloraemia | 1 | 0.004% |
| Hypocoagulable state | 1 | 0.004% |
| Hypoglycaemic coma | 1 | 0.004% |
| Hypogonadism male | 1 | 0.004% |
| Hypouricaemia | 1 | 0.004% |
| Hypovitaminosis | 1 | 0.004% |
| Ileal perforation | 1 | 0.004% |
| Ileal ulcer | 1 | 0.004% |
| Iliac artery embolism | 1 | 0.004% |
| Illusion | 1 | 0.004% |
| Inadequate diet | 1 | 0.004% |
| Incisional hernia | 1 | 0.004% |
| Indifference | 1 | 0.004% |
| Infected skin ulcer | 1 | 0.004% |
| Inguinal hernia repair | 1 | 0.004% |
| Inguinal hernia, obstructive | 1 | 0.004% |
| Injection site extravasation | 1 | 0.004% |
| Injection site infection | 1 | 0.004% |
| Injection site mass | 1 | 0.004% |
| Injection site pain | 1 | 0.004% |
| Injection site rash | 1 | 0.004% |
| Insulin resistance | 1 | 0.004% |
| Insulin resistant diabetes | 1 | 0.004% |
| Intentional overdose | 1 | 0.004% |
| Internal fixation of fracture | 1 | 0.004% |
| Intestinal infarction | 1 | 0.004% |
| Intracranial aneurysm | 1 | 0.004% |
| Intraventricular haemorrhage | 1 | 0.004% |
| Iron deficiency | 1 | 0.004% |
| Jejunal ulcer | 1 | 0.004% |
| Jejunostomy | 1 | 0.004% |
| Joint contracture | 1 | 0.004% |
| Joint injection | 1 | 0.004% |
| Keloid scar | 1 | 0.004% |
| Ketonuria | 1 | 0.004% |
| Large granular lymphocytosis | 1 | 0.004% |
| Laryngeal ulceration | 1 | 0.004% |
| Leptospirosis | 1 | 0.004% |
| Leukaemoid reaction | 1 | 0.004% |
| Ligament sprain | 1 | 0.004% |
| Limb reduction defect | 1 | 0.004% |
| Linear IgA disease | 1 | 0.004% |
| Lip discolouration | 1 | 0.004% |
| Lip dry | 1 | 0.004% |
| Lipoatrophy | 1 | 0.004% |
| Liposarcoma | 1 | 0.004% |
| Listeriosis | 1 | 0.004% |
| Liver tenderness | 1 | 0.004% |
| Lividity | 1 | 0.004% |
| Lung lobectomy | 1 | 0.004% |
| Local reaction | 1 | 0.004% |
| Long QT syndrome | 1 | 0.004% |
| Loss of control of legs | 1 | 0.004% |
| Low density lipoprotein increased | 1 | 0.004% |
| Lumbar puncture abnormal | 1 | 0.004% |
| Lymph node tuberculosis | 1 | 0.004% |
| Lymphangiectasia | 1 | 0.004% |
| Lymphoplasmacytoid lymphoma/immunocytoma | 1 | 0.004% |
| Malabsorption | 1 | 0.004% |
| Malignant melanoma stage IV | 1 | 0.004% |
| Malignant neoplasm of conjunctiva | 1 | 0.004% |
| Malignant neoplasm of eye | 1 | 0.004% |
| Marasmus | 1 | 0.004% |
| Mastocytosis | 1 | 0.004% |
| Mastoiditis | 1 | 0.004% |
| Mean cell haemoglobin concentration increased | 1 | 0.004% |
| Mean cell haemoglobin increased | 1 | 0.004% |
| Mean cell volume decreased | 1 | 0.004% |
| Measles | 1 | 0.004% |
| Mediastinal fibrosis | 1 | 0.004% |
| Mediastinal mass | 1 | 0.004% |
| Meningitis herpes | 1 | 0.004% |
| Meningitis staphylococcal | 1 | 0.004% |
| Meningoencephalitis herpetic | 1 | 0.004% |
| Menstruation irregular | 1 | 0.004% |
| Mesenteric artery embolism | 1 | 0.004% |
| Mesenteric artery stenosis | 1 | 0.004% |
| Metabolic abnormality management | 1 | 0.004% |
| Metastases to pleura | 1 | 0.004% |
| Migraine with aura | 1 | 0.004% |
| Mixed connective tissue disease | 1 | 0.004% |
| Monocyte count decreased | 1 | 0.004% |
| Monocytosis | 1 | 0.004% |
| Mononucleosis syndrome | 1 | 0.004% |
| Motion sickness | 1 | 0.004% |
| Moyamoya disease | 1 | 0.004% |
| Multiple injuries | 1 | 0.004% |
| Mumps | 1 | 0.004% |
| Muscle discomfort | 1 | 0.004% |
| Muscular sarcoidosis | 1 | 0.004% |
| Myoglobin blood increased | 1 | 0.004% |
| Myositis-like syndrome | 1 | 0.004% |
| Nail avulsion | 1 | 0.004% |
| Nail discolouration | 1 | 0.004% |
| Nail dystrophy | 1 | 0.004% |
| Nail pitting | 1 | 0.004% |
| Narcolepsy | 1 | 0.004% |
| Nasal polyps | 1 | 0.004% |
| Nasal septum deviation | 1 | 0.004% |
| Nasal septum perforation | 1 | 0.004% |
| Nasal sinus cancer | 1 | 0.004% |
| Nerve root injury lumbar | 1 | 0.004% |
| Neuroblastoma | 1 | 0.004% |
| Neutrophil count | 1 | 0.004% |
| Night blindness | 1 | 0.004% |
| Nocardiosis | 1 | 0.004% |
| Nodular vasculitis | 1 | 0.004% |
| Non-small cell lung cancer stage III | 1 | 0.004% |
| Non-Hodgkin's lymphoma | 1 | 0.004% |
| Nonspecific reaction | 1 | 0.004% |
| Normal pressure hydrocephalus | 1 | 0.004% |
| Normochromic anaemia | 1 | 0.004% |
| Nosocomial infection | 1 | 0.004% |
| Obstructive sleep apnoea syndrome | 1 | 0.004% |
| Oesophageal haemorrhage | 1 | 0.004% |
| Oesophageal perforation | 1 | 0.004% |
| Oesophageal spasm | 1 | 0.004% |
| Oesophagectomy | 1 | 0.004% |
| Oestradiol decreased | 1 | 0.004% |
| Onycholysis | 1 | 0.004% |
| Opportunistic infection | 1 | 0.004% |
| Optic nerve cupping | 1 | 0.004% |
| Oral hairy leukoplakia | 1 | 0.004% |
| Orbital oedema | 1 | 0.004% |
| Organic brain syndrome | 1 | 0.004% |
| Oropharyngeal squamous cell carcinoma | 1 | 0.004% |
| Orthopnoea | 1 | 0.004% |
| Osteomalacia | 1 | 0.004% |
| Osteosarcoma | 1 | 0.004% |
| Osteosclerosis | 1 | 0.004% |
| Otitis media acute | 1 | 0.004% |
| Otitis media chronic | 1 | 0.004% |
| Otosclerosis | 1 | 0.004% |
| Ovarian cyst ruptured | 1 | 0.004% |
| Ovarian enlargement | 1 | 0.004% |
| Ovarian failure | 1 | 0.004% |
| Palmoplantar keratoderma | 1 | 0.004% |
| Pancreatic pseudocyst | 1 | 0.004% |
| Pancreatitis chronic | 1 | 0.004% |
| Pancreatitis relapsing | 1 | 0.004% |
| Pancreatogenous diabetes | 1 | 0.004% |
| Parasite stool test positive | 1 | 0.004% |
| Parathyroid tumour benign | 1 | 0.004% |
| Parotidectomy | 1 | 0.004% |
| Peliosis hepatis | 1 | 0.004% |
| Pelvic abscess | 1 | 0.004% |
| Pelvic venous thrombosis | 1 | 0.004% |
| Perforation bile duct | 1 | 0.004% |
| Pericardial drainage | 1 | 0.004% |
| Pericardial haemorrhage | 1 | 0.004% |
| Perinephric abscess | 1 | 0.004% |
| Periorbital pain | 1 | 0.004% |
| Peripheral circulatory failure | 1 | 0.004% |
| Peripheral T-cell lymphoma unspecified | 1 | 0.004% |
| Peripheral vascular disorder | 1 | 0.004% |
| Peritoneal abscess | 1 | 0.004% |
| pH urine abnormal | 1 | 0.004% |
| Pharyngeal cancer | 1 | 0.004% |
| Phimosis | 1 | 0.004% |
| Pickwickian syndrome | 1 | 0.004% |
| Piloerection | 1 | 0.004% |
| Pituitary tumour | 1 | 0.004% |
| Pituitary-dependent Cushing's syndrome | 1 | 0.004% |
| Pityriasis rubra pilaris | 1 | 0.004% |
| Placental disorder | 1 | 0.004% |
| Placental insufficiency | 1 | 0.004% |
| Plantar fasciitis | 1 | 0.004% |
| Plasmacytoma | 1 | 0.004% |
| Platelet morphology abnormal | 1 | 0.004% |
| Pleocytosis | 1 | 0.004% |
| Pleural thickening | 1 | 0.004% |
| Pneumonia escherichia | 1 | 0.004% |
| Pneumonia influenzal | 1 | 0.004% |
| Pneumonia mycoplasmal | 1 | 0.004% |
| Pneumonia pneumococcal | 1 | 0.004% |
| Pneumonia pseudomonal | 1 | 0.004% |
| Pneumonia respiratory syncytial viral | 1 | 0.004% |
| Pneumonia staphylococcal | 1 | 0.004% |
| Pneumonia streptococcal | 1 | 0.004% |
| Pneumonia viral | 1 | 0.004% |
| Pneumonitis chemical | 1 | 0.004% |
| Pneumothorax spontaneous | 1 | 0.004% |
| PO2 decreased | 1 | 0.004% |
| Polyarteritis nodosa | 1 | 0.004% |
| Polycythaemia | 1 | 0.004% |
| Polycythaemia vera | 1 | 0.004% |
| Polyglandular autoimmune syndrome type I | 1 | 0.004% |
| Poor peripheral circulation | 1 | 0.004% |
| Portal hypertension | 1 | 0.004% |
| Post herpetic neuralgia | 1 | 0.004% |
| Posture abnormal | 1 | 0.004% |
| Pouchitis | 1 | 0.004% |
| Pre-existing condition improved | 1 | 0.004% |
| Pregnancy | 1 | 0.004% |
| Premature labour | 1 | 0.004% |
| Premature menopause | 1 | 0.004% |
| Presbyopia | 1 | 0.004% |
| Prinzmetal angina | 1 | 0.004% |
| Prostate cancer metastatic | 1 | 0.004% |
| Prostate cancer recurrent | 1 | 0.004% |
| Prostatic disorder | 1 | 0.004% |
| Prothrombin time shortened | 1 | 0.004% |
| Pruritus genital | 1 | 0.004% |
| Pulmonary vasculitis | 1 | 0.004% |
| Pulse absent | 1 | 0.004% |
| Pupil fixed | 1 | 0.004% |
| Pustular psoriasis | 1 | 0.004% |
| Pylorospasm | 1 | 0.004% |
| Radiation mucositis | 1 | 0.004% |
| Radiation myelopathy | 1 | 0.004% |
| Radiation pneumonitis | 1 | 0.004% |
| Rebound effect | 1 | 0.004% |
| Rectal polyp | 1 | 0.004% |
| Rectal ulcer haemorrhage | 1 | 0.004% |
| Rectosigmoid cancer | 1 | 0.004% |
| Red blood cell count abnormal | 1 | 0.004% |
| Red blood cell schistocytes present | 1 | 0.004% |
| Refraction disorder | 1 | 0.004% |
| Renal amyloidosis | 1 | 0.004% |
| Renal aneurysm | 1 | 0.004% |
| Renal atrophy | 1 | 0.004% |
| Renal cancer | 1 | 0.004% |
| Renal colic | 1 | 0.004% |
| Renal necrosis | 1 | 0.004% |
| Renal transplant | 1 | 0.004% |
| Renal tubular disorder | 1 | 0.004% |
| Resorption bone increased | 1 | 0.004% |
| Respiratory rate increased | 1 | 0.004% |
| Restrictive cardiomyopathy | 1 | 0.004% |
| Resuscitation | 1 | 0.004% |
| Reticulocytopenia | 1 | 0.004% |
| Reticulocytosis | 1 | 0.004% |
| Reticuloendothelial system stimulated | 1 | 0.004% |
| Retinal degeneration | 1 | 0.004% |
| Retinal tear | 1 | 0.004% |
| Rhinitis allergic | 1 | 0.004% |
| Right ventricular failure | 1 | 0.004% |
| Sacroiliitis | 1 | 0.004% |
| Sarcomatosis | 1 | 0.004% |
| Schwannoma | 1 | 0.004% |
| Scoliosis | 1 | 0.004% |
| Screaming | 1 | 0.004% |
| Scrotal swelling | 1 | 0.004% |
| Serotonin syndrome | 1 | 0.004% |
| Sexual dysfunction | 1 | 0.004% |
| Shock symptom | 1 | 0.004% |
| Sideroblastic anaemia | 1 | 0.004% |
| Simple partial seizures | 1 | 0.004% |
| Sinus headache | 1 | 0.004% |
| Sinus polyp | 1 | 0.004% |
| Skin discomfort | 1 | 0.004% |
| Skin hypertrophy | 1 | 0.004% |
| Skin warm | 1 | 0.004% |
| Skin wrinkling | 1 | 0.004% |
| SLE arthritis | 1 | 0.004% |
| Sleep disorder due to general medical condition, insomnia type | 1 | 0.004% |
| Small intestinal resection | 1 | 0.004% |
| Speech disorder developmental | 1 | 0.004% |
| Spinal cord injury | 1 | 0.004% |
| Splenic abscess | 1 | 0.004% |
| Splenic rupture | 1 | 0.004% |
| Squamous cell carcinoma of the oral cavity | 1 | 0.004% |
| Squamous cell carcinoma of the vulva | 1 | 0.004% |
| Staphylococcal abscess | 1 | 0.004% |
| Starvation | 1 | 0.004% |
| Stasis dermatitis | 1 | 0.004% |
| Steatorrhoea | 1 | 0.004% |
| Sterile pyuria | 1 | 0.004% |
| Sternal fracture | 1 | 0.004% |
| Still's disease | 1 | 0.004% |
| Strangulated hernia | 1 | 0.004% |
| Strongyloidiasis | 1 | 0.004% |
| Stupor | 1 | 0.004% |
| Subclavian artery thrombosis | 1 | 0.004% |
| Subcutaneous emphysema | 1 | 0.004% |
| Subdural haemorrhage | 1 | 0.004% |
| Superficial injury of eye | 1 | 0.004% |
| Superior sagittal sinus thrombosis | 1 | 0.004% |
| Supraventricular extrasystoles | 1 | 0.004% |
| Sweat gland disorder | 1 | 0.004% |
| Systemic lupus erythematosus rash | 1 | 0.004% |
| Systemic mastocytosis | 1 | 0.004% |
| Teeth brittle | 1 | 0.004% |
| Telangiectasia | 1 | 0.004% |
| Tension | 1 | 0.004% |
| Thymoma | 1 | 0.004% |
| Thyroid atrophy | 1 | 0.004% |
| Thyroid neoplasm | 1 | 0.004% |
| Thyroiditis subacute | 1 | 0.004% |
| Tibia fracture | 1 | 0.004% |
| Tic | 1 | 0.004% |
| Tongue blistering | 1 | 0.004% |
| Tongue paralysis | 1 | 0.004% |
| Tonic convulsion | 1 | 0.004% |
| Toxic nodular goitre | 1 | 0.004% |
| Tracheitis | 1 | 0.004% |
| Tracheostomy | 1 | 0.004% |
| Transfusion reaction | 1 | 0.004% |
| Transitional cell carcinoma | 1 | 0.004% |
| Traumatic haematoma | 1 | 0.004% |
| Tricuspid valve incompetence | 1 | 0.004% |
| Trifascicular block | 1 | 0.004% |
| Trigeminal neuralgia | 1 | 0.004% |
| Trigger finger | 1 | 0.004% |
| Ulna fracture | 1 | 0.004% |
| Urethral disorder | 1 | 0.004% |
| Urethritis | 1 | 0.004% |
| Urge incontinence | 1 | 0.004% |
| Urinary casts | 1 | 0.004% |
| Urinary tract infection enterococcal | 1 | 0.004% |
| Urogenital disorder | 1 | 0.004% |
| Urticaria vesiculosa | 1 | 0.004% |
| Uterine haemorrhage | 1 | 0.004% |
| Uterine inflammation | 1 | 0.004% |
| Varicocele | 1 | 0.004% |
| Varicose vein | 1 | 0.004% |
| Vascular fragility | 1 | 0.004% |
| Vasculitic rash | 1 | 0.004% |
| Vasoconstriction | 1 | 0.004% |
| Vasospasm | 1 | 0.004% |
| VIIIth nerve injury | 1 | 0.004% |
| Vitamin B12 decreased | 1 | 0.004% |
| VIth nerve injury | 1 | 0.004% |
| Vitrectomy | 1 | 0.004% |
| Vitreous opacities | 1 | 0.004% |
| Volume blood increased | 1 | 0.004% |
| White blood cell count | 1 | 0.004% |
| Xeroderma | 1 | 0.004% |
| Yawning | 1 | 0.004% |
| Brief psychotic disorder with marked stressors | 1 | 0.004% |
| Pseudarthrosis | 1 | 0.004% |
| Subdural hygroma | 1 | 0.004% |
| Agnosia | 1 | 0.004% |
| Oral infection | 1 | 0.004% |
| Multiple-drug resistance | 1 | 0.004% |
| Pneumocephalus | 1 | 0.004% |
| Poor venous access | 1 | 0.004% |
| Axillary pain | 1 | 0.004% |
| Dermatosis | 1 | 0.004% |
| Crystal deposit intestine | 1 | 0.004% |
| Urticarial vasculitis | 1 | 0.004% |
| Cerebral ventricle dilatation | 1 | 0.004% |
| Onychoclasis | 1 | 0.004% |
| Retinal fovea disorder | 1 | 0.004% |
| Angiolipoma | 1 | 0.004% |
| Rectal abscess | 1 | 0.004% |
| Paradoxical drug reaction | 1 | 0.004% |
| Vascular pseudoaneurysm | 1 | 0.004% |
| Gastrostomy | 1 | 0.004% |
| Sinobronchitis | 1 | 0.004% |
| Bladder spasm | 1 | 0.004% |
| Scar pain | 1 | 0.004% |
| HELLP syndrome | 1 | 0.004% |
| Weight loss poor | 1 | 0.004% |
| Pharyngotonsillitis | 1 | 0.004% |
| Antiacetylcholine receptor antibody positive | 1 | 0.004% |
| Necrotising gastritis | 1 | 0.004% |
| Allergic sinusitis | 1 | 0.004% |
| Fractured coccyx | 1 | 0.004% |
| Red blood cell agglutination | 1 | 0.004% |
| Haematocrit abnormal | 1 | 0.004% |
| Food aversion | 1 | 0.004% |
| Thermal burns of eye | 1 | 0.004% |
| Onychomadesis | 1 | 0.004% |
| Bronchoalveolar lavage | 1 | 0.004% |
| Skin turgor decreased | 1 | 0.004% |
| Fuchs' syndrome | 1 | 0.004% |
| Traumatic fracture | 1 | 0.004% |
| Knee operation | 1 | 0.004% |
| Shoulder arthroplasty | 1 | 0.004% |
| Cholecystocholangitis | 1 | 0.004% |
| Fungal oesophagitis | 1 | 0.004% |
| Gallbladder polyp | 1 | 0.004% |
| Metastases to bladder | 1 | 0.004% |
| Metastases to eye | 1 | 0.004% |
| Pubic pain | 1 | 0.004% |
| Bradyarrhythmia | 1 | 0.004% |
| Left ventricular hypertrophy | 1 | 0.004% |
| Extraskeletal ossification | 1 | 0.004% |
| Urinary sediment present | 1 | 0.004% |
| Blood HIV RNA increased | 1 | 0.004% |
| Dry gangrene | 1 | 0.004% |
| Lipogranuloma | 1 | 0.004% |
| Mental status changes postoperative | 1 | 0.004% |
| Nephrostomy | 1 | 0.004% |
| Abulia | 1 | 0.004% |
| Sudden onset of sleep | 1 | 0.004% |
| Duodenal stenosis | 1 | 0.004% |
| Application site bruise | 1 | 0.004% |
| Cervical radiculopathy | 1 | 0.004% |
| Anal inflammation | 1 | 0.004% |
| Faecal volume decreased | 1 | 0.004% |
| Cardiovascular deconditioning | 1 | 0.004% |
| Self esteem decreased | 1 | 0.004% |
| Ammonia | 1 | 0.004% |
| Enema administration | 1 | 0.004% |
| Renal tubular dysfunction | 1 | 0.004% |
| Oropharyngeal candidiasis | 1 | 0.004% |
| Skin ulcer haemorrhage | 1 | 0.004% |
| Electrocardiogram T wave abnormal | 1 | 0.004% |
| Eyelid tumour | 1 | 0.004% |
| Chest tube insertion | 1 | 0.004% |
| Mean platelet volume | 1 | 0.004% |
| Left ventricular enlargement | 1 | 0.004% |
| Postoperative hypertension | 1 | 0.004% |
| Prostate infection | 1 | 0.004% |
| Epstein-Barr virus test | 1 | 0.004% |
| Self-medication | 1 | 0.004% |
| Band neutrophil count increased | 1 | 0.004% |
| Urine bilirubin increased | 1 | 0.004% |
| Tracheal stenosis | 1 | 0.004% |
| Lymph gland infection | 1 | 0.004% |
| Anti-neutrophil cytoplasmic antibody positive vasculitis | 1 | 0.004% |
| Lymphorrhoea | 1 | 0.004% |
| Macular hole | 1 | 0.004% |
| Hip surgery | 1 | 0.004% |
| Wrist surgery | 1 | 0.004% |
| Endodontic procedure | 1 | 0.004% |
| Catheter site haemorrhage | 1 | 0.004% |
| Drug ineffective for unapproved indication | 1 | 0.004% |
| Madarosis | 1 | 0.004% |
| Photodermatosis | 1 | 0.004% |
| Soft tissue haemorrhage | 1 | 0.004% |
| Exercise tolerance decreased | 1 | 0.004% |
| Ischaemic neuropathy | 1 | 0.004% |
| Mechanical ileus | 1 | 0.004% |
| Dysentery | 1 | 0.004% |
| Viral diarrhoea | 1 | 0.004% |
| Tolosa-Hunt syndrome | 1 | 0.004% |
| Cardiac neoplasm unspecified | 1 | 0.004% |
| Corneal defect | 1 | 0.004% |
| Chest wall abscess | 1 | 0.004% |
| Laziness | 1 | 0.004% |
| Scrotal angiokeratoma | 1 | 0.004% |
| Metastases to bone marrow | 1 | 0.004% |
| Metastases to oesophagus | 1 | 0.004% |
| Metastases to retroperitoneum | 1 | 0.004% |
| Gastritis viral | 1 | 0.004% |
| Eschar | 1 | 0.004% |
| Encephalomalacia | 1 | 0.004% |
| Skin induration | 1 | 0.004% |
| Tongue cyst | 1 | 0.004% |
| Enterobacter infection | 1 | 0.004% |
| Oesophageal polyp | 1 | 0.004% |
| Hepatic artery occlusion | 1 | 0.004% |
| Lip erosion | 1 | 0.004% |
| Pancreatic islets hyperplasia | 1 | 0.004% |
| Neuroborreliosis | 1 | 0.004% |
| Oscillopsia | 1 | 0.004% |
| Iodine allergy | 1 | 0.004% |
| Eyelid margin crusting | 1 | 0.004% |
| Ear congestion | 1 | 0.004% |
| Lymphocytic lymphoma | 1 | 0.004% |
| Basophil percentage increased | 1 | 0.004% |
| Neutrophil percentage decreased | 1 | 0.004% |
| Catheter site pain | 1 | 0.004% |
| Epstein-Barr virus antibody positive | 1 | 0.004% |
| Herpes oesophagitis | 1 | 0.004% |
| Brain contusion | 1 | 0.004% |
| Oedematous pancreatitis | 1 | 0.004% |
| Gastrointestinal hypermotility | 1 | 0.004% |
| Impaired self-care | 1 | 0.004% |
| Nasal discomfort | 1 | 0.004% |
| Myofascitis | 1 | 0.004% |
| Palatal disorder | 1 | 0.004% |
| Electrocardiogram repolarisation abnormality | 1 | 0.004% |
| Fanconi syndrome acquired | 1 | 0.004% |
| Primary hypogonadism | 1 | 0.004% |
| Thoracic operation | 1 | 0.004% |
| Diaphragmalgia | 1 | 0.004% |
| Pancreatic duct obstruction | 1 | 0.004% |
| Drug tolerance | 1 | 0.004% |
| Perirectal abscess | 1 | 0.004% |
| Acquired oesophageal web | 1 | 0.004% |
| Eosinophilic pneumonia acute | 1 | 0.004% |
| Cardiac flutter | 1 | 0.004% |
| Skin bacterial infection | 1 | 0.004% |
| Oral pruritus | 1 | 0.004% |
| Lesion excision | 1 | 0.004% |
| Ileocolostomy | 1 | 0.004% |
| Emergency care examination | 1 | 0.004% |
| Gastrointestinal disorder therapy | 1 | 0.004% |
| Protein urine present | 1 | 0.004% |
| Bronchitis viral | 1 | 0.004% |
| Alcohol withdrawal syndrome | 1 | 0.004% |
| Candida sepsis | 1 | 0.004% |
| Epidermolysis | 1 | 0.004% |
| Walking disability | 1 | 0.004% |
| Insulin-requiring type 2 diabetes mellitus | 1 | 0.004% |
| Neurolysis | 1 | 0.004% |
| Bronchopleural fistula | 1 | 0.004% |
| Infusion site pain | 1 | 0.004% |
| Tracheal disorder | 1 | 0.004% |
| Scan bone marrow abnormal | 1 | 0.004% |
| Abscess neck | 1 | 0.004% |
| Neurodegenerative disorder | 1 | 0.004% |
| Pachymeningitis | 1 | 0.004% |
| Medical device discomfort | 1 | 0.004% |
| Saccadic eye movement | 1 | 0.004% |
| Fistula repair | 1 | 0.004% |
| Fibrous histiocytoma | 1 | 0.004% |
| Therapeutic procedure | 1 | 0.004% |
| B-lymphocyte abnormalities | 1 | 0.004% |
| Tri-iodothyronine free increased | 1 | 0.004% |
| Anti-insulin antibody positive | 1 | 0.004% |
| Partial lipodystrophy | 1 | 0.004% |
| Red cell distribution width decreased | 1 | 0.004% |
| Thyroglobulin increased | 1 | 0.004% |
| Thyroglobulin decreased | 1 | 0.004% |
| Klebsiella sepsis | 1 | 0.004% |
| Small intestine carcinoma | 1 | 0.004% |
| Intestinal resection | 1 | 0.004% |
| Affect lability | 1 | 0.004% |
| IVth nerve paresis | 1 | 0.004% |
| IIIrd nerve paresis | 1 | 0.004% |
| Citrobacter sepsis | 1 | 0.004% |
| Enterobacter pneumonia | 1 | 0.004% |
| Enterobacter sepsis | 1 | 0.004% |
| Helicobacter infection | 1 | 0.004% |
| Facet joint syndrome | 1 | 0.004% |
| Lipase abnormal | 1 | 0.004% |
| Prosthesis implantation | 1 | 0.004% |
| Splenic lesion | 1 | 0.004% |
| Mucosal discolouration | 1 | 0.004% |
| Inflammation of wound | 1 | 0.004% |
| Allergy to plants | 1 | 0.004% |
| Antidepressant therapy | 1 | 0.004% |
| Infusion site reaction | 1 | 0.004% |
| Infusion site infection | 1 | 0.004% |
| Pancreatic enlargement | 1 | 0.004% |
| Mean platelet volume decreased | 1 | 0.004% |
| Bone cancer metastatic | 1 | 0.004% |
| Hepatic cancer metastatic | 1 | 0.004% |
| Breast cancer metastatic | 1 | 0.004% |
| Necrotising oesophagitis | 1 | 0.004% |
| Haemorrhage urinary tract | 1 | 0.004% |
| Haematoma muscle | 1 | 0.004% |
| Scleroedema | 1 | 0.004% |
| Tinea versicolour | 1 | 0.004% |
| Pain management | 1 | 0.004% |
| Shift to the left | 1 | 0.004% |
| Growth hormone deficiency | 1 | 0.004% |
| Erythrosis | 1 | 0.004% |
| Corneal irritation | 1 | 0.004% |
| Psoas abscess | 1 | 0.004% |
| Therapeutic aspiration | 1 | 0.004% |
| Dehydroepiandrosterone decreased | 1 | 0.004% |
| Mucocutaneous rash | 1 | 0.004% |
| Gaze palsy | 1 | 0.004% |
| Tenosynovitis stenosans | 1 | 0.004% |
| Varicophlebitis | 1 | 0.004% |
| Dental care | 1 | 0.004% |
| Device failure | 1 | 0.004% |
| Near drowning | 1 | 0.004% |
| Necrobiosis lipoidica diabeticorum | 1 | 0.004% |
| Hydrocholecystis | 1 | 0.004% |
| Palatal oedema | 1 | 0.004% |
| Salivary gland mass | 1 | 0.004% |
| Eye laser surgery | 1 | 0.004% |
| Urine odour abnormal | 1 | 0.004% |
| Blast cells absent | 1 | 0.004% |
| Connective tissue inflammation | 1 | 0.004% |
| Parvovirus infection | 1 | 0.004% |
| Scleral oedema | 1 | 0.004% |
| Cardiac ventricular disorder | 1 | 0.004% |
| Peripheral artery occlusion | 1 | 0.004% |
| Tobacco user | 1 | 0.004% |
| Post procedural diarrhoea | 1 | 0.004% |
| Post procedural urine leak | 1 | 0.004% |
| Blood ketone body increased | 1 | 0.004% |
| Cardiac valve vegetation | 1 | 0.004% |
| Colon injury | 1 | 0.004% |
| Haematology test abnormal | 1 | 0.004% |
| Muscle enzyme increased | 1 | 0.004% |
| Globulins decreased | 1 | 0.004% |
| Multi-organ disorder | 1 | 0.004% |
| Neutrophilic dermatosis | 1 | 0.004% |
| Asteatosis | 1 | 0.004% |
| Heart alternation | 1 | 0.004% |
| Hypertensive emergency | 1 | 0.004% |
| Allergy to arthropod sting | 1 | 0.004% |
| Clostridium colitis | 1 | 0.004% |
| Dysplasia | 1 | 0.004% |
| Bladder mass | 1 | 0.004% |
| Hypothrombinaemia | 1 | 0.004% |
| Hypoperfusion | 1 | 0.004% |
| Right ventricular dysfunction | 1 | 0.004% |
| Spinal ligament ossification | 1 | 0.004% |
| Negative thoughts | 1 | 0.004% |
| Pelvic infection | 1 | 0.004% |
| Sinusitis fungal | 1 | 0.004% |
| Richter's syndrome | 1 | 0.004% |
| Mycobacterium avium complex infection | 1 | 0.004% |
| Genital tract inflammation | 1 | 0.004% |
| Bone marrow necrosis | 1 | 0.004% |
| Enteritis infectious | 1 | 0.004% |
| Clostridium bacteraemia | 1 | 0.004% |
| Pseudomonal sepsis | 1 | 0.004% |
| Hand dermatitis | 1 | 0.004% |
| Spinal deformity | 1 | 0.004% |
| General physical condition | 1 | 0.004% |
| Pseudomonal bacteraemia | 1 | 0.004% |
| Disturbance in sexual arousal | 1 | 0.004% |
| Thalamus haemorrhage | 1 | 0.004% |
| Putamen haemorrhage | 1 | 0.004% |
| Bile duct necrosis | 1 | 0.004% |
| Haemobilia | 1 | 0.004% |
| Rathke's cleft cyst | 1 | 0.004% |
| Superior vena cava occlusion | 1 | 0.004% |
| Venous occlusion | 1 | 0.004% |
| Iliac vein occlusion | 1 | 0.004% |
| Ventricular dysfunction | 1 | 0.004% |
| Biloma | 1 | 0.004% |
| Band neutrophil percentage increased | 1 | 0.004% |
| Oxygen consumption decreased | 1 | 0.004% |
| Adrenal haematoma | 1 | 0.004% |
| Eyelid pain | 1 | 0.004% |
| Lymphostasis | 1 | 0.004% |
| Infected lymphocele | 1 | 0.004% |
| Fungal test positive | 1 | 0.004% |
| Viral test positive | 1 | 0.004% |
| Allergic colitis | 1 | 0.004% |
| Neutropenic infection | 1 | 0.004% |
| Histone antibody | 1 | 0.004% |
| Endocrine test abnormal | 1 | 0.004% |
| Haemorrhagic ascites | 1 | 0.004% |
| Infusion site rash | 1 | 0.004% |
| Intelligence test abnormal | 1 | 0.004% |
| Blood mercury abnormal | 1 | 0.004% |
| Presbyoesophagus | 1 | 0.004% |
| Induration | 1 | 0.004% |
| Gastrointestinal erosion | 1 | 0.004% |
| Skin neoplasm bleeding | 1 | 0.004% |
| Ischaemic cerebral infarction | 1 | 0.004% |
| Post lumbar puncture syndrome | 1 | 0.004% |
| Freezing phenomenon | 1 | 0.004% |
| Postoperative adhesion | 1 | 0.004% |
| Anaesthetic complication | 1 | 0.004% |
| Arterial disorder | 1 | 0.004% |
| Arterial stenosis | 1 | 0.004% |
| Autoantibody test | 1 | 0.004% |
| Cardiac myxoma | 1 | 0.004% |
| Bursitis infective | 1 | 0.004% |
| Cerebellar haematoma | 1 | 0.004% |
| Colon neoplasm | 1 | 0.004% |
| Connective tissue neoplasm | 1 | 0.004% |
| Corynebacterium infection | 1 | 0.004% |
| Diabetic complication | 1 | 0.004% |
| Diaphragmatic disorder | 1 | 0.004% |
| DNA antibody positive | 1 | 0.004% |
| Endocrine pancreatic disorder | 1 | 0.004% |
| Eosinophil count abnormal | 1 | 0.004% |
| Ocular toxicity | 1 | 0.004% |
| Eyelid function disorder | 1 | 0.004% |
| Foot deformity | 1 | 0.004% |
| Foot operation | 1 | 0.004% |
| Gastrointestinal injury | 1 | 0.004% |
| Gastrointestinal neoplasm | 1 | 0.004% |
| Genitourinary tract infection | 1 | 0.004% |
| Lens disorder | 1 | 0.004% |
| Ligament disorder | 1 | 0.004% |
| Lipid metabolism disorder | 1 | 0.004% |
| Internal injury | 1 | 0.004% |
| Investigation abnormal | 1 | 0.004% |
| Jaw disorder | 1 | 0.004% |
| Joint lock | 1 | 0.004% |
| Malignant peritoneal neoplasm | 1 | 0.004% |
| Metastases to nervous system | 1 | 0.004% |
| Mineral deficiency | 1 | 0.004% |
| Mucosal haemorrhage | 1 | 0.004% |
| Nail bed infection | 1 | 0.004% |
| Pelvic neoplasm | 1 | 0.004% |
| Pericardial disease | 1 | 0.004% |
| Peritoneal disorder | 1 | 0.004% |
| Pleural neoplasm | 1 | 0.004% |
| Skeletal injury | 1 | 0.004% |
| Skull fracture | 1 | 0.004% |
| Spinal cord disorder | 1 | 0.004% |
| Synovial disorder | 1 | 0.004% |
| Temperature regulation disorder | 1 | 0.004% |
| Traumatic intracranial haemorrhage | 1 | 0.004% |
| Tricuspid valve disease | 1 | 0.004% |
| Tumour excision | 1 | 0.004% |
| Vaginal disorder | 1 | 0.004% |
| Cardiac valve disease | 1 | 0.004% |
| Visual pathway disorder | 1 | 0.004% |
| Colorectal cancer | 1 | 0.004% |
| Retroviral infection | 1 | 0.004% |
| Salivary gland neoplasm | 1 | 0.004% |
| Scleral disorder | 1 | 0.004% |
| Serratia infection | 1 | 0.004% |
| Inner ear disorder | 1 | 0.004% |
| Mass excision | 1 | 0.004% |
| Sensory level abnormal | 1 | 0.004% |
| Vitamin B complex deficiency | 1 | 0.004% |
| Vocal cord disorder | 1 | 0.004% |
| Adrenal neoplasm | 1 | 0.004% |
| Aortic valve disease | 1 | 0.004% |
| Cardiac fibrillation | 1 | 0.004% |
| Deformity | 1 | 0.004% |
| Adjustment disorder | 1 | 0.004% |
| Adrenalectomy | 1 | 0.004% |
| Anorectal infection | 1 | 0.004% |
| Atypical mycobacterial infection | 1 | 0.004% |
| Biliary tract infection | 1 | 0.004% |
| Blood electrolytes decreased | 1 | 0.004% |
| Blood test | 1 | 0.004% |
| Bone operation | 1 | 0.004% |
| Chondrocalcinosis | 1 | 0.004% |
| Chondropathy | 1 | 0.004% |
| CSF pressure abnormal | 1 | 0.004% |
| Enterocolitis viral | 1 | 0.004% |
| Extradural abscess | 1 | 0.004% |
| Extranodal marginal zone B-cell lymphoma (MALT type) | 1 | 0.004% |
| Occult blood negative | 1 | 0.004% |
| Oesophageal neoplasm | 1 | 0.004% |
| Pancreatic neoplasm | 1 | 0.004% |
| Bone scan abnormal | 1 | 0.004% |
| Brain scan abnormal | 1 | 0.004% |
| Lymph nodes scan abnormal | 1 | 0.004% |
| Gastric operation | 1 | 0.004% |
| Severe acute respiratory syndrome | 1 | 0.004% |
| Glomerulosclerosis | 1 | 0.004% |
| Heart valve replacement | 1 | 0.004% |
| Hepatectomy | 1 | 0.004% |
| Hernia repair | 1 | 0.004% |
| Herpes ophthalmic | 1 | 0.004% |
| Large intestine operation | 1 | 0.004% |
| Lip neoplasm | 1 | 0.004% |
| Lithotripsy | 1 | 0.004% |
| Lymphocyte morphology abnormal | 1 | 0.004% |
| Histology abnormal | 1 | 0.004% |
| Hyperlipidaemia | 1 | 0.004% |
| Renal mass | 1 | 0.004% |
| Splenic marginal zone lymphoma | 1 | 0.004% |
| Steroid therapy | 1 | 0.004% |
| Thyroidectomy | 1 | 0.004% |
| Spleen scan abnormal | 1 | 0.004% |
| Sensorimotor disorder | 1 | 0.004% |
| Viral load | 1 | 0.004% |
| Nasal disorder | 1 | 0.004% |
| Spinal cord neoplasm | 1 | 0.004% |
| Spinal operation | 1 | 0.004% |
| Urinary tract infection pseudomonal | 1 | 0.004% |
| Neuromuscular toxicity | 1 | 0.004% |
| Procedural hypotension | 1 | 0.004% |
| Joint hyperextension | 1 | 0.004% |
| Genital abscess | 1 | 0.004% |
| Middle ear effusion | 1 | 0.004% |
| Thrombosis in device | 1 | 0.004% |
| Vulvar erosion | 1 | 0.004% |
| Enterovesical fistula | 1 | 0.004% |
| Muscle contracture | 1 | 0.004% |
| Metamyelocyte percentage increased | 1 | 0.004% |
| Carditis | 1 | 0.004% |
| Hypercreatininaemia | 1 | 0.004% |
| Infective spondylitis | 1 | 0.004% |
| Chest wall mass | 1 | 0.004% |
| Facial spasm | 1 | 0.004% |
| Sarcopenia | 1 | 0.004% |
| Bronchoalveolar lavage abnormal | 1 | 0.004% |
| Acute left ventricular failure | 1 | 0.004% |
| Basilar artery thrombosis | 1 | 0.004% |
| Proctitis fungal | 1 | 0.004% |
| Progesterone decreased | 1 | 0.004% |
| Brain stem syndrome | 1 | 0.004% |
| CD4 lymphocytes increased | 1 | 0.004% |
| Interleukin level increased | 1 | 0.004% |
| Tryptase increased | 1 | 0.004% |
| International normalised ratio fluctuation | 1 | 0.004% |
| Bladder hypertrophy | 1 | 0.004% |
| Acarodermatitis | 1 | 0.004% |
| Body temperature fluctuation | 1 | 0.004% |
| Renal embolism | 1 | 0.004% |
| Spondylolisthesis | 1 | 0.004% |
| Excessive granulation tissue | 1 | 0.004% |
| Exposure to chemical pollution | 1 | 0.004% |
| Pyramidal tract syndrome | 1 | 0.004% |
| Vital functions abnormal | 1 | 0.004% |
| Post procedural oedema | 1 | 0.004% |
| Erosive oesophagitis | 1 | 0.004% |
| Vascular encephalopathy | 1 | 0.004% |
| Extramedullary haemopoiesis | 1 | 0.004% |
| Device malfunction | 1 | 0.004% |
| Undersensing | 1 | 0.004% |
| Lower respiratory tract infection bacterial | 1 | 0.004% |
| Sleep disorder due to a general medical condition | 1 | 0.004% |
| Orthostatic intolerance | 1 | 0.004% |
| Ileocolectomy | 1 | 0.004% |
| Tonsillolith | 1 | 0.004% |
| Polyglandular autoimmune syndrome type III | 1 | 0.004% |
| Splenic granuloma | 1 | 0.004% |
| Epstein-Barr virus test positive | 1 | 0.004% |
| Breakthrough pain | 1 | 0.004% |
| Gene mutation | 1 | 0.004% |
| Serum amyloid A protein increased | 1 | 0.004% |
| Faecal vomiting | 1 | 0.004% |
| Device dislocation | 1 | 0.004% |
| Device occlusion | 1 | 0.004% |
| Antinuclear antibody increased | 1 | 0.004% |
| Tachyphrenia | 1 | 0.004% |
| Micrographic skin surgery | 1 | 0.004% |
| Pancreatic duct dilatation | 1 | 0.004% |
| Bronchial disorder | 1 | 0.004% |
| Cellulitis of male external genital organ | 1 | 0.004% |
| Age-related macular degeneration | 1 | 0.004% |
| Head titubation | 1 | 0.004% |
| Thalamic infarction | 1 | 0.004% |
| Myocardial oedema | 1 | 0.004% |
| Rectal fissure | 1 | 0.004% |
| Epstein-Barr viraemia | 1 | 0.004% |
| Bacterial rhinitis | 1 | 0.004% |
| Cystitis bacterial | 1 | 0.004% |
| Tonsillitis bacterial | 1 | 0.004% |
| Dermo-hypodermitis | 1 | 0.004% |
| Hepatic calcification | 1 | 0.004% |
| Cerebral hypoperfusion | 1 | 0.004% |
| Brachial plexopathy | 1 | 0.004% |
| Infusion site discolouration | 1 | 0.004% |
| Orthostatic hypertension | 1 | 0.004% |
| Eosinophilic bronchitis | 1 | 0.004% |
| Lewis-Sumner syndrome | 1 | 0.004% |
| Urethral stenosis | 1 | 0.004% |
| Suicidal behaviour | 1 | 0.004% |
| Incision site erythema | 1 | 0.004% |
| Intestinal stent insertion | 1 | 0.004% |
| Peritoneal necrosis | 1 | 0.004% |
| Gastrointestinal stoma complication | 1 | 0.004% |
| Laryngeal inflammation | 1 | 0.004% |
| Lip infection | 1 | 0.004% |
| Peripheral nerve infection | 1 | 0.004% |
| Soft tissue necrosis | 1 | 0.004% |
| Vaginal fistula | 1 | 0.004% |
| Infusion site hypoaesthesia | 1 | 0.004% |
| Intracardiac mass | 1 | 0.004% |
| Allergic transfusion reaction | 1 | 0.004% |
| Metapneumovirus infection | 1 | 0.004% |
| Acute graft versus host disease in skin | 1 | 0.004% |
| Rectal tube insertion | 1 | 0.004% |
| Fear of death | 1 | 0.004% |
| Staphylococcal skin infection | 1 | 0.004% |
| Penile oedema | 1 | 0.004% |
| Scleral buckling surgery | 1 | 0.004% |
| Tricuspid valve prolapse | 1 | 0.004% |
| Muscle flap operation | 1 | 0.004% |
| Procedural nausea | 1 | 0.004% |
| Procedural headache | 1 | 0.004% |
| Retroperitoneal lymphadenopathy | 1 | 0.004% |
| Basal ganglia haemorrhage | 1 | 0.004% |
| Procalcitonin increased | 1 | 0.004% |
| Regurgitation | 1 | 0.004% |
| Vasogenic cerebral oedema | 1 | 0.004% |
| Left atrial dilatation | 1 | 0.004% |
| Hypercreatinaemia | 1 | 0.004% |
| Pharyngeal disorder | 1 | 0.004% |
| Abdominal wall haematoma | 1 | 0.004% |
| Gingival erythema | 1 | 0.004% |
| Haemostasis | 1 | 0.004% |
| Cerebral microangiopathy | 1 | 0.004% |
| Pancreatic neuroendocrine tumour | 1 | 0.004% |
| Segmented hyalinising vasculitis | 1 | 0.004% |
| Cartilage atrophy | 1 | 0.004% |
| Vulvovaginal burning sensation | 1 | 0.004% |
| Gastrointestinal sounds abnormal | 1 | 0.004% |
| Focal segmental glomerulosclerosis | 1 | 0.004% |
| Blood creatine phosphokinase decreased | 1 | 0.004% |
| Haemorrhagic erosive gastritis | 1 | 0.004% |
| Coagulation factor deficiency | 1 | 0.004% |
| Gastrointestinal mucosal exfoliation | 1 | 0.004% |
| Neurosurgery | 1 | 0.004% |
| Renal cell carcinoma | 1 | 0.004% |
| Butterfly rash | 1 | 0.004% |
| Mucosal atrophy | 1 | 0.004% |
| Paranasal sinus mucosal hypertrophy | 1 | 0.004% |
| Cerebral amyloid angiopathy | 1 | 0.004% |
| Inhibitory drug interaction | 1 | 0.004% |
| Thyroid hormone replacement therapy | 1 | 0.004% |
| Metastatic ocular melanoma | 1 | 0.004% |
| Extramammary Paget's disease | 1 | 0.004% |
| Hepatitis B DNA increased | 1 | 0.004% |
| Intestinal metaplasia | 1 | 0.004% |
| Axillary lymphadenectomy | 1 | 0.004% |
| Natural killer cell count decreased | 1 | 0.004% |
| Splenic calcification | 1 | 0.004% |
| Ear infection fungal | 1 | 0.004% |
| Respiratory fatigue | 1 | 0.004% |
| Ligament pain | 1 | 0.004% |
| Administration site abscess | 1 | 0.004% |
| Vaccination site pain | 1 | 0.004% |
| Genital hypoaesthesia | 1 | 0.004% |
| Perianal streptococcal infection | 1 | 0.004% |
| Basal ganglia infarction | 1 | 0.004% |
| Peritonectomy | 1 | 0.004% |
| Atrophic glossitis | 1 | 0.004% |
| Granulomatous pneumonitis | 1 | 0.004% |
| Product contamination physical | 1 | 0.004% |
| Product label confusion | 1 | 0.004% |
| Product packaging quantity issue | 1 | 0.004% |
| Ocular ischaemic syndrome | 1 | 0.004% |
| Atrioventricular dissociation | 1 | 0.004% |
| Paraneoplastic encephalomyelitis | 1 | 0.004% |
| Mesenteritis | 1 | 0.004% |
| Acquired gene mutation | 1 | 0.004% |
| Morganella test positive | 1 | 0.004% |
| Stenotrophomonas test positive | 1 | 0.004% |
| Enterobacter test positive | 1 | 0.004% |
| Enterococcus test positive | 1 | 0.004% |
| Streptococcus test positive | 1 | 0.004% |
| Klebsiella test positive | 1 | 0.004% |
| Proteus test positive | 1 | 0.004% |
| Mycoplasma test positive | 1 | 0.004% |
| Hepatitis C virus test positive | 1 | 0.004% |
| Burning feet syndrome | 1 | 0.004% |
| Human rhinovirus test positive | 1 | 0.004% |
| Coronavirus test positive | 1 | 0.004% |
| Refractory cancer | 1 | 0.004% |
| Enterovirus test positive | 1 | 0.004% |
| Aspergillus test positive | 1 | 0.004% |
| Heparin-induced thrombocytopenia test positive | 1 | 0.004% |
| Lip pruritus | 1 | 0.004% |
| Reactive airways dysfunction syndrome | 1 | 0.004% |
| Infection reactivation | 1 | 0.004% |
| VIth nerve paresis | 1 | 0.004% |
| Enostosis | 1 | 0.004% |
| Injection related reaction | 1 | 0.004% |
| Administration site rash | 1 | 0.004% |
| Infectious thyroiditis | 1 | 0.004% |
| Suspected counterfeit product | 1 | 0.004% |
| Slow speech | 1 | 0.004% |
| Hepatitis B core antibody positive | 1 | 0.004% |
| Vocal cord leukoplakia | 1 | 0.004% |
| Mycobacterium chelonae infection | 1 | 0.004% |
| Exposure via partner | 1 | 0.004% |
| Maternal exposure timing unspecified | 1 | 0.004% |
| Autoimmune aplastic anaemia | 1 | 0.004% |
| Vulvovaginal rash | 1 | 0.004% |
| N-terminal prohormone brain natriuretic peptide increased | 1 | 0.004% |
| Muscle oedema | 1 | 0.004% |
| Noninfectious myelitis | 1 | 0.004% |
| Management of reproduction | 1 | 0.004% |
| Fructose intolerance | 1 | 0.004% |
| Paraneoplastic neurological syndrome | 1 | 0.004% |
| Ocular rosacea | 1 | 0.004% |
| Transfusion-associated dyspnoea | 1 | 0.004% |
| Oesophageal mucosa erythema | 1 | 0.004% |
| Substance-induced psychotic disorder | 1 | 0.004% |
| Oesophageal motility disorder | 1 | 0.004% |
| Desmoplastic melanoma | 1 | 0.004% |
| Periureteral collection | 1 | 0.004% |
| Haemangioma of bone | 1 | 0.004% |
| Hypercapnic coma | 1 | 0.004% |
| Refractory cytopenia with unilineage dysplasia | 1 | 0.004% |
| Lid sulcus deepened | 1 | 0.004% |
| Drain placement | 1 | 0.004% |
| Malignant sweat gland neoplasm | 1 | 0.004% |
| Invasive ductal breast carcinoma | 1 | 0.004% |
| Invasive lobular breast carcinoma | 1 | 0.004% |
| Extraskeletal myxoid chondrosarcoma | 1 | 0.004% |
| Exposure via body fluid | 1 | 0.004% |
| Small intestine polyp | 1 | 0.004% |
| Anaplastic large-cell lymphoma | 1 | 0.004% |
| Acquired acrodermatitis enteropathica | 1 | 0.004% |
| Obstructive shock | 1 | 0.004% |
| Incorrect dosage administered | 1 | 0.004% |
| Radioembolisation | 1 | 0.004% |
| Intentional underdose | 1 | 0.004% |
| Procedural intestinal perforation | 1 | 0.004% |
| Lymph node haemorrhage | 1 | 0.004% |
| Therapy change | 1 | 0.004% |
| Diaphragmatic spasm | 1 | 0.004% |
| Ophthalmic vein thrombosis | 1 | 0.004% |
| Traumatic haemothorax | 1 | 0.004% |
| Graft versus host disease in eye | 1 | 0.004% |
| Eyelid rash | 1 | 0.004% |
| Inferior vena cava stenosis | 1 | 0.004% |
| Monoclonal B-cell lymphocytosis | 1 | 0.004% |
| Meningoencephalitis viral | 1 | 0.004% |
| Noninfective chorioretinitis | 1 | 0.004% |
| Apoptotic colonopathy | 1 | 0.004% |
| Forced vital capacity decreased | 1 | 0.004% |
| Hyperfibrinolysis | 1 | 0.004% |
| Infected seroma | 1 | 0.004% |
| Pancreatic fibrosis | 1 | 0.004% |
| Transplant dysfunction | 1 | 0.004% |
| Crowned dens syndrome | 1 | 0.004% |
| Thyrotoxic cardiomyopathy | 1 | 0.004% |
| Mucosal toxicity | 1 | 0.004% |
| Administration site haematoma | 1 | 0.004% |
| Intestinal intraepithelial lymphocytes increased | 1 | 0.004% |
| Noninfective sialoadenitis | 1 | 0.004% |
| Cerebral ventricular rupture | 1 | 0.004% |
| Hernia perforation | 1 | 0.004% |
| Perineal rash | 1 | 0.004% |
| Coating in mouth | 1 | 0.004% |
| Reactive gastropathy | 1 | 0.004% |
| Cholangitis infective | 1 | 0.004% |
| Cutaneous symptom | 1 | 0.004% |
| Dry age-related macular degeneration | 1 | 0.004% |
| Intestinal sepsis | 1 | 0.004% |
| BRAF V600E mutation positive | 1 | 0.004% |
| Basophilopenia | 1 | 0.004% |
| Macular detachment | 1 | 0.004% |
| Behaviour disorder due to a general medical condition | 1 | 0.004% |
| Idiopathic orbital inflammation | 1 | 0.004% |
| Medical device site joint infection | 1 | 0.004% |
| Disorganised speech | 1 | 0.004% |
| Hepatic hypertrophy | 1 | 0.004% |
| Fine motor skill dysfunction | 1 | 0.004% |
| Chronic disease | 1 | 0.004% |
| Steatohepatitis | 1 | 0.004% |
| Large intestine erosion | 1 | 0.004% |
| Gastrointestinal mucosa hyperaemia | 1 | 0.004% |
| Synovial cyst removal | 1 | 0.004% |
| Erythematotelangiectatic rosacea | 1 | 0.004% |
| Urinary tract discomfort | 1 | 0.004% |
| Eye paraesthesia | 1 | 0.004% |
| Lumbosacral radiculopathy | 1 | 0.004% |
| Infective keratitis | 1 | 0.004% |
| New daily persistent headache | 1 | 0.004% |
| Campylobacter colitis | 1 | 0.004% |
| Dose calculation error | 1 | 0.004% |
| Cerebral congestion | 1 | 0.004% |
| Impaired quality of life | 1 | 0.004% |
| Vein rupture | 1 | 0.004% |
| Systemic infection | 1 | 0.004% |
| Central nervous system melanoma | 1 | 0.004% |
| Intensive care unit acquired weakness | 1 | 0.004% |
| Adult failure to thrive | 1 | 0.004% |
| Non-cirrhotic portal hypertension | 1 | 0.004% |
| Immunoglobulin G4 related disease | 1 | 0.004% |
| Hereditary motor and sensory neuropathy | 1 | 0.004% |
| Tumour obstruction | 1 | 0.004% |
| Cytokine abnormal | 1 | 0.004% |
| Imaging procedure abnormal | 1 | 0.004% |
| Device related thrombosis | 1 | 0.004% |
| Myeloproliferative neoplasm | 1 | 0.004% |
| Procedural pneumothorax | 1 | 0.004% |
| Spinal flattening | 1 | 0.004% |
| Quadrantanopia | 1 | 0.004% |
| Gingival discomfort | 1 | 0.004% |
| Tongue discomfort | 1 | 0.004% |
| Herpes simplex test positive | 1 | 0.004% |
| Selective eating disorder | 1 | 0.004% |
| Somatic symptom disorder | 1 | 0.004% |
| Gastrointestinal bacterial overgrowth | 1 | 0.004% |
| Corticobasal degeneration | 1 | 0.004% |
| Discontinued product administered | 1 | 0.004% |
| Therapeutic response shortened | 1 | 0.004% |
| Manufacturing materials contamination | 1 | 0.004% |
| Thyroid gland injury | 1 | 0.004% |
| Hemiparaesthesia | 1 | 0.004% |
| Tumour inflammation | 1 | 0.004% |
| Oxygen therapy | 1 | 0.004% |
| Idiopathic intracranial hypertension | 1 | 0.004% |
| Cerebellar stroke | 1 | 0.004% |
| Tongue erythema | 1 | 0.004% |
| Intercepted product storage error | 1 | 0.004% |
| Product advertising issue | 1 | 0.004% |
| Drug effective for unapproved indication | 1 | 0.004% |
| Herpes zoster meningoradiculitis | 1 | 0.004% |
| Drug use disorder | 1 | 0.004% |
| Epstein Barr virus positive mucocutaneous ulcer | 1 | 0.004% |
| Drain site complication | 1 | 0.004% |
| Dependence on oxygen therapy | 1 | 0.004% |
| Palate injury | 1 | 0.004% |
| Eye haematoma | 1 | 0.004% |
| Cryptitis | 1 | 0.004% |
| Urea cycle disorder | 1 | 0.004% |
| Symptom recurrence | 1 | 0.004% |
| Product communication issue | 1 | 0.004% |
| Skin lesion removal | 1 | 0.004% |
| Drug specific antibody | 1 | 0.004% |
| Product dispensing error | 1 | 0.004% |
| Animal attack | 1 | 0.004% |
| Sinonasal obstruction | 1 | 0.004% |
| Skin weeping | 1 | 0.004% |
| Vascular device infection | 1 | 0.004% |
| Counterfeit product administered | 1 | 0.004% |
| Incorrect dose administered by product | 1 | 0.004% |
| Tissue irritation | 1 | 0.004% |
| Tissue rupture | 1 | 0.004% |
| Ulcerative duodenitis | 1 | 0.004% |
| Respiratory tract procedural complication | 1 | 0.004% |
| Ciliary body melanoma | 1 | 0.004% |
| Uveal melanoma | 1 | 0.004% |
| Autoimmune enteropathy | 1 | 0.004% |
| Poor quality product administered | 1 | 0.004% |
| Stiff tongue | 1 | 0.004% |
| Complicated appendicitis | 1 | 0.004% |
| Wrong product administered | 1 | 0.004% |
| Intercepted product prescribing error | 1 | 0.004% |
| Sitting disability | 1 | 0.004% |
| Hypotony maculopathy | 1 | 0.004% |
| Anti-glomerular basement membrane disease | 1 | 0.004% |
| Drug level abnormal | 1 | 0.004% |
| Therapeutic product effect increased | 1 | 0.004% |
| Pharyngeal swelling | 1 | 0.004% |
| Pulmonary imaging procedure abnormal | 1 | 0.004% |
| Disruption of the photoreceptor inner segment-outer segment | 1 | 0.004% |
| Insulin-like growth factor abnormal | 1 | 0.004% |
| Cerebral venous sinus thrombosis | 1 | 0.004% |
| Urinary tract candidiasis | 1 | 0.004% |
| SJS-TEN overlap | 1 | 0.004% |
| HER2 negative breast cancer | 1 | 0.004% |
| Lymph node ulcer | 1 | 0.004% |
| Drug effect less than expected | 1 | 0.004% |
| Oral lichenoid reaction | 1 | 0.004% |
| Diversion proctitis | 1 | 0.004% |
| Loss of therapeutic response | 1 | 0.004% |
| SARS-CoV-2 test negative | 1 | 0.004% |
| Diverticulitis intestinal perforated | 1 | 0.004% |
| Exposure to SARS-CoV-2 | 1 | 0.004% |
| TP53 gene mutation | 1 | 0.004% |
| SARS-CoV-2 sepsis | 1 | 0.004% |
| Troponin decreased | 1 | 0.004% |
| Troponin abnormal | 1 | 0.004% |
| Peritoneal catheter insertion | 1 | 0.004% |
| Postoperative lymphocele | 1 | 0.004% |
| Retroperitoneal disorder | 1 | 0.004% |
| Myelin oligodendrocyte glycoprotein antibody-associated disease | 1 | 0.004% |
| Idiopathic inflammatory myopathy | 1 | 0.004% |
| Central serous chorioretinopathy | 1 | 0.004% |
| Squamoproliferative lesion | 1 | 0.004% |
| Immune-mediated scleritis | 1 | 0.004% |
